# Rewiring of an Epithelial Differentiation Factor, miR-203, to Inhibit Human Squamous Cell Carcinoma Metastasis

**DOI:** 10.1016/j.celrep.2014.08.062

**Published:** 2014-10-02

**Authors:** Nathan Benaich, Samuel Woodhouse, Stephen J. Goldie, Ajay Mishra, Sven R. Quist, Fiona M. Watt

**Affiliations:** 1 Cancer Research UK Cambridge Institute, University of Cambridge, Li Ka Shing Centre, Robinson Way, Cambridge CB2 0RE, UK; 2 Centre for Stem Cells and Regenerative Medicine, King’s College London, 28^th^ Floor, Tower Wing, Guy’s Hospital, Great Maze Pond, London SE1 9RT, UK; 3 Clinic of Dermatology and Venereology, Otto-von-Guericke University, Magdeburg, Leipziger Strasse 44, 39120 Magdeburg, Germany

## Abstract

Metastatic colonization of distant organs underpins the majority of human-cancer-related deaths, including deaths from head and neck squamous cell carcinoma (HNSCC). We report that miR-203, a miRNA that triggers differentiation in multilayered epithelia, inhibits multiple postextravasation events during HNSCC lung metastasis. Inducible reactivation of miR-203 in already established lung metastases reduces the overall metastatic burden. Using an integrated approach, we reveal that miR-203 inhibits metastasis independently of its effects on differentiation. In vivo genetic reconstitution experiments show that miR-203 inhibits lung metastasis by suppressing the prometastatic activities of three factors involved in cytoskeletal dynamics (LASP1), extracellular matrix remodeling (SPARC), and cell metabolism (NUAK1). Expression of miR-203 and its downstream effectors correlates with HNSCC overall survival outcomes, indicating the therapeutic potential of targeting this signaling axis.

## Highlights

• miR-203 regulates in vivo lung metastasis without triggering differentiation
• Restoring miR-203 in already established metastases elicits regression
• LASP1, NUAK1, and SPARC are downstream prometastatic effectors of miR-203
• The miR-203-LASP1/SPARC/NUAK1 axis is prognostic of overall survival in HNSCC

## Graphical Abstract

**Figure:**
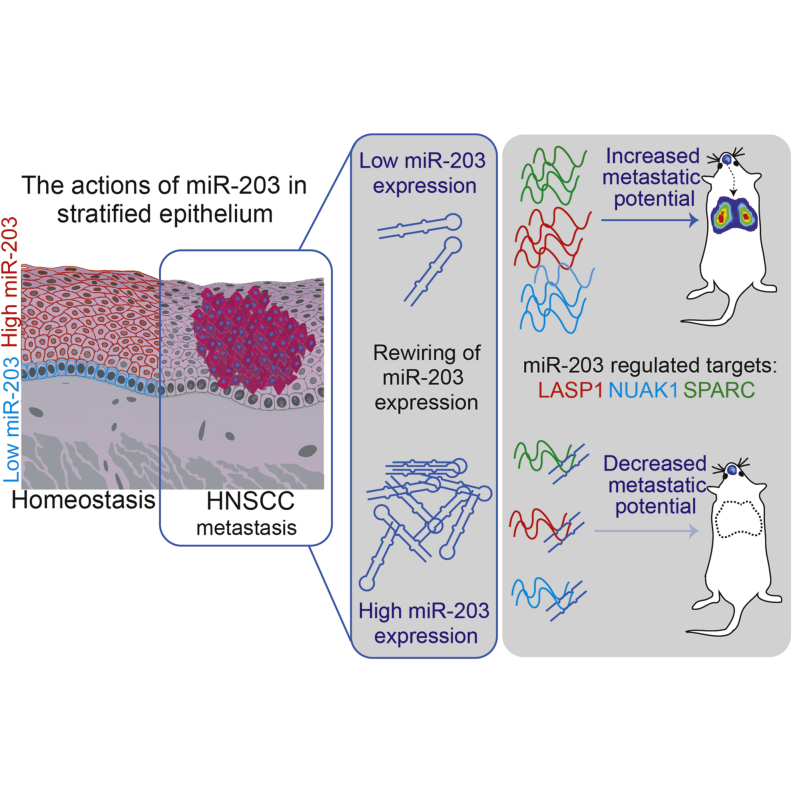

## Teaser

Benaich et al. have identified miR-203, a microRNA that triggers differentiation in multilayered epithelia, as an inhibitor of lung metastasis in head and neck squamous cell carcinoma (HNSCC) cells. They show that miR-203 inhibits metastasis independently of its effects on differentiation. Rather, miR-203 suppresses the prometastatic activities of three factors involved in cytoskeletal dynamics (LASP1), extracellular matrix remodeling (SPARC), and cell metabolism (NUAK1). Expression of miR-203 and its downstream effectors correlates with survival in HNSCC patients.

## Introduction

Adult stratified epithelia are maintained by a balance between stem cell self-renewal and differentiation (Arwert et al., 2012; Blanpain and Fuchs, 2006). As emerging neoplastic drivers, stem cells and the factors that control their biology are of therapeutic relevance in carcinomas (Pardal et al., 2003). For example, *NOTCH1* and *TP63* form a negative feedback loop in epidermal stem cells, with *NOTCH1* promoting differentiation and *TP63* inhibiting it (Nguyen et al., 2006). Inactivation of these genes is associated with skin tumors in mice (Flores et al., 2005; Nicolas et al., 2003) and head and neck squamous cell carcinoma (HNSCC) in humans (Agrawal et al., 2011; Stransky et al., 2011). Thus, disruption of the epithelial stem cell molecular circuitry can play a driving role in malignant transformation of the tissues they replenish.

HNSCC is the sixth most common cancer worldwide and has had a 5-year overall survival rate of only ∼50% for decades (Leemans et al., 2011). Two-thirds of patients present with advanced, locally invasive disease that recurs despite mainstay surgery or chemo- and/or radiotherapy, thus creating a pressing need for novel avenues of therapeutic intervention (Argiris et al., 2008).

Metastasis accounts for >90% of solid-cancer-related deaths (Valastyan and Weinberg, 2011). Metastatic dissemination can occur early in the evolution of a tumor, followed by extended dormancy (Hüsemann et al., 2008). Indeed, up to 40% of carcinoma cases without clinical evidence of metastasis actually harbor disseminated tumor cells in the bone marrow (Pantel and Brakenhoff, 2004). Thus, truly efficacious cancer therapeutics must target already established metastases rather than just inhibit tumor growth or dissemination (Valastyan and Weinberg, 2011).

miRNAs are small noncoding RNAs that posttranscriptionally repress target mRNAs important for tissue homeostasis and cancer (Lujambio and Lowe, 2012; Valastyan et al., 2009b). Although our understanding of metastasis-relevant miRNAs has advanced rapidly in well-studied malignancies such as breast cancer (Valastyan et al., 2009a, 2010, 2011; Yi et al., 2008), we know little about whether and how miRNAs modulate metastasis in HNSCC. Therefore, we employed functional in vivo approaches to identify miR-203 as a potent negative regulator of HNSCC metastasis by targeting a panel of prometastatic effector proteins (Yi et al., 2008).

## Results

### A Screen of miRNAs in HNSCC Identifies miR-203 as a Metastasis Suppressor

**Figure 1:**
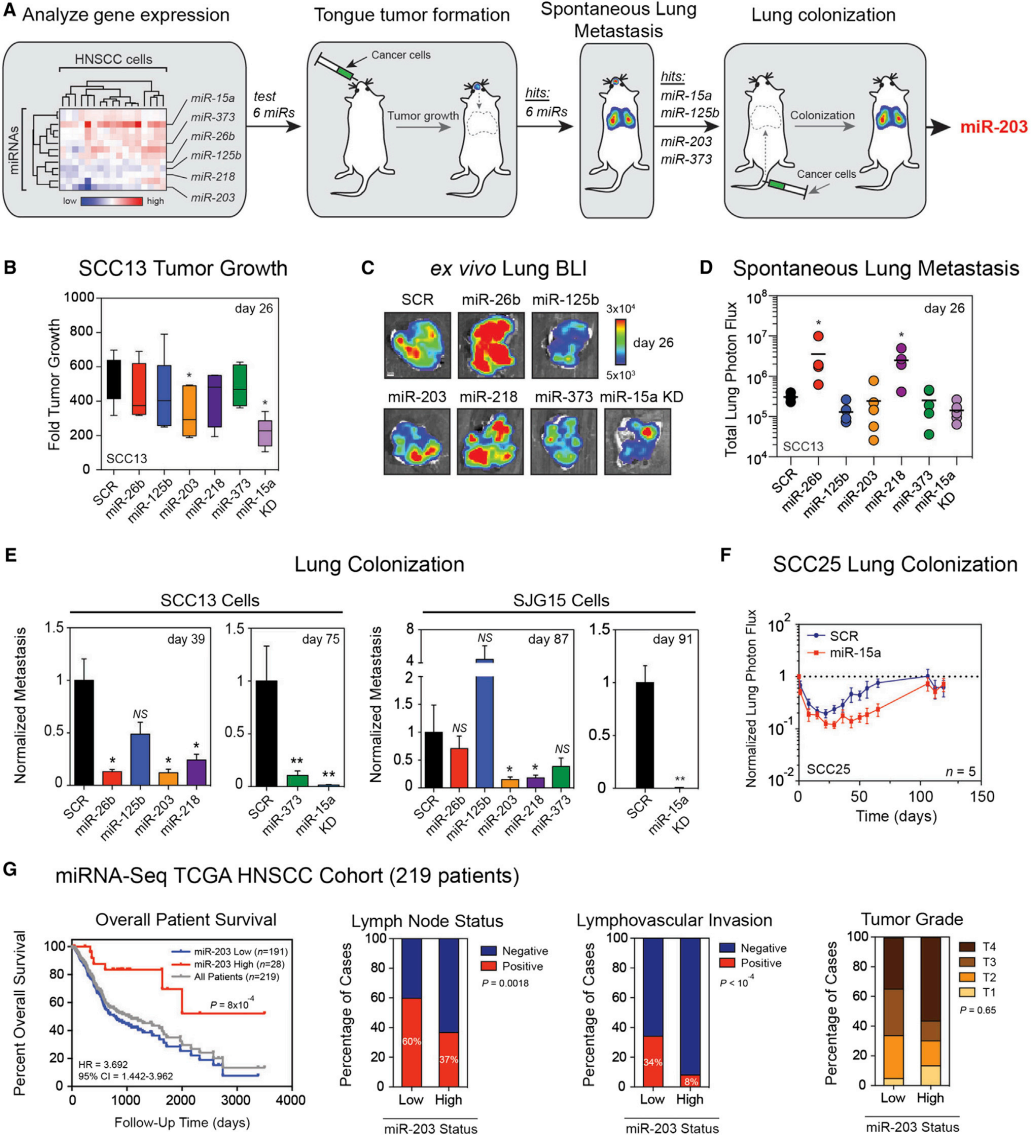
Candidate-Gene-Based Functional In Vivo miRNA Screen (A) Schematic of the pipeline for an in vivo functional screen to identify miRNAs that regulate HNSCC lung metastasis. Heatmap of log_2_ normalized qRT-PCR expression data for 13 miRNAs in 17 human HNSCC lines normalized to normal human oral keratinocytes. Data were clustered using cosine statistics. (B) Fold primary tumor growth generated by 10^5^ SCC13 cells individually expressing the indicated miRNA vectors after 26 days. Whiskers indicate min/max and the horizontal bar is the median, with n = 4–5 per group. (C) Representative ex vivo bioluminescent images of whole lungs at necropsy (day 26). Scale bar represents 3 mm. (D) Total ex vivo lung photon flux at endpoint (day 26). The horizontal line indicates mean, with n = 5 per group. (E) Lung metastatic burden resulting from tail-vein injection of SCC13 or SJG15 cells in which the levels of six miRNAs were individually modulated. Data are means ± SEM, n = 5 per group. (F) Time course of experimental lung metastasis by SCC25 cells overexpressing miR-15a or control vector for 119 days. Log_10_ y axis, data are means ± SEM, n = 5 per group. (G) Kaplan-Meier plots for overall survival of 219 HNSCC patients from the TCGA cohort partitioned into miR-203-high (red, n = 28) and miR-203-low (blue, n = 191) cohorts based on miRNAseq read counts. The miR-203-high and -low patient cohorts were analyzed for the proportion of cases with lymph node disease (positive or negative), lymphovascular invasion (positive or negative), and primary tumor T-stage, which is a measure of tumour state and size. ^∗^p < 0.05; ^∗∗^p < 0.01; ^∗∗∗^p < 0.001 (nonparametric Mann-Whitney test). For Kaplan-Meier plots, p values were calculated using a log rank Mantel-Cox test. For patient cohort comparisons, p values were calculated using Fisher’s exact test. See also Figure S1.

To uncover endogenous miRNAs that reduce the lung metastatic potential of HNSCC, we employed the screening approach shown in Figure 1A. Using a panel of 17 primary, early-passage human HNSCC cell lines from surgically resected tumors, we assayed the expression of 15 miRNAs identified as coordinately deregulated in published expression profiles of HNSCC (see the Supplemental Experimental Procedures). We identified five downregulated miRNAs (miR-26b, miR-125b, miR-203, miR-218, and miR-373) and one upregulated miRNA (miR-15a) when we compared miRNA expression in HNSCC cells versus primary human keratinocytes (Figure 1A). miR-133a and miR-133b were not detected in any lines.

To assess the function of the deregulated miRNAs in HNSCC, we generated two YFP-luciferase-expressing cell lines—SCC13 (established facial SCC; Rheinwald and Beckett, 1981) and SJG15 (primary lingual SCC; Goldie et al., 2012)—in which we knocked down miR-15a or stably overexpressed miR-26b, miR-125b, miR-203, miR-218, or miR-373 using lentiviral approaches (Figure S1A). No antiproliferative or cytotoxic effects were observed in vitro in adherent cultures (Figure S1C).

We explored the function of these miRNAs in an in vivo orthotopic tongue xenograft assay that recapitulates aspects of human HNSCC (Goldie et al., 2012). SCC13 cells were injected into the lingual mucosa of nonobese diabetic/severe combined immunodeficient interleukin-2 receptor-γ chain null (NSG) mice (Figure 1A), and noninvasive, luciferase-based bioluminescent imaging was used to monitor disease progression (Kim et al., 2010; Tiffen et al., 2010). Xenografting 10^5^ miRNA expressing SCC13 cells revealed that overexpression of miR-203 and knockdown of miR-15a modestly reduced tumor burden after 26 days compared with control. The other miRNAs did not affect tumor burden (Figures 1B and S1D).

To measure metastatic dissemination from the primary tumors, we imaged the lungs of tumor-bearing mice ex vivo at 26 days (Figure 1C). Overexpression of miR-26b and miR-218 enhanced metastatic dissemination, whereas the other four miRNAs had no significant effect (Figure 1D). Since the aim of the screen was to identify antimetastatic miRNAs, we did not analyze miR-26b and miR-218 further.

To examine metastatic colonization independently of primary tumor growth, we intravenously injected miRNA-expressing SCC13 and SJG15 cells into NSG mice (Figure 1A). Overexpression of miR-203 or knockdown of miR-15a suppressed metastasis in both cell lines, as determined by endpoint lung metastatic burden, ex vivo fluorescence microscopy, and histology (Figures 1E and S1E–S1H). miR-125b and miR-373 were excluded from further analysis because their effects were not consistent between SCC13 and SJG15 cells. Since miR-15a is a tumor suppressor (Aqeilan et al., 2010), we expressed miR-15a in otherwise nonmetastatic, miR-15a^low^ SCC25 cells (established lingual SCC; Rheinwald and Beckett, 1981; Figure S1B). Overexpression of miR-15a did not enhance lung metastatic colonization by SCC25 cells (Figure 1F), so we did not analyze this miR further.

With miR-203 presenting the most compelling case as a metastasis-suppressor gene, we explored its clinical relevance in a cohort of 219 HNSCC patients from The Cancer Genome Atlas (TCGA) (data available through the TCGA Data Portal; Figure 1G). The 5-year survival rate of miR-203-low patients was 36%, compared with 70% for miR-203-high patients, independently of primary tumor size (T1/T2 versus T3/T4). Patients with miR-203-low tumors also exhibited significantly more lymph node-positive disease and lymphovascular invasion—two major prognostic factors for eventual distant metastasis. Hence, miR-203 is a prognostic indicator of overall survival and propensity for metastasis.

Using high-resolution copy-number data for 322 HNSCC patients from TCGA, we detected a copy-number alteration (CNA) harboring miR-203 that was focally deleted in 15% of cases (Figure S2B). This CNA included several genes that have been implicated in suppressing epithelial tumorigenesis and/or metastasis, such as DICER1 (Martello et al., 2010) and ELF5 (Chakrabarti et al., 2012). The miR-203 locus was also deleted in cutaneous melanoma (34% of cases), lung adenocarcinoma (29% of cases), and glioblastoma multiforme (28% of cases) (Figure S2C). Therefore, deregulation of miR-203 in HNSCC (and potentially other tumor types) is due, at least in part, to focal CNAs that alter multiple tumor- and/or metastasis-suppressive pathways.

### Effect of miR-203 Expression on Primary Tumor Growth

**Figure 2:**
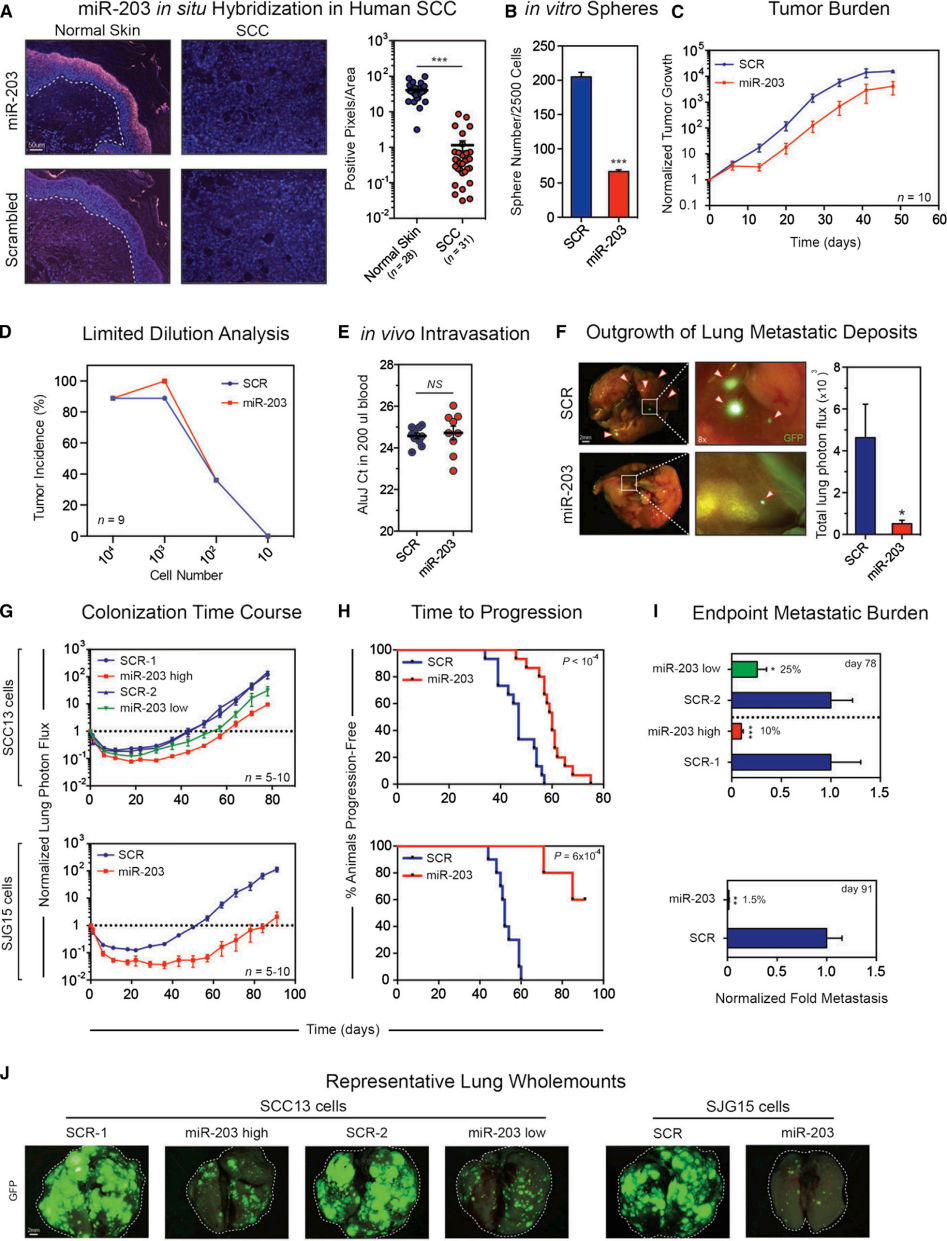
miR-203 Inhibits Experimental Lung Metastasis (A) Representative in situ hybridization (red signal) for miR-203 and scrambled control probe in normal human skin (n = 28) and malignant SCC (n = 31) counterstained with DAPI nuclear label. Scale bar represents 50 μm. Adjacent panel: quantification of miR-203 signal intensity in normal skin versus SCC. Log_10_ y axis. (B) Anchorage-independent soft-agar assay. GFP^+^ spheres were quantified after 3 weeks. Data are means ± SEM. (C) Fold primary tumor growth: 10^4^ control and miR-203 expressing SCC13 cells were injected into the tongue and tumor growth was monitored by bioluminescent imaging. Raw photon flux was normalized to input levels and presented as mean ± SEM; n = 10 per group. (D) Limited dilution analysis: 10^4^, 10^3^, 100, and 10 control and miR-203-expressing SCC13 cells were injected into the tongue. Tumor incidence (5-fold increase over baseline) was determined by bioluminescent imaging and tumors were monitored for up to 70 days (n = 9). (E) In vivo tumor cell intravasation was inferred by using qRT-PCR for human-specific AluJ repeat elements present in circulating tumor cells within 200 μl whole blood. n = 10 (SCR), n = 9 (miR-203). (F) Whole-mount lung fluorescence images from representative animals in (D). Scale bar represents 2 mm; left panels at 2× and right panels at 8× magnification. Quantification of lung metastasis by bioluminescence; data are means ± SEM; n = 7 (SCR), n = 6 (miR-203). (G) Lung metastatic colonization time course for SCC13 cells expressing high (n = 10) or low miR-203 (n = 5) and relevant controls, and SJG15 cells expressing miR-203 or scrambled control hairpin (n = 5 each). Log_10_ y axis. Data are means ± SEM. (H) Kaplan-Meier analysis for time to progression of lung metastasis. SCC13 SCR (n = 15) and miR-203 groups (high and low; n = 15) were pooled. (I) Endpoint lung metastatic burden of animals from (F) injected with miR-203 expressing SCC13 cells (day 78) and SJG15 cells (day 91) normalized to control animals. Data are means ± SEM. (J) Representative whole-mount GFP fluorescence microscopy images of metastatic lung fields generated by miR-203 and control SCC13 and SJG15 cells. Scale bar represents 2 mm. ^∗^p < 0.05; ^∗∗^p < 0.01; ^∗∗∗^p < 0.001 calculated using the nonparametric Mann-Whitney test. The p values for the Kaplan-Meier plots were calculated using the log rank Mantel-Cox test. The p values for the soft-agar assay were calculated using the two-tailed Student’s t test. See also Figures S2 and S3.

To examine miR-203 expression in vivo, we performed in situ hybridization in 28 cases of primary and/or metastatic skin SCCs with matched normal skin from the same patients, and three further cases of skin SCC (total of 11 patients; Table S1). Whereas miR-203 was strongly expressed in the suprabasal, differentiated layers of normal epidermis, confirming previous reports (Yi et al., 2008), its expression was 35-fold lower in matched cutaneous SCCs (Figure 2A).

We found that miR-203 significantly reduced both the average number and size of spheres formed by SCC13 in soft agar (Figure 2B and data not shown), consistent with the observation that when 10^5^ cells were injected into the tongue, miR-203 reduced tumor growth (Figure 1B). To assess the impact on tumor growth in more detail, we xenografted 10^4^ SCC13 cells expressing miR-203 or scrambled control hairpin (SCR) and monitored tumors for up to 50 days (Figure 2C). We confirmed miR-203 expression by in situ hybridization (Figure S3B). The reduction in tumor size upon expression of miR-203 was due to a lag in the initial rate of growth, and from week 2 onward the rate of tumor growth was comparable to that in control SCC13 cells (Figures 2C and S3A).

To investigate whether miR-203 affected the proportion of cells that were capable of initiating primary tumors, we performed a limiting dilution analysis (LDA) (Lapouge et al., 2012) in which 10^4^, 10^3^, 100, or 10 SCC13 SCR or miR-203 cells were injected into the tongue (Figure 2D). No difference in tumor incidence was observed between miR-203- and SCR-expressing cells (Figure 2D). There was, however, a lag in tumor growth phase at all seeding densities (Figures 2C and S1D; data not shown).

We conclude that miR-203 overexpression does not affect the number of tumor-initiating cells or the overall tumor growth rate, but causes a delay before tumor growth becomes established, which results in a reduction in tumor size. This may explain the lack of a correlation between T stage and miR-203 expression in HNSCC patients, since, at least in xenografts, the effect on tumor size is more marked at early time points (Figure 1G).

### miR-203 Expression Inhibits Lung Metastasis

We measured the circulating tumor DNA content by extracting whole peripheral blood 50 days after tongue xenografting (Figure 2C) and determined the levels of human *AluJ* or *hLine1* repeat elements by quantitative PCR (qPCR) (Figure 2E). There was no difference in circulating tumor DNA content between mice bearing miR-203 or control SCC13 primary tumors (Figures 2E and S3C). However, ex vivo bioluminescence and fluorescence imaging of lungs showed that miR-203-expressing SCC13 cells founded smaller metastases than control cells (Figure 2F). Therefore, miR-203 does not alter the ability of cells from the primary tumor to enter the circulation, but does hamper their capacity to expand following engraftment in the lungs.

Consistent with these results, miR-203-expressing SCC13 and SJG15 cells exhibited a dose-dependent impairment in lung colonization following tail-vein injection (Figure 2G). Expression of miR-203 significantly prolonged the time required for SCC13 and SJG15 cells to reach input levels of lung bioluminescence, suggesting that miR-203 extended the dormancy phase of engrafted cells (Figure 2G), mirroring the phenotype observed in primary tumors (Figure 2C). Expression of miR-203 significantly increased survival time (Figure 2H) and reduced the endpoint lung metastatic burden (Figure 2I). This was confirmed by ex vivo fluorescence microscopy and anti-keratin-14 immunohistochemistry (IHC) of lungs (Figure 2J and S3E). Whereas control SCC13 and SJG15 cells colonized the majority of the lung area, cells expressing miR-203 only generated small metastatic lesions or remained as clusters of individual cells (Figure S3F). We confirmed that miR-203 expression was sustained in SCC13 lung metastases (Figure S3G). Taken together, these data suggest that miR-203 regulates the outgrowth of tumor cells once they have reached the lung.

**Figure 3:**
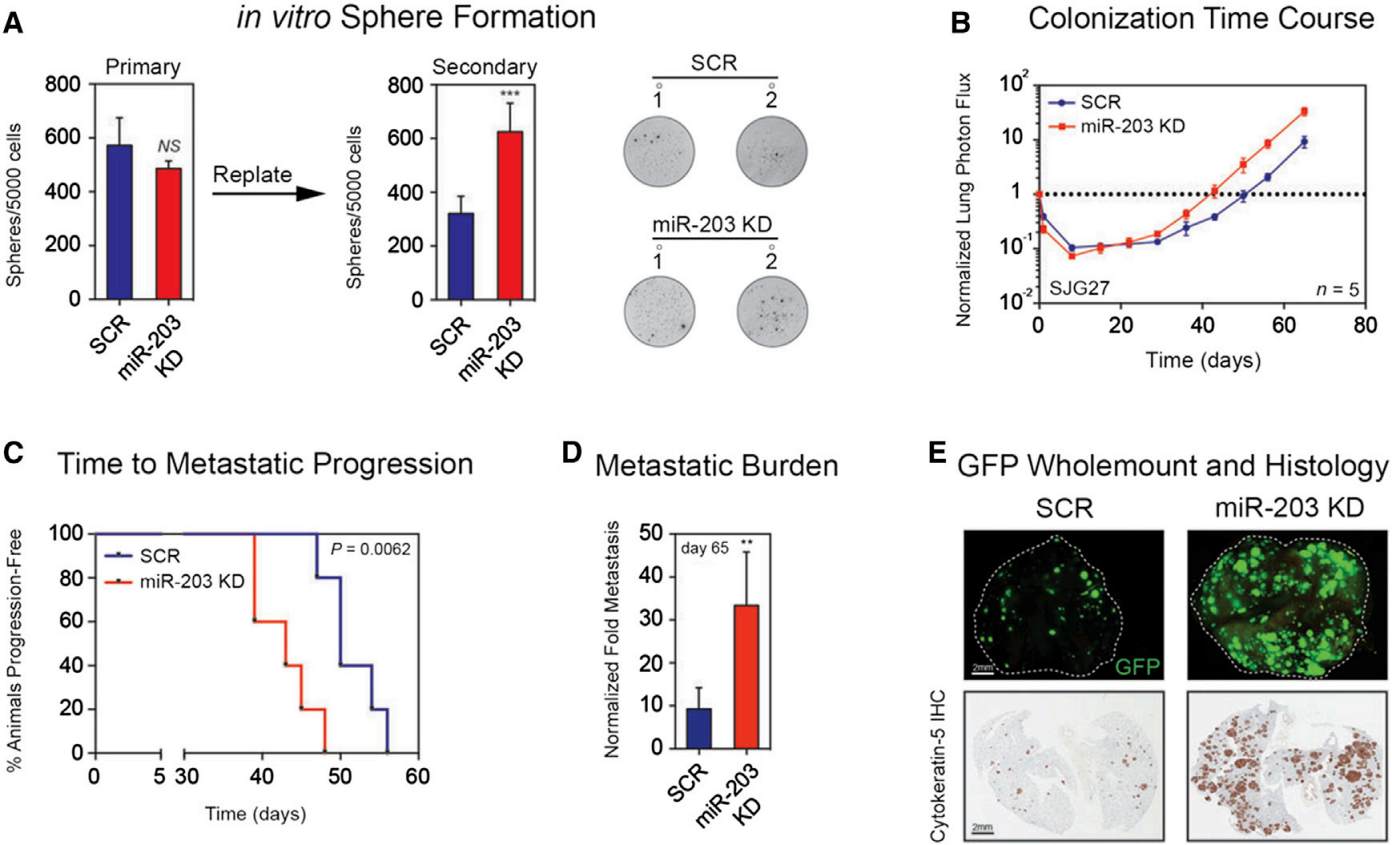
Silencing Endogenous miR-203 Enhances Lung Metastasis (A) In vitro soft-agar assay quantified at the first and second passages. Data are means ± SEM, with representative wells shown; ^∗∗∗^p < 0.001 calculated using two-tailed Student’s t test. (B) Time course of experimental lung metastasis of miR-203 knockdown (KD) and control SJG27 cells over 65 days. Log_10_ y axis; data are mean ± SEM; n = 5 per group. (C) Kaplan-Meier plots for time to progression of lung metastasis of SJG27 SCR and miR-203-KD groups; p values were calculated using the log rank Mantel-Cox test. (D) Lung metastasis by control and miR-203-KD SJG27 cells at day 65. Data are mean ± SEM; n = 5 per group; ^∗∗^p < 0.01 calculated using the nonparametric Mann-Whitney test. (E) Representative whole-mount GFP fluorescence microscopy images and matched keratin-5 IHC of metastatic lungs. Scale bar represents 2 mm. See also Figure S3.

We next asked whether endogenous miR-203 was necessary to prevent metastasis. For this purpose, we used SJG27 cells (primary lingual SCC), which express high levels of miR-203 and do not readily metastasize. Silencing miR-203 (miR-203 KD) in YFP-luciferase-labeled SJG27 cells did not impair proliferation in vitro (Figures S3H and S3I), but did enhance formation of clonogenic spheres in soft agar (Figure 3A). When injected into the circulation of mice, miR-203 KD SJG27 cells were significantly more proficient at forming lung metastases over 65 days compared with control cells (Figure 3B). Knockdown of miR-203 reduced the time to metastatic progression (Figure 3C) and enhanced the total lung metastatic burden (Figure 3D). Ex vivo fluorescence microscopy and anti-keratin-5 IHC showed that miR-203 KD cells generated more numerous and larger nodules than control cells (Figure 3E). Clusters of individual cells were visible in control, but not miR-203 KD, lungs, further suggesting a role for miR-203 in maintaining dormancy (Figure S3J).

We conclude, on the basis of miR-203 knockdown and overexpression, that miR-203 inhibits lung metastasis of HNSCC.

### miR-203 Regulates Multiple Postextravasation Events and Inhibits Established Metastases

**Figure 4:**
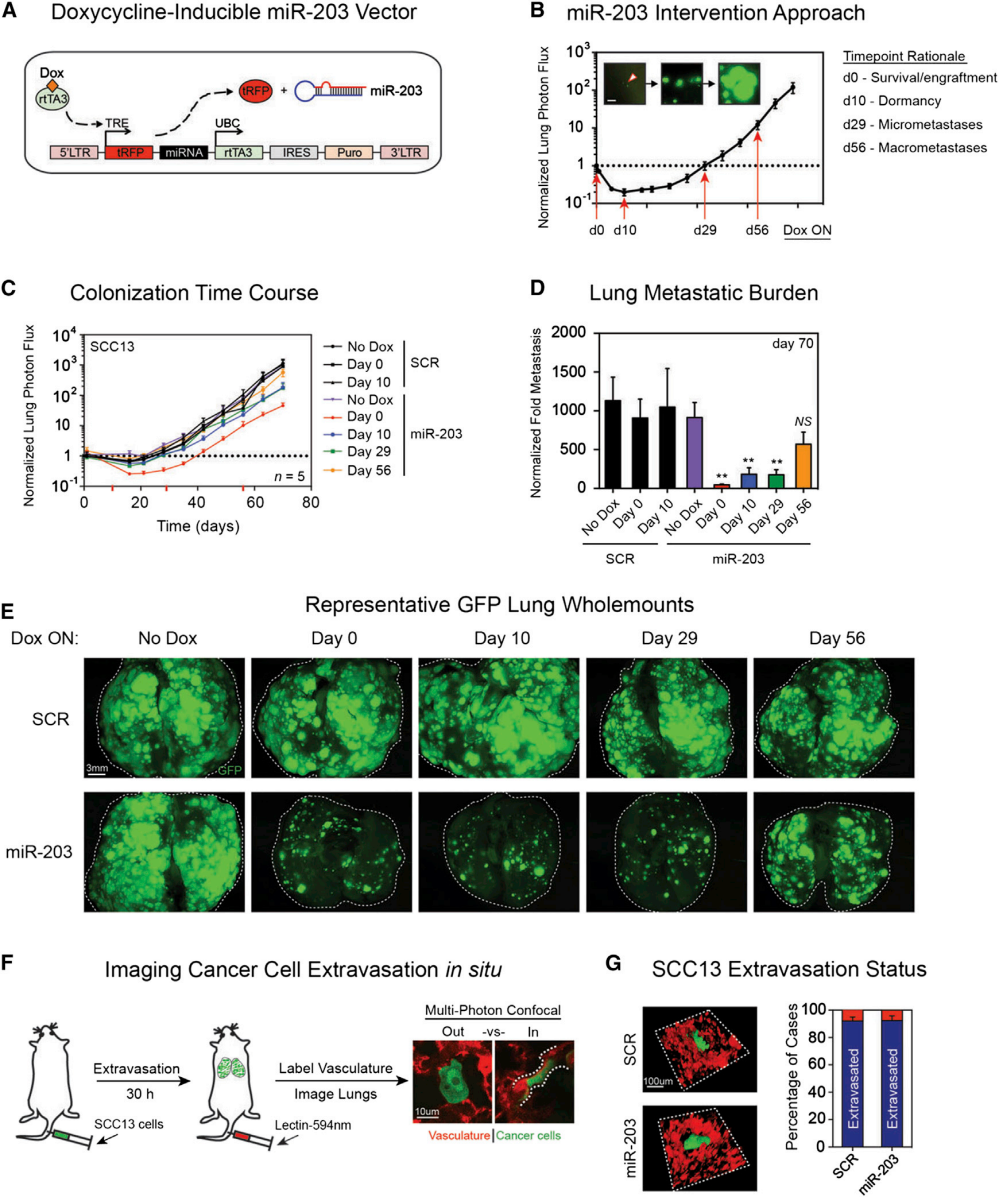
Inducible Restoration of miR-203 (A) Schematic representation of the dox-inducible miR-203 vector. (B) Dox-mediated approach for reexpressing miR-203 following intravenous inoculation of SCC13 cells. Images represent single cells, micrometastases, and macrometastases (left to right) in the lung following tail-vein injection. Scale bar represents 500 μm. (C) Time course of experimental lung metastases formed by inducible control (SCR) and miR-203-expressing SCC13 in animals on normal or dox-rich diets. Day indicates when the diet was switched to dox-rich. Log_10_ y axis; data are means ± SEM; n = 4–5 per group. (D) Lung metastasis at endpoint (day 70) of noninduced or induced control and miR-203 SCC13 groups. Data are means ± SEM; n = 4–5 per group; ^∗∗^p < 0.01 calculated using nonparametric Mann-Whitney test relative to noninduced miR-203 animals. Differences between SCR groups and noninduced miR-203 are nonsignificant. (E) Representative whole-mount ex vivo GFP fluorescence microscopy images of metastatic lungs at day 70. Scale bar represents 3 mm. (F) Experimental strategy to quantify cancer cell extravasation across the lung vasculature in vivo. Examples of GFP^+^ SCC13 cells that have successfully extravasated (out) or are trapped within the lumen of pulmonary blood vessels labeled in red (in) and outlined with a dashed white line are shown. Scale bar represents 10 μm. (G) Proportion of cells in or out of lung vasculature at 30 hr after intravenous injection. Data are mean % ± SEM, with n = 3 per group. A total of 127–136 cells were counted per mouse. See also Figure S4.

We next used a doxycycline (dox)-inducible system to temporally control miR-203 expression (Figures 4A, 4B, and S4A). Dox-inducible miR-203 expression did not affect in vitro proliferation of adherent cells (Figure S4B), but reduced the anchorage-independent growth of SCC13 cells in soft agar (Figure S4C), consistent with the effect of constitutive expression (Figure 2B).

We activated miR-203 expression at defined time points following intravenous injection of SCC13 cells (Figure 4B). Expression of miR-203 was either never induced (mimicking cells lacking miR-203), induced before intravenous injection (day 0, phenocopying constitutive miR-203 expression), or induced 10 (dormancy phase of engrafted cells), 29 (establishment of micrometastases), or 56 days (macrometastatic colonization) after injection.

Animals injected with scrambled control cells that were never induced or induced from day 0, 10, 29, or 56 all had similar disease burdens, arguing against any effects of the dox-rich diet (Figures 4C–4E and data not shown). An examination of RFP and GFP signals from lung metastases in each cohort of animals, as well as in situ hybridization for miR-203, confirmed dox-dependent, sustained expression of miR-203 in vivo (Figures S4D–S4F and data not shown).

Activation of miR-203 for the duration of the experiment resulted in a 95% inhibition of endpoint lung metastatic burden (Figures 4C and 4D). Reexpression of miR-203 from 10 or 29 days was sufficient to block 80% of the endpoint lung metastatic burden (Figures 4C and 4D). Ex vivo fluorescence microscopy of lungs confirmed that reintroduction of miR-203 substantially reduced the number and size of lung metastases (Figure 4E). Although it was not statistically significant, animals in which miR-203 was induced for only the final 2 weeks of the experiment had ∼40% lower lung metastasis (Figures 4C and 4D) accompanied by a moderate reduction in lesion size and number (Figure 4E).

We next investigated whether miR-203 affects cancer cell extravasation across the lung vasculature in vivo (Figure 4F). Thirty hours after intravenous injection, multiphoton confocal imaging of individual GFP^+^ tumor cells and tomato lectin-labeled blood vessels revealed that a similar proportion (∼90%) of control and miR-203-expressing SCC13 cells had successfully extravasated (Figure 4G).

We conclude that miR-203 inhibits lung metastasis by inhibiting exit from dormancy, rather than by preventing migration from the primary tumor or entry into the lung.

### miR-203 Induces Mutually Exclusive Transcriptomic Landscapes Characteristic of Keratinocyte Differentiation and Poorly Metastatic Cells

**Figure 5:**
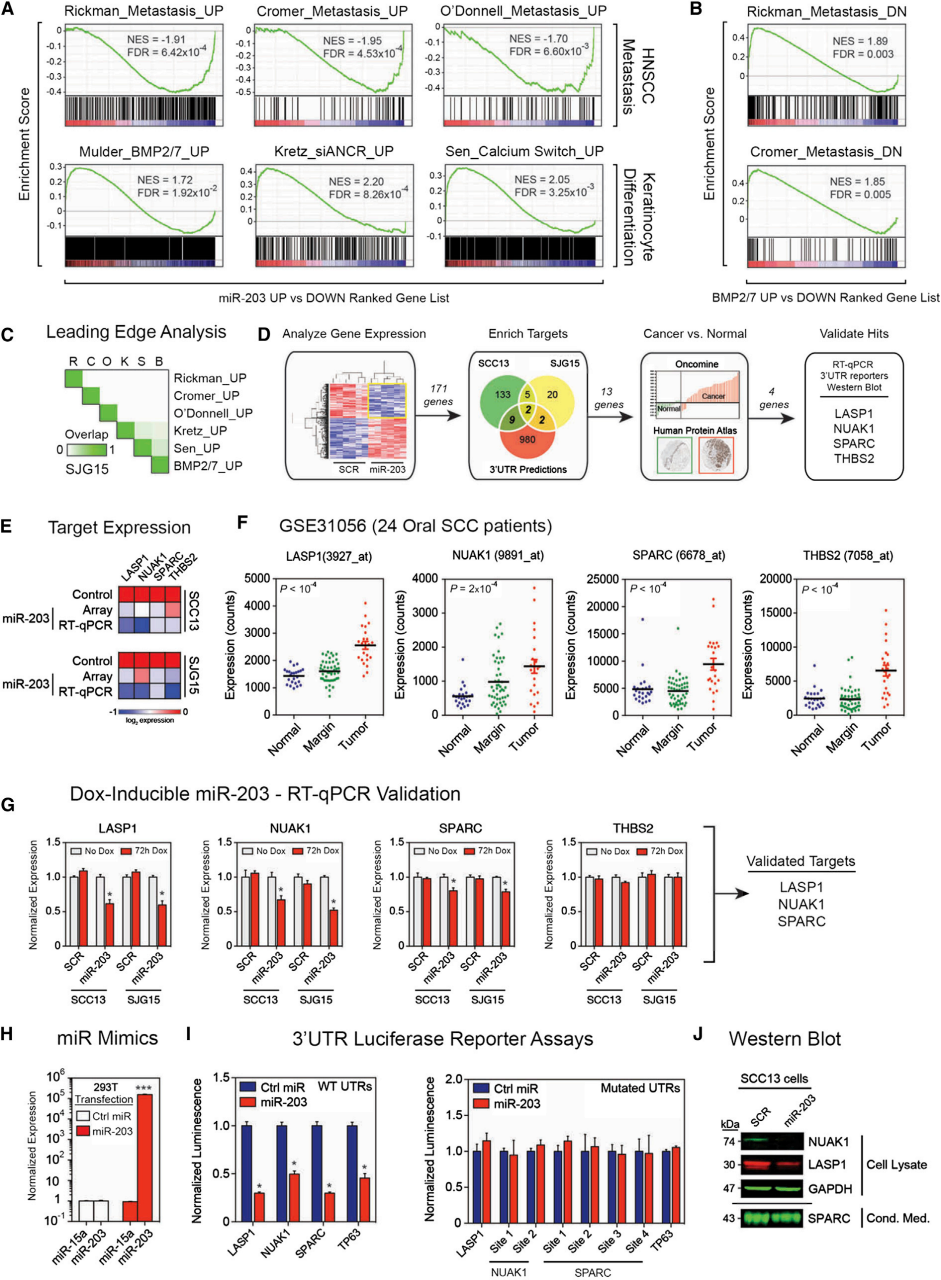
LASP1, SPARC, and NUAK1 Are Direct Target Genes of miR-203 (A) GSEA plots showing negative enrichment of three signatures of genes upregulated in metastatic HNSCC (top row), and positive enrichment with three signatures of genes upregulated during primary human keratinocyte differentiation in vitro (bottom row), in miR-203-expressing SJG15 cells compared with control. NES, normalized enrichment score; FDR, false-discovery rate q value. (B) GSEA as in (A) using the ranked gene list (top upregulated to most downregulated) of genes in BMP2/7-treated keratinocytes compared with control. (C) Leading-edge analysis of genes driving enrichment of metastasis and differentiation signatures in miR-203-expressing SJG15. Signatures on the vertical are abbreviated to first letters only on the horizontal axis. The overlap score (0–1) indicates the degree to which leading-edge genes in each signature are shared with other signatures. (D) Experimental and bioinformatics strategy to identify clinically relevant miR-203 target genes. Microarray expression profiling of stable miR-203 and control expressing SCC13 and SJG15 cells was followed by enrichment for downregulated direct targets using publically available miRNA target prediction algorithms, and subsequently analysis of microarray and protein expression data sets in Oncomine and the Human Protein Atlas to obtain four candidate target genes for validation. (E) Heatmap depicting log_2_ transformed expression levels of four candidate miR-203 target genes in the microarray experiments, validated by qRT-PCR in control and miR-203-expressing SCC13 and SJG15 cells. (F) Analysis of GSE31056 for LASP1, NUAK1, SPARC, and THBS2 expression (counts) in normal tissue (24 samples), oral SCC margins (49 samples), and oral SCCs (23 samples); p values were calculated between normal and tumors using the nonparametric Mann-Whitney test. (G) qRT-PCR for LASP1, NUAK1, SPARC, and THBS2 mRNA expression in SCC13 and SJG15 cells expressing inducible miR-203 or control vector. Cells were either not induced or induced for 3 consecutive days in vitro. Expression normalized to GAPDH. Data are means ± SEM. (H) qRT-PCR expression of miR-15a and mi-20 in 293T cells transfected with 20 nM negative control miRNA (Ctrl miR) or miR-203 for luciferase reporter assays. Expression was normalized to Ctrl miR. Data are means ± SEM. (I) Luciferase reporter assays measuring the ability of transfected miR-203 to repress wild-type (left) and mutant (right) 3′ UTR sequences of LASP1, NUAK1, SPARC, and TP63 (positive control). Measurements are the ratios of firefly/renilla luminescence readings relative to control miRNA transfectants. Data are means ± SEM. (J) Li-Cor western blots of whole-cell lysates (NUAK1, LASP1, and GAPDH) and conditioned medium (SPARC) from control (SCR) and miR-203-expressing SCC13 cell cultures. ^∗^p < 0.05, ^∗∗^p < 0.01, ^∗∗∗^p < 0.001 calculated using unpaired Student’s t test. See also Figures S5 and S6.

To probe the mechanisms by which miR-203 regulates metastasis, we performed genome-wide expression profiling and gene set enrichment analysis (GSEA) of miR-203-expressing SCC13 and SJG15 cells versus scrambled controls. Genome-wide expression changes elicited by miR-203 were negatively enriched for signatures of metastasis in primary human HNSCC arising in various anatomical locations within the aerodigestive tract (Cromer et al., 2004; O’Donnell et al., 2005; Rickman et al., 2008; Figure 5A). A similar negative enrichment between miR-203 and metastasis signatures was also found in melanoma, breast, prostate, and endometrial tumors, all of which have a tendency to metastasize (data not shown). Together, these results suggest that miR-203 enforces a transcriptional landscape in human HNSCC cells that is restrictive for metastasis.

GSEA also revealed that signatures of human keratinocyte differentiation were positively enriched in miR-203-expressing HNSCC cells (Kretz et al., 2012; Mulder et al., 2012; Sen et al., 2010; Figures 5A and 5B). However, there was no overlap between the core genes driving the metastasis and differentiation signatures (Figure 5C). This suggests that miR-203 uses mutually exclusive genetic strategies to prime the transcriptional landscape of HNSCC cells toward a poorly metastatic cell state and a differentiated state. In support of this prediction, there was no difference between control and miR-203-expressing SCC13 tongue tumors and lung metastases in expression of markers of undifferentiated keratinocytes, including keratin-5, keratin-14, α6-integrin, and p63. There was no difference in E-cadherin expression, and all tumors were negative for the epithelial-mesenchymal transition (EMT) markers N-cadherin and vimentin, with the exception of a few cells at the invasive edge (Figures S5A and S5B; cf. Taube et al., 2013). There was also no difference in expression of the keratinocyte differentiation markers involucrin, keratin 10, and loricrin (Figures S5C and S5D), or in markers of proliferation (Ki67 and phospho-histone H3), apoptosis (cleaved-caspase 3 positive), or vascular density (Figures S6A–S6C).

Hence, although miR-203 primed the transcriptional landscape of HNSCC cells toward a differentiated state, primary tumors and lung metastases did not exhibit alterations in differentiation status in vivo. These results suggest that the mechanism by which miR-203 controls metastasis is distinct from its prodifferentiation function in normal epidermis (Yi et al., 2008).

### miR-203 Directly Targets a Cohort of Genes Upregulated in HNSCC

To identify prometastatic miR-203 target genes, we used an integrated genomics, bioinformatics, and experimental approach (Figure 5D). We curated our microarray expression data for genes significantly downregulated by miR-203 and overlapped these hits (171 genes) with a list of 993 miR-203 predicted target genes from 11 publically available algorithms. To enrich for relevance in human HNSCC, we filtered the 13 hits through Oncomine (Rhodes et al., 2007) and the Human Protein Atlas (Uhlen et al., 2010), and discovered four genes (*LASP1*, *NUAK1*, *SPARC*, and *THBS2*) that were upregulated in HNSCC specimens relative to normal mucosa. Furthermore, we observed tumor-specific upregulation of *LASP1*, *NUAK1*, *SPARC*, and *THBS2* in an independent cohort of 24 oral SCC patients not contained in Oncomine (Figure 5F; Rhodes et al., 2007). Genes associated with the actions of miR-203 in other cancers, such as BIRC5 (Saini et al., 2011), SNAI2 (Ding et al., 2013), P63 (Yi et al., 2008), and c-Jun (Sonkoly et al., 2012), were not enriched in our samples.

To validate the results of our analysis, we showed that overexpression of miR-203 led to downregulation of LASP1, NUAK1, SPARC, and THBS2 in SCC13 and SJG15 cells (Figure 5E). Dox induction of miR-203 in SCC13 and SJG15 cells led to downregulation of LASP1, NUAK1, and SPARC, but not THBS2 (Figure 5G).

We focused on LASP1, NUAK1, and SPARC because of their involvement in metastasis-relevant processes such as cytoskeletal dynamics, energy metabolism, and extracellular matrix remodeling, respectively, and their previously described roles in cancer (Liu et al., 2012; Minn et al., 2005; Traenka et al., 2010). To determine whether or not *LASP1*, *NUAK1*, and *SPARC* are direct miR-203 targets, we cloned the 3′ UTRs into a luciferase reporter construct. The 3′ UTR of *TP63*, a known downstream target of miR-203 (Yi et al., 2008), served as a positive control. Transfection of exogenous miR-203 molecules (mimics) into 293T cells led to specific upregulation of miR-203 levels (Figure 5H). miR-203 mimics repressed the *LASP1*, *NUAK1*, *SPARC*, and *TP63* 3′ UTR reporters, while mutation of the various miR-203 binding sites blocked miR-203-mediated regulation of each 3′ UTR (Figure 5I). Western blotting showed a miR-203-dependent reduction in LASP1, NUAK1, and SPARC protein levels (Figure 5J). Moreover, we found significant negative correlations between RNA sequencing (RNAseq) read counts of miR-203 and each of its three direct targets, but not *TP63*, across 225 HNSCC patients in TCGA (Figure S2A). We thus confirmed LASP1, NUAK1, and SPARC as direct target genes of miR-203 in human HNSCC.

### LASP1, NUAK1, and SPARC Are Functionally Important Prometastatic Downstream Effectors of miR-203 that Are Prognostic of Overall Survival in HNSCC

**Figure 6:**
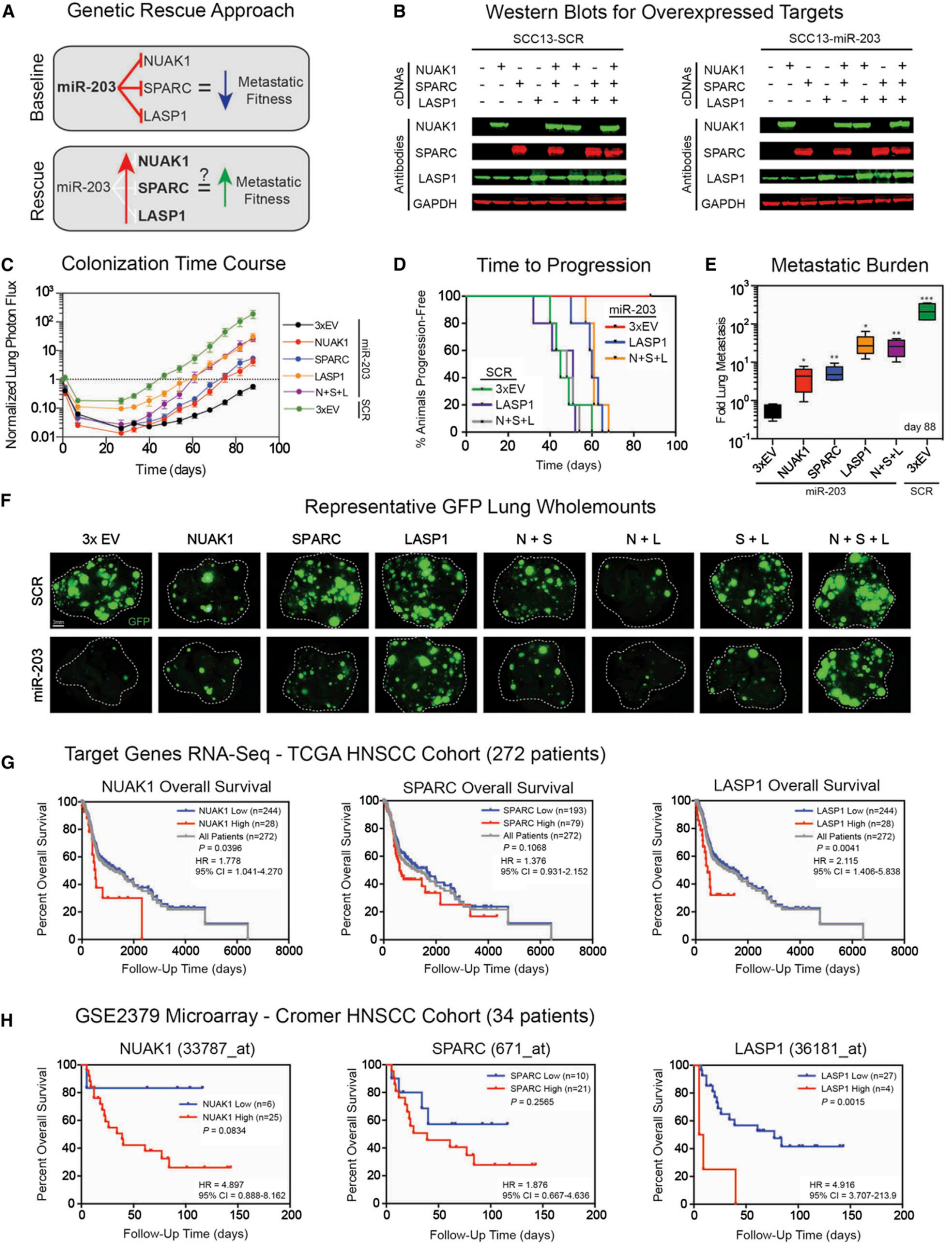
LASP1, SPARC, and NUAK1 Can Overcome miR-203 Metastasis Suppression (A) Schematic model for miR-203-driven suppression of lung metastatic fitness in SCC, and hypothesis for combinatorial genetic rescue by overexpression of miR-203-insensitive LASP1, NUAK1, and SPARC in miR-203-expressing SCC13 cells. (B) Li-Cor western blots of 16 different whole-cell lysates from control (SCR) and miR-203-expressing SCC13 cells also overexpressing single, double, or triple combinations of LASP1, NUAK1, and SPARC cDNAs. GAPDH was used as a loading control. (C) Time course of experimental lung metastasis generated by control (SCR) SCC13 cells expressing 3xEV and miR-203 SCC13 cells expressing 3xEV, NUAK1, SPARC, or LASP1, or NUAK1, SPARC, and LASP1 together (N+S+L) over 88 days (n = 5 per group). Log_10_ y axis; data are means ± SEM with n = 4–5 per group. Data were selected from the full set of combinations presented in Figure S6D. (D) Kaplan-Meier plots for time to progression of lung metastasis of control and miR-203 SCC13 cells expressing 3xEV, LASP1 alone, or all three target gene cDNAs; p values were calculated using the log rank Mantel-Cox test; n = 4–5 per group. (E) Lung metastasis formed by 3xEV, NUAK1, SPARC, or LASP1, or NUAK1, SPARC, and LASP1 together (N+S+L) of miR203 SCC13 cell lines and 3xEV in the SCR SCC13 cell line, analyzed at day 88. Whiskers indicate min/max and the horizontal bar is median, with n = 4–5 per group. Log_10_ y axis. ^∗^p < 0.05 and ^∗∗^p < 0.01 were calculated using the nonparametric Mann-Whitney test in comparison with miR-203 3xEV. (F) Representative whole-mount ex vivo GFP fluorescence microscopy images of lung metastases formed by all 16 combinations of SCC13 cell lines at day 88. Scale bar represents 3 mm. (G) Kaplan-Meier plots for overall survival of 272 HNSCC patients from the TCGA cohort partitioned on the basis of LASP1, NUAK1, and SPARC RNAseq read counts; p values were calculated using the log rank Mantel-Cox test. (H) LASP1, NUAK1, and SPARC mRNA expression was used to partition the GSE2379 cohort of 34 HNSCC patients; p values were calculated using the log rank Mantel-Cox test.

To investigate the functional contribution(s) of LASP1, NUAK1, and SPARC to miR-203-induced inhibition of metastasis, we conducted genetic rescue experiments in vivo (Figure 6A). We engineered control and miR-203 expressing SCC13 cells to stably express all 16 possible combinations of miRNA-insensitive cDNAs (lacking 3′ UTRs) encoding *LASP1*, *NUAK1*, and *SPARC*, and validated correct target gene reconstitution by western blotting (Figure 6B). The levels of exogenous protein expressed were sufficiently high that endogenous NUAK1 and SPARC (Figure 5J) could not be seen on the blots (Figure 6B).

All 16 SCC13 populations were intravenously injected into mice and lung metastasis was monitored for up to 88 days (Figures 6C, S6D, and S6E). Consistent with our earlier findings (Figure 2), SCC13 cells expressing miR-203 together with three empty-vector controls (3xEV) did not colonize the lungs, whereas 3xEV SCC13 SCR cells generated numerous macrometastatic lesions (Figure 6C). Overexpression of SPARC or NUAK1 alone led to a small increase in metastasis of miR-203 cells, whereas overexpression of LASP1 alone caused a pronounced stimulation, which was comparable to overexpressing all three proteins (Figures 6C, S6E, and S6E). Overexpression of LASP1 and/or SPARC, or combined expression of LASP1, SPARC, and NUAK1 did not enhance metastasis of SCR SCC13 cells (Figures 6C–6F, S6D, and S6E). Unexpectedly, when NUAK1 was expressed alone or in combination with SPARC or LASP1, SCR SCC13 cells produced fewer metastases (Figures 6F and S6E).

Individual or combined reintroduction of NUAK1, SPARC, and/or LASP1 was sufficient to bolster the lung metastatic colonization by SCC13 expressing miR-203 (Figure 6C–6F). NUAK1, SPARC, and LASP1 promoted exit from dormancy (Figure 6D) and overt pulmonary colonization, increasing the lung metastatic burden by 7-fold, 10-fold, and 53-fold, respectively (Figures 6E and 6F). Thus, each miR-203 target promotes metastasis in the presence of miR-203, with the effect of LASP1 being most pronounced. In addition, knockdown of LASP1 reduced the clonogenic potential of SCC13 cells in vitro (Figures S6F and S6G), recreating the phenotype observed when miR-203 is expressed in the SCC13 cell line (Figure 2B).

The clinical relevance of the miR-203 target genes was confirmed in patient data. High expression of *LASP1*, *NUAK1*, and *SPARC* correlated with poor prognosis in both the TCGA cohort and a separate publically available HNSCC cohort (Figures 6G and 6H; Cromer et al., 2004).

We conclude that miR-203 inhibits lung metastasis, not by triggering differentiation, but by directly targeting the prometastatic genes *LASP1*, *SPARC*, and *NUAK1*, which are prognostic factors in human HNSCC.

## Discussion

Our studies uncover miR-203 as a potent suppressor of key postextravasation events during lung metastasis. Reintroducing miR-203 into already established pulmonary nodules elicits their regression, suggesting the potential for therapeutic modalities aimed at activating miR-203 in cancer cells to treat metastatic HNSCC.

Recent evidence suggests that cancer cells hijack normal stem cell self-renewal signaling pathways to generate and propagate tumors in vivo (Reis et al., 2011*)*. If so, coaxing cancer cells to differentiate may inhibit tumor growth and metastasis. Although miR-203 promotes differentiation of normal epidermal stem cells (Jackson et al., 2013; Yi et al., 2008), our study shows that it does not promote carcinoma differentiation in vivo. Instead, miR-203 controls HNSCC metastasis by targeting a network of prometastatic proteins, including LASP1, SPARC, and NUAK1. Thus, we propose that miR-203 undergoes a context-dependent functional switch from regulating normal differentiation to acting as a differentiation-independent roadblock to metastasis.

It is known that the genetic and epigenetic alterations sustained by host cells during malignant transformation alter the repertoire of mRNA species available for targeting by miRNAs (Lujambio and Lowe, 2012). Thus, miRNAs can perform cellular-context-dependent functions driven by different cohorts of downstream effectors. For example, miR-126 inhibits breast cancer metastasis either by modulating the primary tumor microenvironment (Zhang et al., 2013) or by inhibiting lung metastatic colonization (Png et al., 2012) in mouse or human breast cancer models, respectively. As such, the rewiring of miR-203 function from regulating differentiation to inhibiting metastasis is likely to be explained by changes in target gene selection from differentiation-relevant proteins, such as TP63, to metastasis-promoting factors, such as LASP1, SPARC, and NUAK1.

Our mechanistic studies identify LASP1, SPARC, and NUAK1 as important direct downstream effectors for miR-203-mediated inhibition of HNSCC metastasis. Prior studies have shown that these proteins promote cancer cell migration and invasion in vitro, promote tumor progression and/or metastasis in vivo, and correlate with poor overall survival in diverse carcinomas (Chang et al., 2012; Chin et al., 2005; Davis et al., 2008; Liu et al., 2012; Minn et al., 2005; Suzuki et al., 2004; Traenka et al., 2010; Viticchiè et al., 2011). We speculate that LASP1, NUAK1, and SPARC promote postextravasation survival and/or engraftment, shorten dormancy, and bolster overt pulmonary colonization by HNSCC cells.

Our data indicate that miR-203 is an upstream governor of several distinct cellular pathways that converge to enforce a poorly metastatic cell state, and highlight the importance of cellular context in determining the effects of a specific miRNA on metastasis. Indeed, miR-203 regulates tumor progression in breast and prostate cancer, but the targets of its action are very distinct among tissue types. In advanced metastatic prostate cancer, miR-203 targets BIRC5 (Saini et al., 2011), whereas in breast cancer metastasis it targets SNAI2 (Ding et al., 2013). This suggests that miR-203 not only functionally switches between tissue homeostasis and cancer progression, as shown here, but also switches targets depending on the cancer type.

In conclusion, our studies highlight miR-203 and its effectors as promising routes for therapeutic intervention in metastatic HNSCC. Unlike most anticancer agents and antimetastatic strategies currently in clinical trials (Valastyan and Weinberg, 2011), miR-203 can antagonize metastasis even after cancer cells have seeded the lung and formed clinically advanced nodules. In light of the development of targeted strategies to deliver nucleic acids into tumor cells (Davis et al., 2008), we envision the potential value of using miR-203 mimetics to alleviate otherwise therapeutically intractable metastatic HNSCC.

## Experimental Procedures

### Cell Lines

SCC13, SCC25, SJG cell lines, and normal keratinocytes were cultured in FAD medium under standard conditions. Work with human material was either carried out in compliance with the UK Human Tissue Act (2004) and approved by the National Research Ethics Service (08/H0306/30) or according to the recommendations of the local ethics committee and the German Medical Council for diagnostic tissue used in research.

### Animal Studies

Tongue xenografting and experimental lung metastasis experiments were performed as previously described (Goldie et al., 2012). Animal studies were subject to Cancer Research UK and King’s College London ethical review and performed in accordance with an approved UK Government Home Office license. A dox-rich diet (Harlan) was used to induce miR-203 expression in vivo. Bioluminescent imaging was conducted using a Xenogen IVIS 200 system (Perkin Elmer).

### qRT-PCR, Microarrays, and GEO Data Sets

RNA was isolated using a miRNeasy Mini Kit (QIAGEN), and qRT-PCR was performed using TaqMan probes (Life Technologies) and SYBR Green primers. Genome-wide expression analysis was carried out on Illumina Human HT12 version 4 arrays. Microarray data sets GSE31056 and GSE2379 were downloaded from NCBI GEO. TCGA data were downloaded from https://tcga-data.nci.nih.gov/tcga/ and http://www.broadinstitute.org/tcga/home.

### Statistical Analysis

An unpaired two-tailed Student’s t test (in vitro experiments) or a nonparametric Mann-Whitney test (in vivo mouse experiments) was used for comparisons, with p < 0.05 considered significant. A log rank (Mantel-Cox) test was used to compare survival curves and compute hazard ratios, and Fisher’s exact test used to compare human cohorts on the basis of clinical characteristics.

## Author Contributions

N.B. and F.M.W. conceived the project. N.B., S.W., and F.M.W. wrote the manuscript. N.B., S.W., and A.M. performed experiments. S.J.G. and S.R.Q. provided reagents.

## Accession Numbers

The NCBI Gene Expression Omnibus accession number for the gene expression data reported in this paper is GSE47028.

## Supplemental Information

Document S1. Supplemental Experimental Procedures, Figures S1–S6, and Table S1

Document S2. Article plus Supplemental Information
