# Supplementary material for: Rewiring of an Epithelial Differentiation Factor, miR-203, to Inhibit Human Squamous Cell Carcinoma Metastasis
Source: Cell Rep. 2014 Oct 2;9(1):104–17. doi: 10.1016/j.celrep.2014.08.062 (PMC4536294; doi:10.1016/j.celrep.2014.08.062)
Supplement: Document S1. Supplemental Experimental Procedures, Figures S1–S6, and Table S1 [file mmc1.pdf]

Cell Reports, Volume *9*

Supplemental Information

Rewiring of an Epithelial Differentiation Factor, miR-203, to Inhibit Human Squamous Cell Carcinoma Metastasis

Nathan Benaich, Samuel Woodhouse, Stephen J. Goldie, Ajay Mishra, Sven R. Quist, and Fiona M. Watt

SUPPLEMENTAL INFORMATION

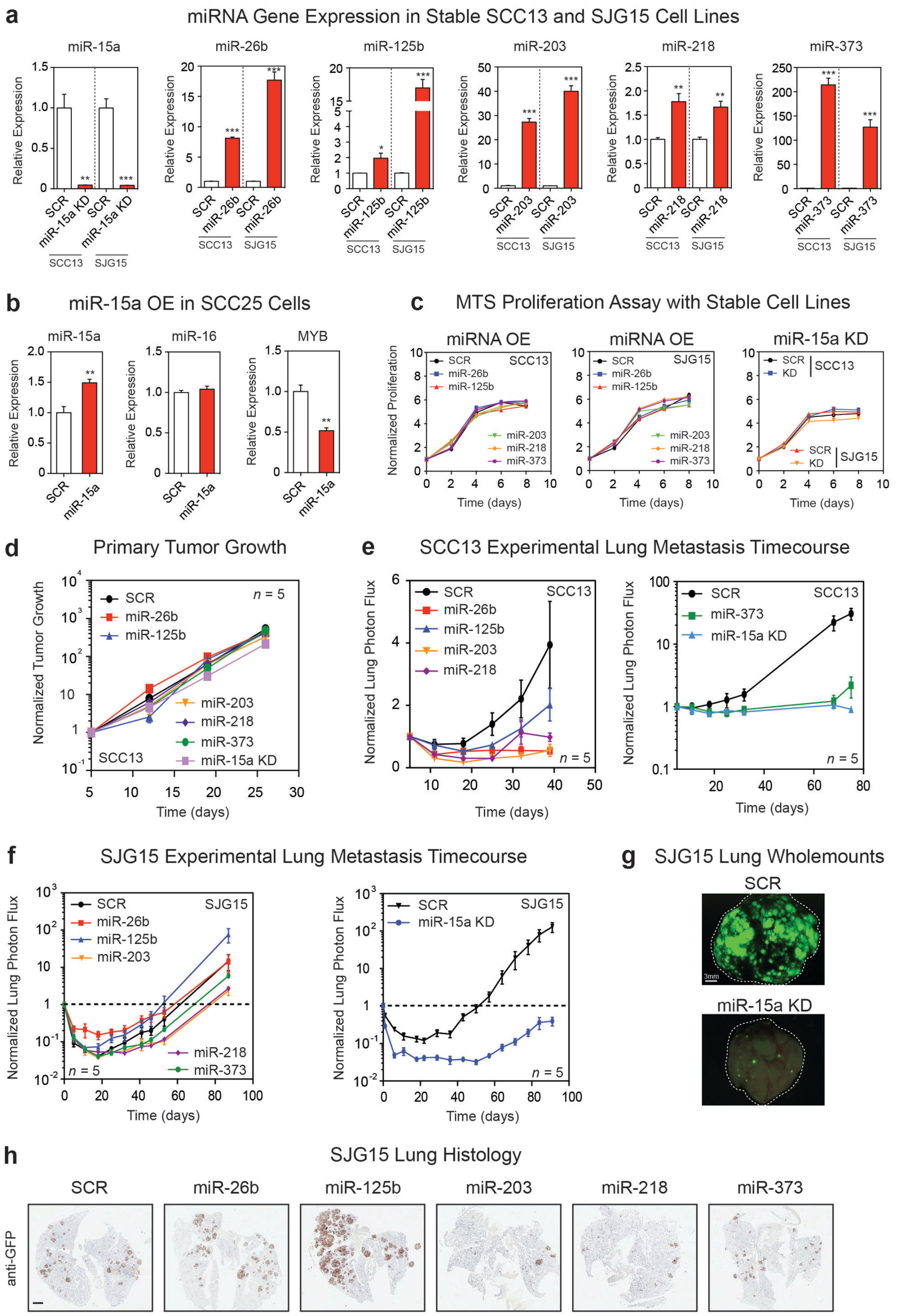

Figure S1. Stable SCC13 and SJG15 cell line validation and additional *in vivo* data panels. Related to Figure 1

(a) qRT-PCR was used to assay expression levels of mature miR-15a, miR-26b, miR-125b, miR-203, miR-218, and miR-373 in SCC13 and SJG15 cells stably infected with either overexpression or knockdown (KD) vectors. Expression was normalized to SCR in each case. Data are mean ± SEM.

(b) miR-15a overexpression, but not miR-16, in SCC25 cells was verified by qRT-PCR, as well as its published target gene, *MYB*. Data are mean ± SEM.

(c) MTS assay measuring *in vitro* growth kinetics of SCC13 and SJG15 cells from panel (a). Each data point was normalized to the day 0 value within each group of cells. Data are mean ± SEM.

(d) Time course of primary tongue tumor growth starting with 10^5 SCC13 cells. Total lung photon flux was normalized to day 5 for each mouse and expressed as a relative fold change. Log_{10} y-axis, data are mean ± SEM with *n*=5 per group.

(e) Time course of SCC13 experimental lung metastasis using cells in panel (a). Data are normalized to bioluminescence at input and presented as mean ± SEM with *n*=4-5 per group.

(f) Same as (e) except using SJG15 cells.

(g) *Ex vivo* fluorescence lung wholemounts of mice tail-vein injected with control or miR-15a KD SJG15 cells at day 91. Scale bar, 3 mm.

(h) Matched representative anti-GFP immunohistochemistry of lungs injected with SJG15 after 87 days after tail-vein injection of indicated SJG15 cells. Scale bar, 50 µm.

**P* < 0.05; ***P* < 0.01; ****P* < 0.001 calculated using a non-parametric Mann-Whitney test (mouse experiments) or a two-tailed Student's t-test (qRT-PCR data).

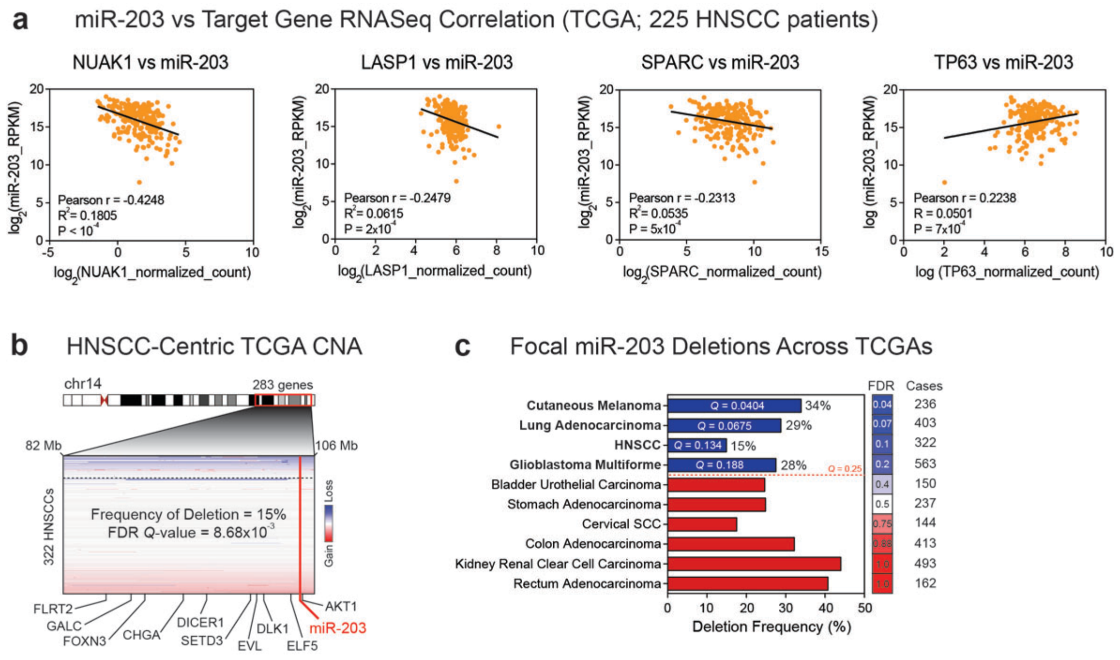

Figure S2. RNASeq correlations between miR-203 and target genes in HNSCC TCGA cohort and CNA analysis. Related to Figure 2

(a) miRNASeq (RPKM) and RNASeq (normalized counts) paired values for 225 HNSCC TCGA primary tumors were plotted against one another and correlations calculated using a Pearson's test. Note negative correlation between miR-203 and NUAK1, LASP1, and SPARC, but absence of such correlation with a previously reported target, TP63.

(b) Schematic representation of a 24 Mb region on human chromosome 14 incorporating miR-203 that is significantly focally deleted in 15% of HNSCC cases in the 322 TCGA patient cohort. Indicated genes are an example subset. Blue indicates deletion and red indicates amplification. False discovery rate Q-value (<0.25 cutoff) calculated using GISTIC statistics.

(c) A miR-203-centric GISTIC analysis of TCGAs cohorts reveals significant deletion of miR-203 in multiple tumor types (significant in red; non-significant in blue, using *Q*<0.25 as a cutoff).

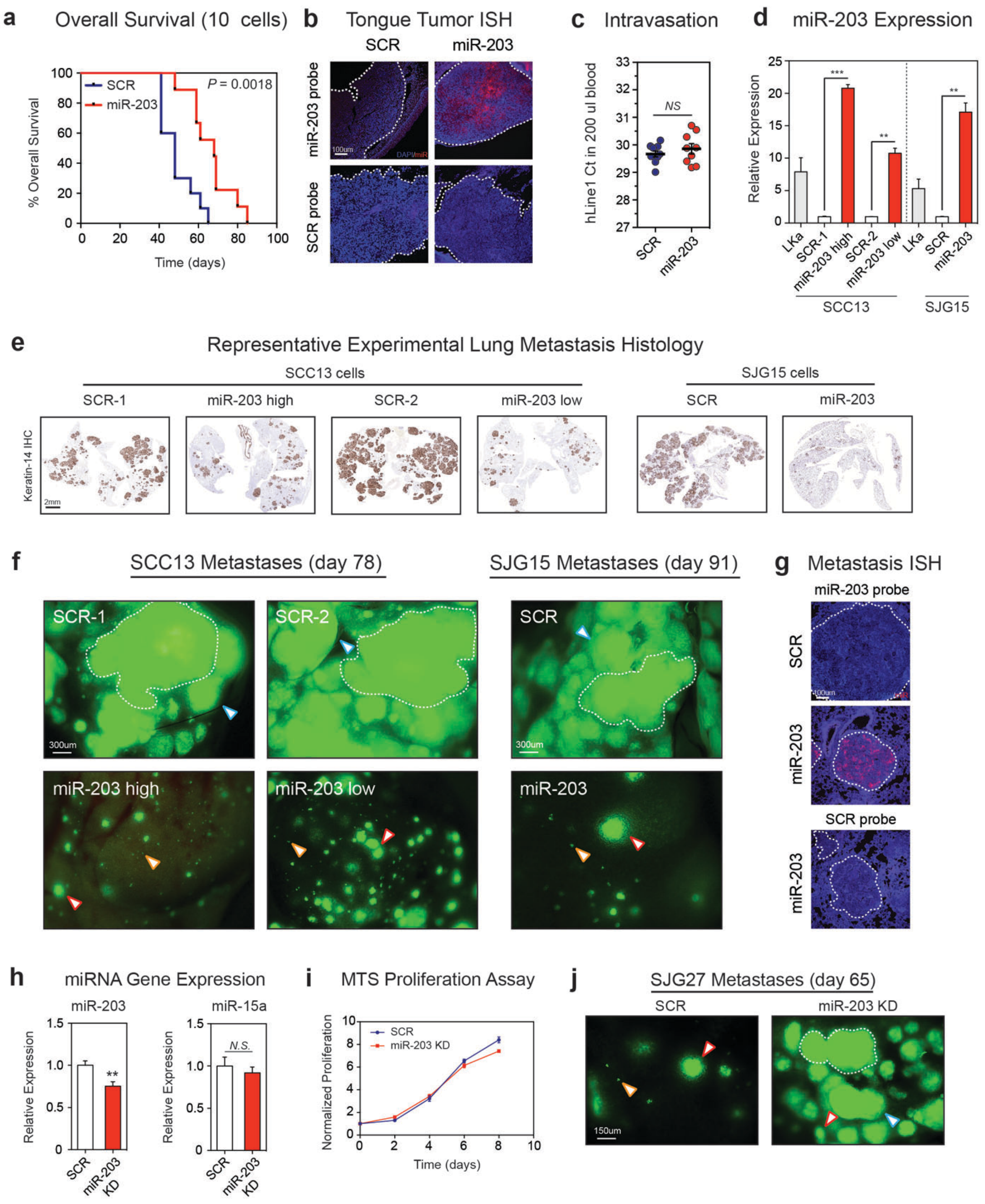

Figure S3. miR-203 primarily regulates lung metastatic colonization *in vivo*. Related to Figure 2 and Figure 3

(a) Kaplan-Meier overall survival curve for animals tongue xenografted with 10^4 control or miR-203 expressing SCC13 cells (*n*=10 per group).

(b) Primary tongue tumors formed by control (SCR) and miR-203 expressing SCC13 cells stained with miR-203 or scrambled LNA probes (Exiqon). miR-203 signal in red and DAPI nuclei in blue. Scale bar is 100 µm. Dotted lines outline metastases.

(c) *in vivo* tumor cell intravasation was measured by qRT-PCR for human-specific *AluJ* repeat elements present in circulating tumor cells within 200 ul of whole mouse

blood. *n*=10 (SCR) and *n*=9 (miR-203). *P*-values calculated using a non-parametric Mann-Whitney test.

(d) qRT-PCR expression of miR-203 high and miR-203 low SCC13 cells, SJG15 cells expressing miR-203, and normal human primary lip keratinocytes (LKa). Expression was normalized to SCR in each case. Data are mean ± SEM.

(e) Representative keratin-14 immunohistochemistry of experimental lung metastases formed by SCC13 cells expressing high or low miR-203, as well as primary SJG15 cells expressing miR-203. Sections are of matched lungs from fluorescence images in Figure 2j. Scale bar is 2mm.

(f) High magnification GFP fluorescence microscopy images of control and miR-203 experimental lung metastases formed by SCC13 or SJG15 cells *in vivo* after 78 or 91 days, respectively. Dashed lines outline massive metastatic areas observed only in control animals. Blue arrowheads point to macrometastases; red arrowheads indicate small macrometastases or micrometastases; orange arrowheads show clusters of single cells. Scale bars are 300 μm.

(g) Experimental lung metastases formed by control (SCR) and miR-203 expressing SCC13 cells were stained with miR-203 or scrambled LNA probes (Exiqon). Note presence of miR-203 signal in metastases formed by miR-203 expressing cells, but not control cells. Scale bar and dotted lines are the same as in panel (e).

(h) qRT-PCR gene expression measuring miR-203 and miR-15a (control) levels in SJG27 cells expressing scrambled control hairpin (SCR) or miR-203 knockdown vector (KD). Expression was normalized to SCR in each case. Data are mean ± SEM.

(i) MTS assay measuring *in vitro* growth kinetics of control and miR-203 knockdown SJG27 cells. Each data point was normalized to the day 0 value within each group of cells. Data are mean ± SEM. *P*-values calculated using a two-tailed Student's t-test.

(j) High magnification GFP fluorescence microscopy images of SJG27 control and miR-203 KD experimental lung metastases after 65 days. Dashed lines outline massive metastatic areas observed only in control animals. Blue arrowheads point to macrometastases; red arrowheads indicate small macrometastases or micrometastases; orange arrowheads show clusters of single cells. Scale bars are 150 μm.

**P* < 0.05; ***P* < 0.01; ****P* < 0.001 calculated using a two-tailed Student's t-test for panels (d) and (h).

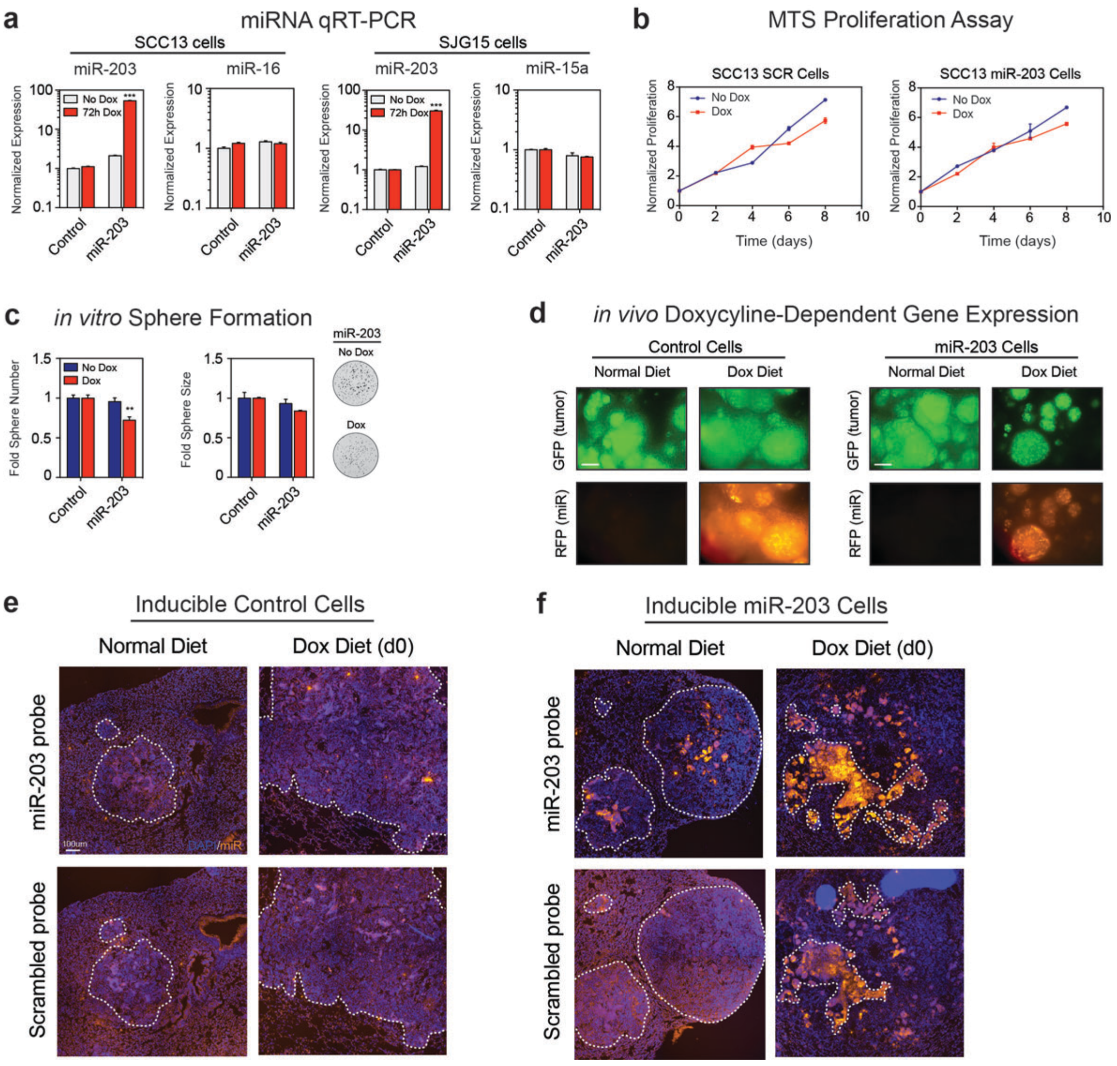

Figure S4. Validation experiments for doxycycline-inducible miR-203 vector system. Related to Figure 4

(a) qRT-PCR expression analysis of miR-203 and either miR-16 or miR-15a (control miRNAs) in control and miR-203 inducible SCC13 and SJG15 cells after *in vitro* treatment with dox for 72h. Note dox-dependent induction of miR-203 but not irrelevant miRNAs, miR-15a or miR-16. Data are mean ± SEM with a $\log_{10}$ y-axis

(b) MTS assay measuring *in vitro* growth kinetics of SCC13 in panel (b) either non-induced or induced with dox starting 3 days prior to the experiment. Each data point was normalized to the day 0 value within each group of cells. Data are mean ± SEM.

(c) *in vitro* anchorage-independent soft agar assay using SCR and miR-203 inducible SCC13 cells either not induced or induced continuously with dox. Fold changes in sphere number and size (5000 cells seeded/well) are normalized to respective non-dox

treated groups. Data are presented mean ± SEM. Representative GFP scans of wells with non-induced or induced miR-203 spheres are shown on the right.

(d) *ex vivo* GFP (all tumor cells) and RFP (only induced SCR or miR-203) fluorescence microscopy images of lung metastases formed by control and miR-203 inducible SCC13 cells in animals fed with either a normal diet (no dox) or dox-rich diet starting at 10 days post-injection. Images are at 8x magnification, scale bar is 500 μm

(e) and (f) Experimental lung metastases formed by inducible control (SCR) (e) and miR-203 expressing SCC13 cells (f) in mice fed with normal diet (non-induced) or dox-rich diet starting at day 0 of the experiment were stained with miR-203 or scrambled LNA probes (Exiqon). miR-203 signal is orange and DAPI nuclei in blue. Scale bar is 100 μm and lung metastases outlined using dotted lines. Note the absence of miR-203 signal in non-induced and induced control metastases in panel (e), and the strong miR-203 signal present in dox-induced miR-203 lung metastases in panel (f).

*$P < 0.05$; **$P < 0.01$; ***$P < 0.001$ calculated using a two-tailed Student's t-test.

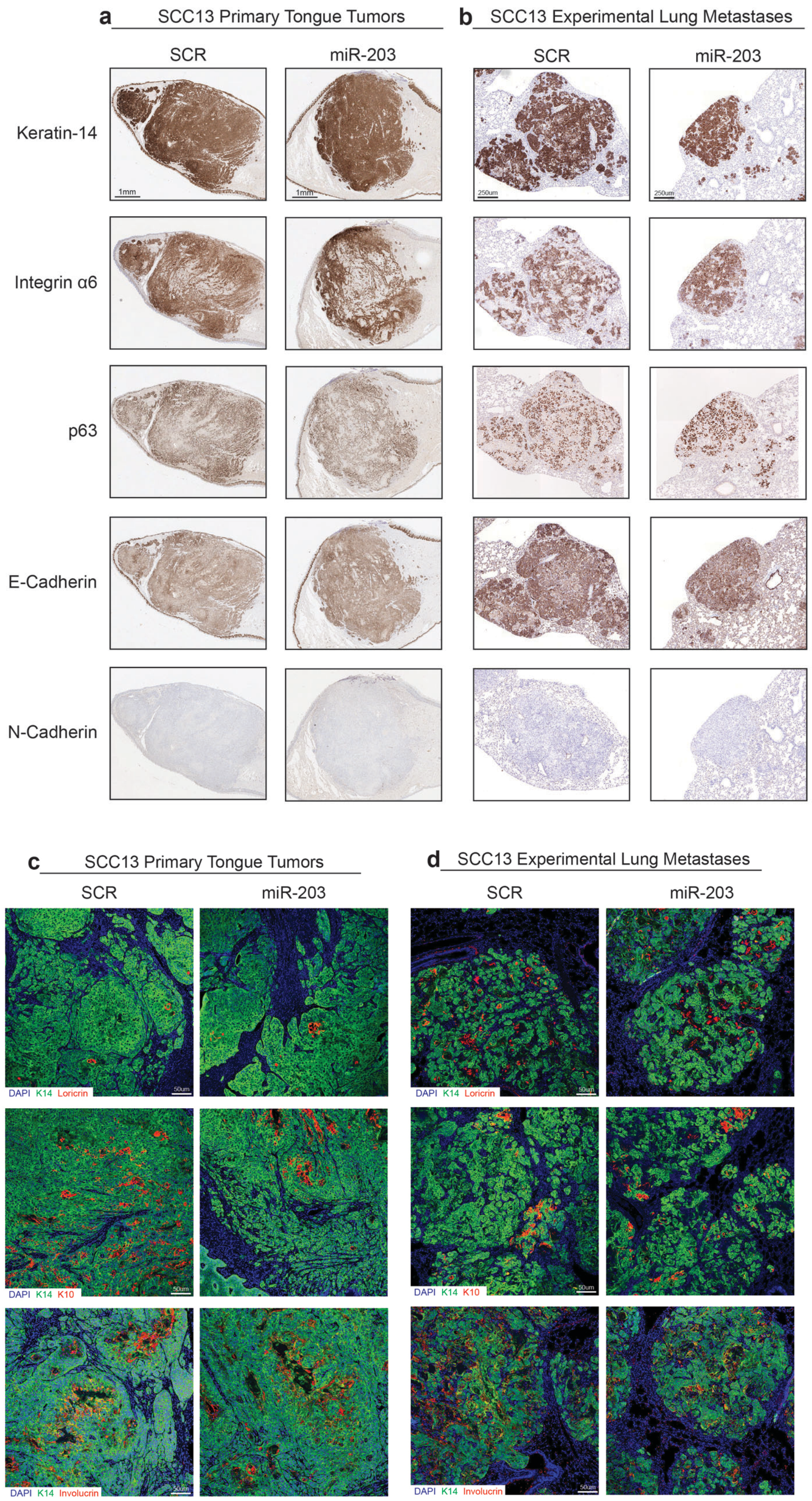

Figure S5. Analysis of epithelial markers and differentiation status in SCC13 primary tongue tumors and experimental lung metastases. Related to Figure 5.

(a-d) Primary tongue xenograft tumors (a, c) and experimental lung metastases (b, d) generated by SCC13 cells expressing control (SCR) or miR-203 were immunolabelled for: (a, b) basal keratinocyte markers Keratin-14, Integrin α6, p63, the epithelial marker E-cadherin, and the mesenchymal marker N-cadherin; (c, d) Keratin-14 (green), and the keratinocyte terminal differentiation markers Loricrin, Keratin-10 and Involucrin (red) with DAPI nuclear counter-stain (blue). (a, b) Note persistence of basal keratinocyte marker expression in both control and miR-203 tumors and metastases, as well as maintenance of an epithelial phenotype. (c, d) Note presence of terminally differentiated cells in both control and miR-203 tumors and metastases. Scale bars: (a, b) primary tumors 1 mm, lung metastases 250 μm; (c, d) 50 μm.

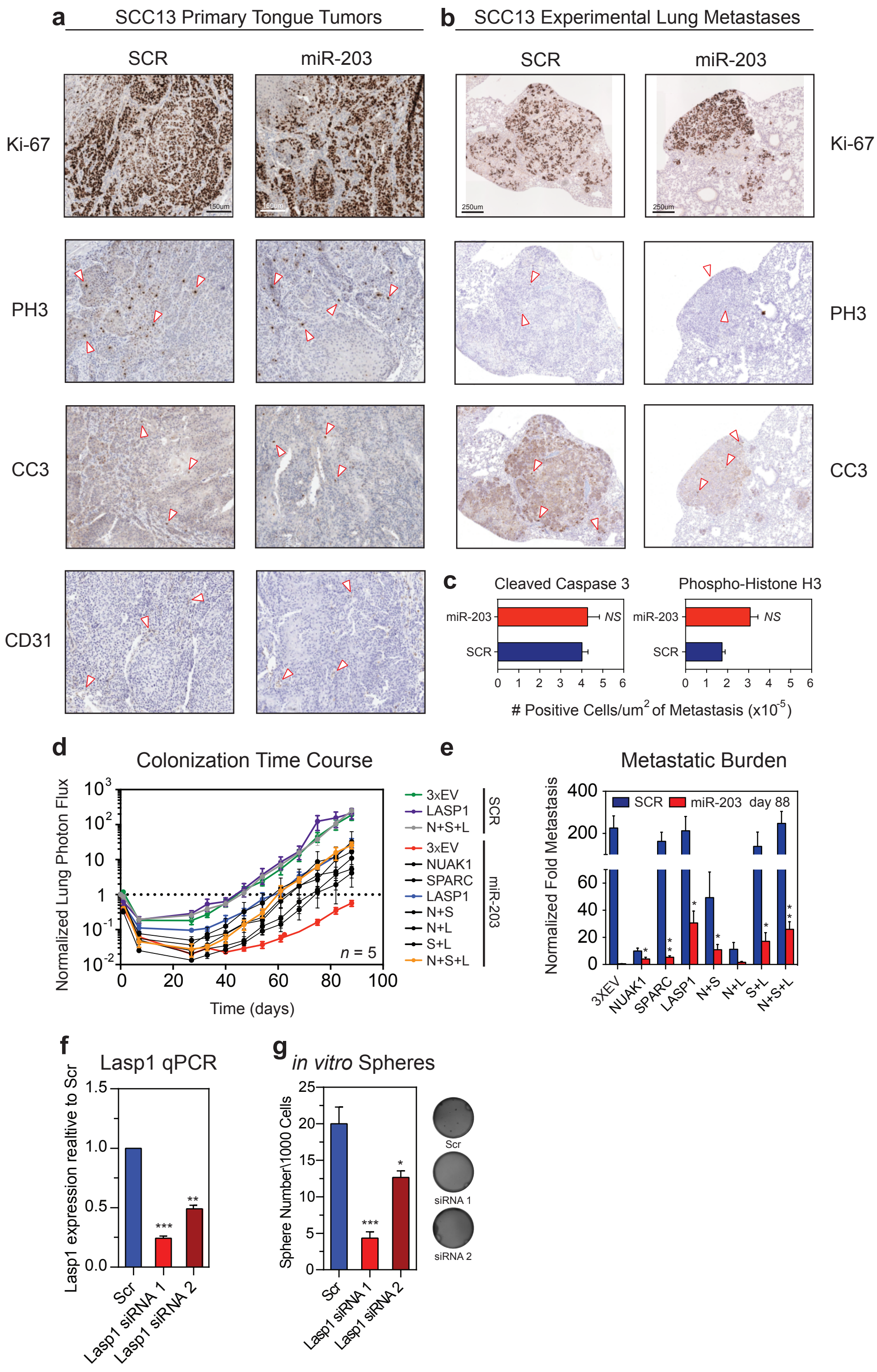

Figure S6. Immunohistochemical analysis of proliferation, apoptosis, and vascularity in SCC13 primary tongue tumors and experimental lung metastases and validation of role of LASP1 in anchorage independent growth of SCC13 cells. Related to Figure 5.

(a) and (b) Primary tongue xenograft tumors (a) and experimental lung metastases (b)

generated by SCC13 cells expressing control (SCR) or miR-203 were immunohistochemically stained for markers of proliferation (Ki-67 and PH3), apoptosis (CC3), and vascularity (CD31). Scale bars are 150 μm (tumors) and 250 μm (metastases).

(c) Quantification of CC3- and PH3-positive cells in control and miR-203 expressing SCC13 lung metastases. Data are presented as means (average of 15 individual metastases analyzed in one section per mouse, *n*=3 mice per group) ± SEM. A non-parametric Mann-Whitney test was used to calculate significance.

(d) Time course of experimental lung metastasis generated by control (SCR) SCC13 cells expressing 3xEV, LASP1 alone, or NUAK1+SPARC+LASP1 (N+S+L) as representative controls, and miR-203 SCC13 cells expressing all 8 cDNA combinations over 88 days (*n*=5 per group). Log_{10} y-axis, data are mean ± SEM with *n*=4-5 per group.

(e) Lung metastases formed by all 16 combinations of SCC13 cell lines at day 88. Data are presented as means ± SEM, *n*=4-5 per group. $*P < 0.05$ and $**P < 0.01$ calculated using a non-parametric Mann-Whitney test comparing miR-203 3xEV with miR-203 expressing one, two, or all three target gene cDNAs.

(f) qRT-PCR was used to assay for the expression levels of LASP1 in SCC13 cells transfected with either SCR control or two independent, LASP1 siRNAs (1 or 2). Expression was normalised to SCR. Data are mean ± SEM.

(g) *in vitro* sphere formation in an anchorage-independent soft-agar assay. The number of spheres was quantified after 3 weeks. Data are presented as mean ± SEM with representative wells shown.

Patient #	Age at diagnosis	Sample ID	Sample description
1	58	A	Primary skin SCC on left cheek with local skin metastasis. Patient suffered infraorbital metastasis infiltrating bone 26 months after diagnosis.
2	82	A	Primary skin SCC on left lower leg
2		B	Local skin metastasis on left knee in transit to lymph nodes suffered 14 months after diagnosis
2		C	Local skin metastasis on left knee following tumor progression post-chemo
3	78	A	Primary skin SCC on frontal head
3		B	Local skin metastasis in transit to lymph nodes
3		C	Local skin metastasis in transit to lymph nodes
3		D	Local skin metastasis in transit to lymph nodes
3		E	Local skin metastasis in transit to lymph nodes
3		F	Local skin metastasis in transit to lymph nodes
4	69	A	Primary skin SCC on left frontal head
4		B	Local skin metastasis in transit to lymph nodes
4		C	Local skin metastasis in transit to lymph nodes
5	81	A	Primary lower lip SCC
5		B	Primary lower lip SCC with potential right submandibular lymph node metastasis
6	75	A	Primary skin SCC on left second finger. Later detected 2/7 positive left axilla lymph nodes; 2 months later found distant metastasis to lungs and kidneys. Overall survival 24 months.
7	81	A	Primary skin SCC
7		B	Primary skin SCC
7		C	Ulcerated primary skin SCC on left temple involving subcutaneous metastasis in right preauricular lymph node
7		D	Local skin metastasis in transit to lymph nodes
7		E	Local skin metastasis in transit to lymph nodes
8	85	A	Primary skin SCC on right temple
8		B	Local skin metastasis in transit to lymph nodes
8		C	Local skin metastasis in transit to lymph nodes
9	80	A	Primary skin SCC above left front parietal lobe. Patient suffered metastasis to parotid gland and

			cervical lymph node 2 months after local spread.
9		B	Local skin metastasis in transit to lymph nodes
10	86	A	Primary skin SCC on right temple
10		B	Local skin metastasis
10		C	Local skin metastasis
11	90	A	Primary skin SCC on right frontal head
11		B	Local skin metastasis in transit to lymph nodes

Table S1. Clinical information of patients from whom matched skin and SCCs were resected and used for miRNA *in situ* hybridization. Related to Figure 2.

Supplemental Experimental Procedures

Cell Lines and Human Tissue

The human facial skin SCC line, SCC13, was obtained from ATCC. Primary human "SJG" cell lines were established from freshly resected HNSCC specimens of patients treated at Addenbrooke's Hospital, Cambridge. Primary normal human lip (strain LKa) and foreskin keratinocytes were obtained from surgical operations at the same hospital. Work with human material was carried out in compliance with the UK Human Tissue Act (2004) and approved by the National Research Ethics Service (08/H0306/30), or the German Medical Council, according to the recommendations of the local ethics committee, for tissue used in research. Appropriate informed consent was obtained from patients prior to their operation.

All cell lines were cultured at 37°C with 5% CO_2 in FAD medium (three parts DMEM medium, one part F12 medium and 10^{-4} M adenine, supplemented with 10% Fetal Bovine Serum Gold (PAA), 2 mM L-glutamine, 0.5 μg/ml hydrocortisone, 5 μg/ml insulin, 10^{-10} M cholera toxin, 10 ng/ml epidermal growth factor, 100 IU/l penicillin and 100 μg/l streptomycin (Gibco). HEK293T cells for lentiviral packaging were maintained in DMEM supplemented with 10% Fetal Bovine Serum, 100 IU/l penicillin and 100 μg/l streptomycin (Gibco). Cell lines passed mycoplasma testing.

For *in vitro* sphere forming assays, 1 ml of a 1% agar/FAD medium base layer was added to a 6-well culture plate (Corning) and 1,000 – 3,000 cells (depending on assay) were suspended in 1 ml 0.8% agar/FAD medium and seeded on top the solidified base layer. The cells were cultured for 21 days, stained with 0.01% Crystal Violet and imaged using a Bio-Rad XR+. For *in vitro* doxycycline induction

experiments, cells were grown in the presence of 1mg/ml doxycycline (filtered and diluted in PBS) for 3 days (Sigma).

Plasmid Construction

To generate miRNA-expression vectors, the human sequences encoding miR-15a, miR-26b, miR-125b-1, miR-203, miR-218-1 and miR-373, as well as up to 250 bp of upstream and downstream flanking genomic sequence, were PCR-amplified from normal human keratinocyte genomic DNA using Phusion High-Fidelity Polymerase (NEB) with 10% DMSO and buffers for GC-rich sequences. Relevant primer sequences for cloning are available in supplementary information. miRNA PCR products were verified by gel electrophoresis, purified using the QIAquick PCR Purification Kit (Qiagen), subcloned into the *XhoI* and *MluI* sites of the pGIPZ and/or pTRIPZ lentiviral expression vectors (Thermo), and sequence-verified. The pGIPZ and pTRIPZ lentiviral scrambled hairpin control plasmids were purchased from Open Biosystems. To knock down expression of miR-15a and miR-203, miRZip lentiviral expression vectors were purchased from System Biosciences.

Precision LentiORFs (pLOC backbone) encoding human LASP1 (clone ID PLOHS_100004169) and SPARC (clone ID PLOHS_100006045) cDNAs with their native stop codons were purchased from Thermo. An ORFeome Collaboration (pENTR223.1 backbone) encoding human NUAK1 cDNA (clone ID 100064197) with its native stop codon was purchased from Thermo. LeGO-iC2 (SFFV promoter, IRES-mCherry; Addgene plasmid 27345) and LeGO-iCer2 (SFFV promoter, IRES-Cerulean; Addgene plasmid 27346) were rendered Gateway-competent using the Gateway Vector Conversion System with One Shot *ccdB* Survival Cells (Invitrogen). These plasmids were used as empty vector controls. NUAK1 cDNA was then inserted into LeGO-iCer2 and SPARC cDNA into LeGO-iC2 using standard Gateway cloning techniques (Invitrogen) and sequence verified. A pLOC empty vector was generated by excising the LASP1 cDNA, blunting with Klenow, and ligating ends (NEB).

To generate psiCheck2-3'UTR reporter plasmids, relevant 3'UTR sequences were obtained from Ensembl, PCR amplified from normal human keratinocyte genomic DNA, verified by gel electrophoresis and gel purified when required, and subsequently cloned into the *XhoI* and *NotI* sites of psiCheck2 (Promega). Mutant UTRs were generated using a QuikChange XL Site-Directed Mutagenesis kit

(Agilent) with the following differences: extension time (2.5min/kb), 3% DMSO, and 100ng input DNA. All plasmids were sequenced verified.

A retrovirus expressing a yellow fluorescent protein and codon-optimized firefly luciferase (YFP-Luc) fusion protein was a generous gift from Dr. Scott Lyons.

Virus Production and Generation of Stable Cell Lines

pGIPZ, pTRIPZ, miRZip, LeGO, and pLOC lentiviral vectors were packaged using pMDLg/pRRE (Addgene plasmid 12251), pRSV-Rev (Addgene plasmid 12253), and pMD2.G (Addgene plasmid 12259). The YFP-luciferase vector was packaged using the FELIX system of pCMV-VSVg (Addgene pCI-VSVg) and pCPRDEnv (Addgene plasmid 1732). In all cases plasmids (25 ng total) were transfected into HEK293T cells using 50 ul jetPEI in 500 ul total NaCl solution. Serum-containing DMEM was used at all times. Viral supernatants were harvested after 48, 72, and 96 hours post-transfection, pooled, filtered through a 40 μm syringe-driven filter (Millipore), concentrated 100x using the Lenti-X Concentrator system (Clontech), and stored as aliquots at -80°C. Concentrated lentiviral particles were then used to transduce $2x10^5$ target cells in 6-well dishes in the presence of 8 μg/ml polybrene in complete FAD overnight. Successfully infected cells were selected 48 hours post-transduction with either 2 ug/ml Puromycin, 150 ug/ml Hygromycin B, or Blasticidin (10 ug/ml) (Sigma).

SCC13, SJG15, SJG27 and SCC25 cells were first infected with YFP-luciferase virus followed by miRNA or cDNA vectors. To generate cDNA combination cell lines, YFP-luciferase expressing SCC13 cells were first infected with LeGO-iCer2-NUAK1 or LeGO-iCer2, sorted for Cerulean positivity by flow cytometry, and placed back in culture. These cells were then infected with LeGO-iC2-SPARC or LeGO-iC2, sorted for Cerulean and mCherry positivity and cultured. These double-cDNA cells were then infected with pLOC-LASP1 or pLOC-EV and treated with Blasticidin to select for triple-cDNA expressing cells. To generate pTRIPZ cells, YFP-luciferase expressing SCC13 cells were infected with pTRIPZ-SCR or pTRIPZ-miR-203 and selected with puromycin. Surviving clones were pooled and either treated with doxycycline for 3 days or not. Flow cytometry was used to isolate those cells that robustly induced tRFP expression upon doxycycline treatment;

uninduced cells remained uniformly tRFP negative. All cell sorting was run using a FACS Aria SORP (BD Biosciences).

RNA Extraction and qRT-PCR Expression Analysis

We curated miRNA expression data from 12 previously published studies using either primary HNSCC samples or cell lines for miRNAs coordinately deregulated in at least 3 of 12 reports (49, 50, 51, 52, 53, 54, 55, 56, 57, 58, 59, 60) The output list of 20 miRNAs was refined down to 15 miRNAs by excluding miRNAs with well-documented roles in cancer at the time of study initiation (miR-21, miR-31, miR-155, and the let-7 family) (61). miR-199b was removed from our candidate list given that the multiplexed primer pool used to assay miRNA expression did not feature a primer for this miRNA.

Total RNA was isolated using the miRNeasy Mini Kit (Qiagen) for both miRNA and mRNA expression analysis. For miRNA expression analysis, 500 ng input RNA was reverse transcribed using the TaqMan Megaplex Human Primer Pool A v2.1 and TaqMan MicroRNA Reverse Transcription Kit as per the manufacturer's instructions (Applied Biosystems). Expression of the relevant mature miRNA species was quantified by real-time PCR using individual TaqMan MicroRNAs Assays (Applied Biosystems) with the U6 snRNA as a normalization control. For mRNA expression analysis, 250 ng input RNA was reverse transcribed using the SuperScript III First-Strand Synthesis system (Invitrogen) according to the manufacturer's instructions. SYBR Green- or pre-designed TaqMan probe-based real-time PCR (Applied Biosystems) was used to amplify genes of interest with GAPDH or 18S ribosomal RNA serving as normalization controls, respectively. Data were acquired and analyzed using an ABI Prism 7900HT Sequence Detection System (Applied Biosystems). Relevant primer sequences are available in supplemental information.

Microarray Experiments and Gene Set Enrichment Analysis

Total RNA was isolated in technical duplicates from YFP-luciferase expressing SCC13 and SJG15 cells stably infected in biological duplicates with either miR-203 or scrambled control hairpin using a miRNeasy Mini Kit (Qiagen). RNA quality was analyzed using a Bioanalyzer 2100 (Agilent) and the RNA Nano chip.

Gene expression analysis was carried out on Illumina Human HT12 version 4 arrays. All data analyses were carried out on R using Bioconductor (62) packages. Raw intensity data from the array scanner were processed using the BASH (63) and HULK algorithms as implemented in the *beadarray* package (64). Log2 transformation and quantile normalization of the data were performed across all sample groups. Differential expression analysis was carried out using the *limma* package (65). Differentially expressed genes were selected using a p-value cut-off of <0.05 after application of FDR correction for multiple testing applied globally to correct for multiple contrasts. Gene expression data has been deposited into NCBI GEO and is accessible under accession number GSE47028.

GSEA and Leading Edge Analysis was performed using GSEA v2.0 software (Broad Institute) (66). Genes were ranked according to fold change from highest to lowest in miR-203 expressing SJG15 compared to control, or BMP2/7-treated primary human keratinocytes compared to control (33; GSE34558). The median value of probe sets present more than once was used and statistical significance assessed using 1000 random permutations of the gene set. An FDR q-value < 0.2 was considered significant. Gene sets were obtained from the MSigDB database v3.1 (September 2012 release) or from the published datasets indicated in the main text. Other datasets used but not shown are the following: melanoma (67, 68, 69), breast (70), prostate cancer (71), and endometroid endometrial tumors (72).

Luciferase Reporter Assays

HEK293T cells were seeded in white-coated 96 well plates (Corning) at a density of 10,000 cells/well overnight. 10ng psiCheck2-3'UTR vector was co-transfected with 20nM miRNA mimic molecules (Dharmacon) using jetPRIME (Polyplus), and incubated for 48 hours. Cells were subsequently lysed and a Dual-Luciferase assay (Promega) was performed using a PHERAstar *FS* multi-mode microplate reader (BMG LabTech) and standard protocols. Normalized luminescent signal (i.e. *Renilla*/*Firefly* ratio) in miRNA mimic transfected wells was compared to values from negative control miRNA transfected wells.

Animal Studies and Non-Invasive in vivo Bioluminescent Imaging

All animal work was subject to Cancer Research UK ethical review and performed in accordance with an approved U.K. Government Home Office license. Aged-matched (minimum 6 weeks of age) immunocompromized NOD/SCID/interleukin-2 gamma chain null (*Il2rg*$^{-/-}$) (NSG) mice (Jackson Labs; bred in-house) were inoculated via the tail vein with either 10^5 or $2.5\text{x}10^5$ SCC13 or SJG15 cells in 200 μL of PBS. For tongue xenografts, either 10^5 10^4, 10^3, 100 or 10 SCC13 cells in 50 μL of PBS were injected into the anterior dorsal mucosa of mice anaesthesized with isofluorane.

For bioluminescent imaging, mice were injected intraperitoneally with 200 μL D-luciferin dissolved in PBS (15mg/ml), anaesthetized with isofluorane, and imaged using a Xenogen IVIS 200 system 10 min after injection (PerkinElmer). Bioluminescent images were analyzed using tethered Living Image acquisition software (PerkinElmer). To produce BLI plots and associated graphs, total photon flux was calculated by drawing a region of interest across the thorax (experimental metastases) or head (tongue primary tumor growth) of each mouse individually in a supine position. This value was normalized to that obtained on the first day of scanning so that all mice began the experiment with an arbitrary starting BLI signal equal to 1.

Survival Analysis using Publically Available Microarray Datasets

NCBI GEO datasets GSE31056 (36) and GSE2379 (29) were downloaded as series matrix files of normalized expression values. Probe sets were matched to gene symbols for each platform. RNASeq (mRNA and miRNA) data from The Cancer Genome Atlas (TCGA) cohort of Head and Neck Cancer patients was downloaded as Level 3 data matrix files from the TCGA Data Portal available online (https://tcga-data.nci.nih.gov/tcga/). The BCGSC IlluminaHiSeq_miRNASeq (reads per million miRNA mapped) and the UNC IlluminaHiSeq_RNASeqV2 (normalized counts) data files were used for analysis. X-Tile software (73) was used to compute the optimal cutoff point for 2-population Kaplan-Meier analysis plotted using GraphPad Prism 6. Copy number aberrations called using gene- or cancer-centric GISTIC analysis of TCGA datasets were obtained through the TCGA Copy Number Portal available online (http://www.broadinstitute.org/tcga/home; 74).

Immunoblotting

For whole cell lysates, cells were grown to 70% confluency, washed twice with PBS, and harvested in RIPA buffer (Thermo Scientific). For secreted protein analysis, $5x10^6$ cells were seeded in 15 cm tissue culture places with 20ml serum-free FAD medium for 24 hours after which conditioned medium was harvested, filtered through a 0.45 μm syringe and concentrated using a 30 kDa Vivaspin 500 sample concentrator at full speed for 1 hour. Flow through was discarded and the remaining precipitate washed 3x for 30 minutes with 1M Tris-HCl (pH 8.0) followed by resuspension in RIPA buffer.

Protein lysates were sonicated for three 10-second cycles on medium power using a Biorupter (Diagenode) and sample concentration measured using a DC Protein Assay (Bio-Rad). 40 ug total protein per sample was separated on 4-12% polyacrylamide gradient gels, transferred to nitrocellulose membranes and detected using the following primary anti-human antibodies: LASP1 diluted 1:250 (Atlas HPA012072), NUAK1 diluted 1:250 (Atlas HPA027455), SPARC diluted 1:1,500 (Haemtech AON-5031), and GAPDH diluted 1:10,000 (Ambion AM4300). IRDye 800CW (800 nm channel) and 680LT (700 nm channel) secondary antibodies were used (LiCor). Western Blots were visualized using a Li-Cor Odyssey near infrared imager.

In vivo Intravasation

Total blood (w/heparin-lined syringe) was obtained by cardiac puncture under terminal anaesthesia from moribund mice that had received tongue xenografts of 10^4 SCC13-miR-203 and control cells. Total genomic DNA was extracted from 200 ul anti-coagulated whole blood per mouse using a QIAmp DNA Micro Kit (Qiagen) and diluted 1:20. The presence of circulating tumor cells was detected by SYBR Green qRT-PCR for the human repeat elements *AluJ* and *hLine1* (75), as well as the YFP-luciferase and Hygomycin genes integrated in luciferase-expressing cells.

Multi-Photon Confocal Microscopy

To quantitatively investigate *in vivo* extravasation potential, $2.5x10^5$ miR-203 and control SCC13 cells were tail-vein injected into NSG mice (*n*=3 per condition) and allowed to extravasate into the lungs. One hour prior to sacrifice, mice received an intravenous injection of 200 μl DyLight 594 labeled Tomato Lectin (*Lycopersicon*

Esculentum) (Vector Labs) to label the lung microvasculature. Whole lungs were surgically removed at necropsy after 30 hours of extravasation, washed in PBS, immobilized in an Ibidi μ-dish$^{35mm, high}$, and imaged as a wholemount with a Leica TCS SP5 confocal microscope equipped with a Chameleon Ultra laser (Coherent) for multi photon excitation using a 63x objective (HCX PL APO lambda blue 1.4 OIL). For excitation the laser was tuned to 835nm. An average of 25 lung areas were imaged per mouse, equating to 138 and 127 individual control and miR-203 expressing SCC13 cells per mouse, respectively. Images were processed with Volocity 3D Image Analysis 6.2 software (PerkinElmer) and Adobe Photoshop CS4.

miRNA in situ Hybridization

Formalin-fixed paraffin-embedded (FFPE) tissue blocks were sectioned at 10 μm on Fisher Superfrost slides and dried overnight at 45°C. After dewaxing and hydration through an ethanol series (100%, 70%, 50%), slides were incubated with 10ug/ml Proteinase K (Sigma Aldrich) for 8 min at 37°C. Slides were dehydrated in ethanol (70%, 90%, and 100%) and left to air dry. Double DIG-labeled LNA scrambled control and miR-203 probes (Exiqon) were diluted to 40nM in 2X hybridization buffer (Exiqon), denatured at 80°C for 4 min and chilled on ice. Probes were hybridized to tissue sections for 1 hour at 57°C using a hybridizer. Slides were put through a series of stringency washes with Saline-sodium citrate (SSC) and rinsed with PBS. Slides were then blocked for 30 min in antibody blocking buffer (10% sheep serum, 2% BSA in 1 X PBS) and incubated with anti-DIG-POD antibody (Roche; 1:200 in blocking buffer) for 1 hour at room temperature. Samples were washed in TNT buffer (0.1M Tris HCl pH 7.4, 0.15M NaCl, 0.05% Tween 20, 0.1% DEPC) and the Perkin Elmer TSA Cy3 kit reagents utilized to amplify and detect signal according to the manufacturer's protocol. Slides were mounted in Prolong Gold + DAPI and widefield fluorescence images of tissue sections were obtained with the Ariol automated microscope image capture system using filter sets for DAPI (nucleus) and Cy3 (miRNA) at fixed exposures.

Immunohistochemistry

FFPE specimens were de-waxed and rehydrated, using standard protocols, on the automated Leica ST5020. Antigen retrieval was done at 100°C in citrate buffer or

EDTA. The following primary antibodies were used: Ki67 (DAKO, clone MIB-1, 1:200), p63 (Novocastra, clone 7JUL, 1:50), EpCAM (Novocastra, clone VU-1D9, 1:100), Cytokeratin-14 (Novocastra, clone LL002, 1:20), Cytokeratin-5 (Novocastra, clone XM26, 1:100), E-Cadherin (DAKO, clone NCH-38, 1:25), SPARC (Haematologic Technologies, cat number AON-5031, 1:1500), Vimentin (Novocastra, clone SRL33, 1:400), N-Cadherin (Novocastra, clone IAR06, 1:100), Cleaved Caspase-3 (CC3) (Cell Signaling, cat number 9664, 1:100), Alpha 6 Integrin (Atlas, HPA012696, 1:140), Phospho-Histone H3 (PH3) (Ser10; Upstate, cat number 06-570, 1:500), GFP (Abcam, ab13970, 1;1000), CD31 (BD Pharmingen, clone MEC13.3, 1:100), LASP1 (Atlas, HPA012072, 1:500), NUAK1 (Atlas, HPA027455, 1:50), Keratin-10 (Covance, PRB-159P, 1:500), Loricrin (Covance, PRB-145P, 1:500), Involucrin (in-house, clone SY7, 1:500) and Keratin-14 (Covance, PRB-155P, 1:1000).

CC3, PH3, GFP, and CD31 were run on the LSAB system (Intense R) on the automated Bond Max (Leica Microsystems). All other antibodies were run on the polymer refine kit as per the manufacturer's instructions (Leica Microsystems).

Secondary antibodies (Jackson) were run at 1:250 in Bond diluent: biotinylated donkey anti-rat (ref. 712-065-153), biotinylated donkey anti-chicken (ref. 703-066-155), and biotinylated donkey anti-rabbit (ref. 711-065-152). The DAB Enhancer was used for all antibodies (Leica, ref. AR9432), as was the Avidin/Biotin blocking kit (Vector, ref. SP-2001).

Post-IHC hydration and clearing were performed on the automated Leica ST5020 and slides were mounted on Leica's CV5030. The automated ScanScope Digital Slide Scanner and software (Aperio) were used to image staining.

Graphing and Statistical Analysis

All graphs were generated using GraphPad Prism 6 and Adobe Illustrator CS4 software. Data are mean ± standard error of the mean (SEM). An unpaired two-tailed Student's t-test (*in vitro* experiments) or a non-parametric Mann-Whitney test (*in vivo* mouse experiments) was used for comparisons, with $P < 0.05$ considered significant. The Log-rank (Mantel-Cox) test was used to compare survival curves and compute Hazard Ratios, and a Fisher's exact test used to compare human cohorts on the basis of clinical characteristics.

Supplemental References

(49) Avissar, M., Christensen, B.C., Kelsey, K.T., and Marsit, C.J. (2009). MicroRNA expression ratio is predictive of head and neck squamous cell carcinoma. Clin. Cancer Res. *15*, 2850–2855.

(50) Childs, G., Fazzari, M., Kung, G., Kawachi, N., Brandwein-Gensler, M., McLemore, M., Chen, Q., Burk, R.D., Smith, R.V., Prystowsky, M.B., et al. (2009). Low-level expression of microRNAs let-7d and miR-205 are prognostic markers of head and neck squamous cell carcinoma. Am. J. Pathol. *174*, 736–745.

(51) Hebert, C., Norris, K., Scheper, M.A., Nikitakis, N., and Sauk, J.J. (2007). High mobility group A2 is a target for miRNA-98 in head and neck squamous cell carcinoma. Mol. Cancer *6*, 5.

(52) Henson, B.J., Bhattacharjee, S., O'Dee, D.M., Feingold, E., and Gollin, S.M. (2009). Decreased expression of miR-125b and miR-100 in oral cancer cells contributes to malignancy. Genes Chromosomes Cancer *48*, 569–582.

(53) Hui, A.B.Y., Lenarduzzi, M., Krushel, T., Waldron, L., Pintilie, M., Shi, W., Perez-Ordonez, B., Jurisica, I., O'Sullivan, B., Waldron, J., et al. (2010). Comprehensive MicroRNA profiling for head and neck squamous cell carcinomas. Clinical Cancer Research: An Official Journal of the American Association for Cancer Research *16*, 1129–1139.

(54) Kikkawa, N., Hanazawa, T., Fujimura, L., Nohata, N., Suzuki, H., Chazono, H., Sakurai, D., Horiguchi, S., Okamoto, Y., and Seki, N. (2010). miR-489 is a tumor-suppressive miRNA target PTPN11 in hypopharyngeal squamous cell carcinoma (HSCC). Br. J. Cancer *103*, 877–884.

(55) Kimura, S., Naganuma, S., Susuki, D., Hirono, Y., Yamaguchi, A., Fujieda, S., Sano, K., and Itoh, H. (2010). Expression of microRNAs in squamous cell carcinoma of human head and neck and the esophagus: miR-205 and miR-21 are specific markers for HNSCC and ESCC. Oncol. Rep. *23*, 1625–1633.

(56) Kozaki, K., Imoto, I., Mogi, S., Omura, K., and Inazawa, J. (2008). Exploration of tumor-suppressive microRNAs silenced by DNA hypermethylation in oral cancer. Cancer Res. *68*, 2094–2105.

(57) Ramdas, L., Giri, U., Ashorn, C.L., Coombes, K.R., El-Naggar, A., Ang, K.K., and Story, M.D. (2009). miRNA expression profiles in head and neck squamous cell carcinoma and adjacent normal tissue. Head Neck *31*, 642–654.

(58) Tran, N., McLean, T., Zhang, X., Zhao, C.J., Thomson, J.M., O'Brien, C., and Rose, B. (2007). MicroRNA expression profiles in head and neck cancer cell lines. Biochem. Biophys. Res. Commun. *358*, 12–17.

(59) Wald, A.I., Hoskins, E.E., Wells, S.I., Ferris, R.L., and Khan, S.A. (2011). Alteration of microRNA profiles in squamous cell carcinoma of the head and neck cell lines by human papillomavirus. Head & Neck *33*, 504–512.

(60) Wong, T.-S., Liu, X.-B., Wong, B.Y.-H., Ng, R.W.-M., Yuen, A.P.-W., and Wei, W.I. (2008). Mature miR-184 as Potential Oncogenic microRNA of Squamous Cell Carcinoma of Tongue. Clin. Cancer Res. *14*, 2588–2592.

(61) Ventura, A., and Jacks, T. (2009). MicroRNAs and cancer: short RNAs go a long way. Cell *136*, 586–591.

(62) Gentleman, R.C., Carey, V.J., Bates, D.M., Bolstad, B., Dettling, M., Dudoit, S., Ellis, B., Gautier, L., Ge, Y., Gentry, J., et al. (2004). Bioconductor: open software development for computational biology and bioinformatics. Genome Biology *5*, R80.

(63) Cairns, J.M., Dunning, M.J., Ritchie, M.E., Russell, R., and Lynch, A.G. (2008). BASH: a tool for managing BeadArray spatial artefacts. Bioinformatics (Oxford, England) *24*, 2921–2922.

(64) Dunning, M.J., Smith, M.L., Ritchie, M.E., and Tavaré, S. (2007). beadarray: R classes and methods for Illumina bead-based data. Bioinformatics *23*, 2183–2184.

(65) Smyth, G.K. (2004). Linear models and empirical bayes methods for assessing differential expression in microarray experiments. Stat Appl Genet Mol Biol *3*, Article3.

(66) Subramanian, A., Tamayo, P., Mootha, V.K., Mukherjee, S., Ebert, B.L., Gillette, M.A., Paulovich, A., Pomeroy, S.L., Golub, T.R., Lander, E.S., et al.

(2005). Gene set enrichment analysis: A knowledge-based approach for interpreting genome-wide expression profiles. PNAS *102*, 15545–15550.

(67) Winnepenninckx, V., Lazar, V., Michiels, S., Dessen, P., Stas, M., Alonso, S.R., Avril, M.-F., Ortiz Romero, P.L., Robert, T., Balacescu, O., et al. (2006). Gene expression profiling of primary cutaneous melanoma and clinical outcome. J. Natl. Cancer Inst. *98*, 472–482.

(68) Jaeger, J., Koczan, D., Thiesen, H.-J., Ibrahim, S.M., Gross, G., Spang, R., and Kunz, M. (2007). Gene expression signatures for tumor progression, tumor subtype, and tumor thickness in laser-microdissected melanoma tissues. Clin. Cancer Res. *13*, 806–815.

(69) Alonso, S.R., Tracey, L., Ortiz, P., Pérez-Gómez, B., Palacios, J., Pollán, M., Linares, J., Serrano, S., Sáez-Castillo, A.I., Sánchez, L., et al. (2007). A high-throughput study in melanoma identifies epithelial-mesenchymal transition as a major determinant of metastasis. Cancer Res. *67*, 3450–3460.

(70) Ramaswamy, S., Ross, K.N., Lander, E.S., and Golub, T.R. (2003). A molecular signature of metastasis in primary solid tumors. Nat. Genet. *33*, 49–54.

(71) Chandran, U.R., Ma, C., Dhir, R., Bisceglia, M., Lyons-Weiler, M., Liang, W., Michalopoulos, G., Becich, M., and Monzon, F.A. (2007). Gene expression profiles of prostate cancer reveal involvement of multiple molecular pathways in the metastatic process. BMC Cancer *7*, 64.

(72) Bidus, M.A., Risinger, J.I., Chandramouli, G.V.R., Dainty, L.A., Litzi, T.J., Berchuck, A., Barrett, J.C., and Maxwell, G.L. (2006). Prediction of lymph node metastasis in patients with endometrioid endometrial cancer using expression microarray. Clin. Cancer Res. *12*, 83–88.

(73) Camp, R.L., Dolled-Filhart, M., and Rimm, D.L. (2004). X-Tile A New Bio-Informatics Tool for Biomarker Assessment and Outcome-Based Cut-Point Optimization. Clin Cancer Res *10*, 7252–7259.

(74) Mermel, C.H., Schumacher, S.E., Hill, B., Meyerson, M.L., Beroukhim, R., and Getz, G. (2011). GISTIC2.0 facilitates sensitive and confident localization of the targets of focal somatic copy-number alteration in human cancers. Genome Biol. *12*, R41.

(75) Gorges, T.M., Schiler, J., Schmitz, A., Schuetzmann, D., Schatz, C., Zollner, T.M., Krahn, T., von Ahsen, O. (2012) Cancer therapy monitoring in xenografts by quantitative analysis of circulating tumor DNA. Biomarkers. *17*, 498-506.
