# Supplementary material for: Rewiring of an Epithelial Differentiation Factor, miR-203, to Inhibit Human Squamous Cell Carcinoma Metastasis
Source: Cell Rep. 2014 Oct 2;9(1):104–17. doi: 10.1016/j.celrep.2014.08.062 (PMC4536294; doi:10.1016/j.celrep.2014.08.062)
Supplement: Document S2. Article plus Supplemental Information [file mmc2.pdf]

Article

Cell Reports

Rewiring of an Epithelial Differentiation Factor, miR-203, to Inhibit Human Squamous Cell Carcinoma Metastasis

Graphical Abstract

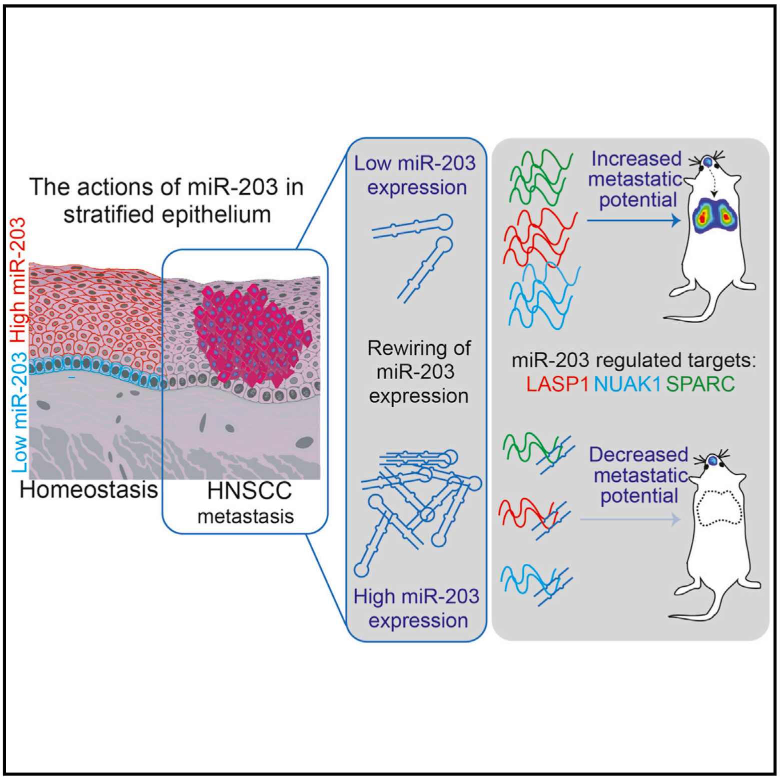

Authors

Nathan Benaich, Samuel Woodhouse, ..., Sven R. Quist, Fiona M. Watt

Correspondence

fiona.watt@kcl.ac.uk

In Brief

Benaich et al. have identified miR-203, a microRNA that triggers differentiation in multilayered epithelia, as an inhibitor of lung metastasis in head and neck squamous cell carcinoma (HNSCC) cells. They show that miR-203 inhibits metastasis independently of its effects on differentiation. Rather, miR-203 suppresses the prometastatic activities of three factors involved in cytoskeletal dynamics (LASP1), extracellular matrix remodeling (SPARC), and cell metabolism (NUAK1). Expression of miR-203 and its downstream effectors correlates with survival in HNSCC patients.

Accession Numbers

GSE47028

Highlights

miR-203 regulates in vivo lung metastasis without triggering differentiation

Restoring miR-203 in already established metastases elicits regression

LASP1, NUAK1, and SPARC are downstream prometastatic effectors of miR-203

The miR-203-LASP1/SPARC/NUAK1 axis is prognostic of overall survival in HNSCC

Benaich et al., 2014, Cell Reports 9, 104–117
October 9, 2014 ©2014 The Authors
http://dx.doi.org/10.1016/j.celrep.2014.08.062

Rewiring of an Epithelial Differentiation Factor, miR-203, to Inhibit Human Squamous Cell Carcinoma Metastasis

Nathan Benaich,[1,2,4] Samuel Woodhouse,[2,4] Stephen J. Goldie,[1] Ajay Mishra,[2] Sven R. Quist,[1,3] and Fiona M. Watt[2,*]
[1]Cancer Research UK Cambridge Institute, University of Cambridge, Li Ka Shing Centre, Robinson Way, Cambridge CB2 0RE, UK
[2]Centre for Stem Cells and Regenerative Medicine, King's College London, 28th Floor, Tower Wing, Guy's Hospital, Great Maze Pond, London SE1 9RT, UK
[3]Clinic of Dermatology and Venereology, Otto-von-Guericke University, Magdeburg, Leipziger Strasse 44, 39120 Magdeburg, Germany
[4]Co-first author
*Correspondence: fiona.watt@kcl.ac.uk
http://dx.doi.org/10.1016/j.celrep.2014.08.062
This is an open access article under the CC BY license (http://creativecommons.org/licenses/by/3.0/).

SUMMARY

Metastatic colonization of distant organs underpins the majority of human-cancer-related deaths, including deaths from head and neck squamous cell carcinoma (HNSCC). We report that miR-203, a miRNA that triggers differentiation in multilayered epithelia, inhibits multiple postextravasation events during HNSCC lung metastasis. Inducible reactivation of miR-203 in already established lung metastases reduces the overall metastatic burden. Using an integrated approach, we reveal that miR-203 inhibits metastasis independently of its effects on differentiation. In vivo genetic reconstitution experiments show that miR-203 inhibits lung metastasis by suppressing the prometastatic activities of three factors involved in cytoskeletal dynamics (LASP1), extracellular matrix remodeling (SPARC), and cell metabolism (NUAK1). Expression of miR-203 and its downstream effectors correlates with HNSCC overall survival outcomes, indicating the therapeutic potential of targeting this signaling axis.

INTRODUCTION

Adult stratified epithelia are maintained by a balance between stem cell self-renewal and differentiation (Arwert et al., 2012; Blanpain and Fuchs, 2006). As emerging neoplastic drivers, stem cells and the factors that control their biology are of therapeutic relevance in carcinomas (Pardal et al., 2003). For example, *NOTCH1* and *TP63* form a negative feedback loop in epidermal stem cells, with *NOTCH1* promoting differentiation and *TP63* inhibiting it (Nguyen et al., 2006). Inactivation of these genes is associated with skin tumors in mice (Flores et al., 2005; Nicolas et al., 2003) and head and neck squamous cell carcinoma (HNSCC) in humans (Agrawal et al., 2011; Stransky et al., 2011). Thus, disruption of the epithelial stem cell molecular circuitry can play a driving role in malignant transformation of the tissues they replenish.

HNSCC is the sixth most common cancer worldwide and has had a 5-year overall survival rate of only ~50% for decades (Leemans et al., 2011). Two-thirds of patients present with advanced, locally invasive disease that recurs despite mainstay surgery or chemo- and/or radiotherapy, thus creating a pressing need for novel avenues of therapeutic intervention (Argiris et al., 2008).

Metastasis accounts for >90% of solid-cancer-related deaths (Valastyan and Weinberg, 2011). Metastatic dissemination can occur early in the evolution of a tumor, followed by extended dormancy (Hüsemann et al., 2008). Indeed, up to 40% of carcinoma cases without clinical evidence of metastasis actually harbor disseminated tumor cells in the bone marrow (Pantel and Brakenhoff, 2004). Thus, truly efficacious cancer therapeutics must target already established metastases rather than just inhibit tumor growth or dissemination (Valastyan and Weinberg, 2011).

miRNAs are small noncoding RNAs that posttranscriptionally repress target mRNAs important for tissue homeostasis and cancer (Lujambio and Lowe, 2012; Valastyan et al., 2009b). Although our understanding of metastasis-relevant miRNAs has advanced rapidly in well-studied malignancies such as breast cancer (Valastyan et al., 2009a, 2010, 2011; Yi et al., 2008), we know little about whether and how miRNAs modulate metastasis in HNSCC. Therefore, we employed functional in vivo approaches to identify miR-203 as a potent negative regulator of HNSCC metastasis by targeting a panel of prometastatic effector proteins (Yi et al., 2008).

RESULTS

A Screen of miRNAs in HNSCC Identifies miR-203 as a Metastasis Suppressor

To uncover endogenous miRNAs that reduce the lung metastatic potential of HNSCC, we employed the screening approach shown in Figure 1A. Using a panel of 17 primary, early-passage human HNSCC cell lines from surgically resected tumors, we assayed the expression of 15 miRNAs identified as coordinately deregulated in published expression profiles of HNSCC (see the Supplemental Experimental Procedures). We identified five downregulated miRNAs (miR-26b, miR-125b, miR-203,

miR-218, and miR-373) and one upregulated miRNA (miR-15a) when we compared miRNA expression in HNSCC cells versus primary human keratinocytes (Figure 1A). miR-133a and miR-133b were not detected in any lines.

To assess the function of the deregulated miRNAs in HNSCC, we generated two YFP-luciferase-expressing cell lines—SCC13 (established facial SCC; Rheinwald and Beckett, 1981) and SJG15 (primary lingual SCC; Goldie et al., 2012)—in which we knocked down miR-15a or stably overexpressed miR-26b, miR-125b, miR-203, miR-218, or miR-373 using lentiviral approaches (Figure S1A). No antiproliferative or cytotoxic effects were observed in vitro in adherent cultures (Figure S1C).

We explored the function of these miRNAs in an in vivo orthotopic tongue xenograft assay that recapitulates aspects of human HNSCC (Goldie et al., 2012). SCC13 cells were injected into the lingual mucosa of nonobese diabetic/severe combined immunodeficient interleukin-2 receptor-γ chain null (NSG) mice (Figure 1A), and noninvasive, luciferase-based bioluminescent imaging was used to monitor disease progression (Kim et al., 2010; Tiffen et al., 2010). Xenografting 10^5 miRNA expressing SCC13 cells revealed that overexpression of miR-203 and knockdown of miR-15a modestly reduced tumor burden after 26 days compared with control. The other miRNAs did not affect tumor burden (Figures 1B and S1D).

To measure metastatic dissemination from the primary tumors, we imaged the lungs of tumor-bearing mice ex vivo at 26 days (Figure 1C). Overexpression of miR-26b and miR-218 enhanced metastatic dissemination, whereas the other four miRNAs had no significant effect (Figure 1D). Since the aim of the screen was to identify antimetastatic miRNAs, we did not analyze miR-26b and miR-218 further.

To examine metastatic colonization independently of primary tumor growth, we intravenously injected miRNA-expressing SCC13 and SJG15 cells into NSG mice (Figure 1A). Overexpression of miR-203 or knockdown of miR-15a suppressed metastasis in both cell lines, as determined by endpoint lung metastatic burden, ex vivo fluorescence microscopy, and histology (Figures 1E and S1E–S1H). miR-125b and miR-373 were excluded from further analysis because their effects were not consistent between SCC13 and SJG15 cells. Since miR-15a is a tumor suppressor (Aqeilan et al., 2010), we expressed miR-15a in otherwise nonmetastatic, miR-15a^{low} SCC25 cells (established lingual SCC; Rheinwald and Beckett, 1981; Figure S1B). Overexpression of miR-15a did not enhance lung metastatic colonization by SCC25 cells (Figure 1F), so we did not analyze this miR further.

With miR-203 presenting the most compelling case as a metastasis-suppressor gene, we explored its clinical relevance in a cohort of 219 HNSCC patients from The Cancer Genome Atlas (TCGA) (data available through the TCGA Data Portal; Figure 1G). The 5-year survival rate of miR-203-low patients was 36%, compared with 70% for miR-203-high patients, independently of primary tumor size (T1/T2 versus T3/T4). Patients with miR-203-low tumors also exhibited significantly more lymph node-positive disease and lymphovascular invasion—two major prognostic factors for eventual distant metastasis. Hence, miR-203 is a prognostic indicator of overall survival and propensity for metastasis.

Using high-resolution copy-number data for 322 HNSCC patients from TCGA, we detected a copy-number alteration (CNA) harboring miR-203 that was focally deleted in 15% of cases (Figure S2B). This CNA included several genes that have been implicated in suppressing epithelial tumorigenesis and/or metastasis, such as DICER1 (Martello et al., 2010) and ELF5 (Chakrabarti et al., 2012). The miR-203 locus was also deleted in cutaneous melanoma (34% of cases), lung adenocarcinoma (29% of cases), and glioblastoma multiforme (28% of cases) (Figure S2C). Therefore, deregulation of miR-203 in HNSCC (and potentially other tumor types) is due, at least in part, to focal CNAs that alter multiple tumor- and/or metastasis-suppressive pathways.

Effect of miR-203 Expression on Primary Tumor Growth

To examine miR-203 expression in vivo, we performed in situ hybridization in 28 cases of primary and/or metastatic skin SCCs with matched normal skin from the same patients, and three further cases of skin SCC (total of 11 patients; Table S1). Whereas miR-203 was strongly expressed in the suprabasal, differentiated layers of normal epidermis, confirming previous reports (Yi et al., 2008), its expression was 35-fold lower in matched cutaneous SCCs (Figure 2A).

We found that miR-203 significantly reduced both the average number and size of spheres formed by SCC13 in soft agar (Figure 2B and data not shown), consistent with the observation that when 10^5 cells were injected into the tongue, miR-203 reduced tumor growth (Figure 1B). To assess the impact on tumor growth in more detail, we xenografted 10^4 SCC13 cells expressing miR-203 or scrambled control hairpin (SCR) and monitored tumors for up to 50 days (Figure 2C). We confirmed miR-203 expression by in situ hybridization (Figure S3B). The reduction in tumor size upon expression of miR-203 was due to a lag in the initial rate of growth, and from week 2 onward the rate of tumor growth was comparable to that in control SCC13 cells (Figures 2C and S3A).

To investigate whether miR-203 affected the proportion of cells that were capable of initiating primary tumors, we performed a limiting dilution analysis (LDA) (Lapouge et al., 2012) in which 10^4, 10^3, 100, or 10 SCC13 SCR or miR-203 cells were injected into the tongue (Figure 2D). No difference in tumor incidence was observed between miR-203- and SCR-expressing cells (Figure 2D). There was, however, a lag in tumor growth phase at all seeding densities (Figures 2C and S1D; data not shown).

We conclude that miR-203 overexpression does not affect the number of tumor-initiating cells or the overall tumor growth rate, but causes a delay before tumor growth becomes established, which results in a reduction in tumor size. This may explain the lack of a correlation between T stage and miR-203 expression in HNSCC patients, since, at least in xenografts, the effect on tumor size is more marked at early time points (Figure 1G).

miR-203 Expression Inhibits Lung Metastasis

We measured the circulating tumor DNA content by extracting whole peripheral blood 50 days after tongue xenografting (Figure 2C) and determined the levels of human *AluJ* or *hLine1* repeat elements by quantitative PCR (qPCR) (Figure 2E). There was no difference in circulating tumor DNA content between

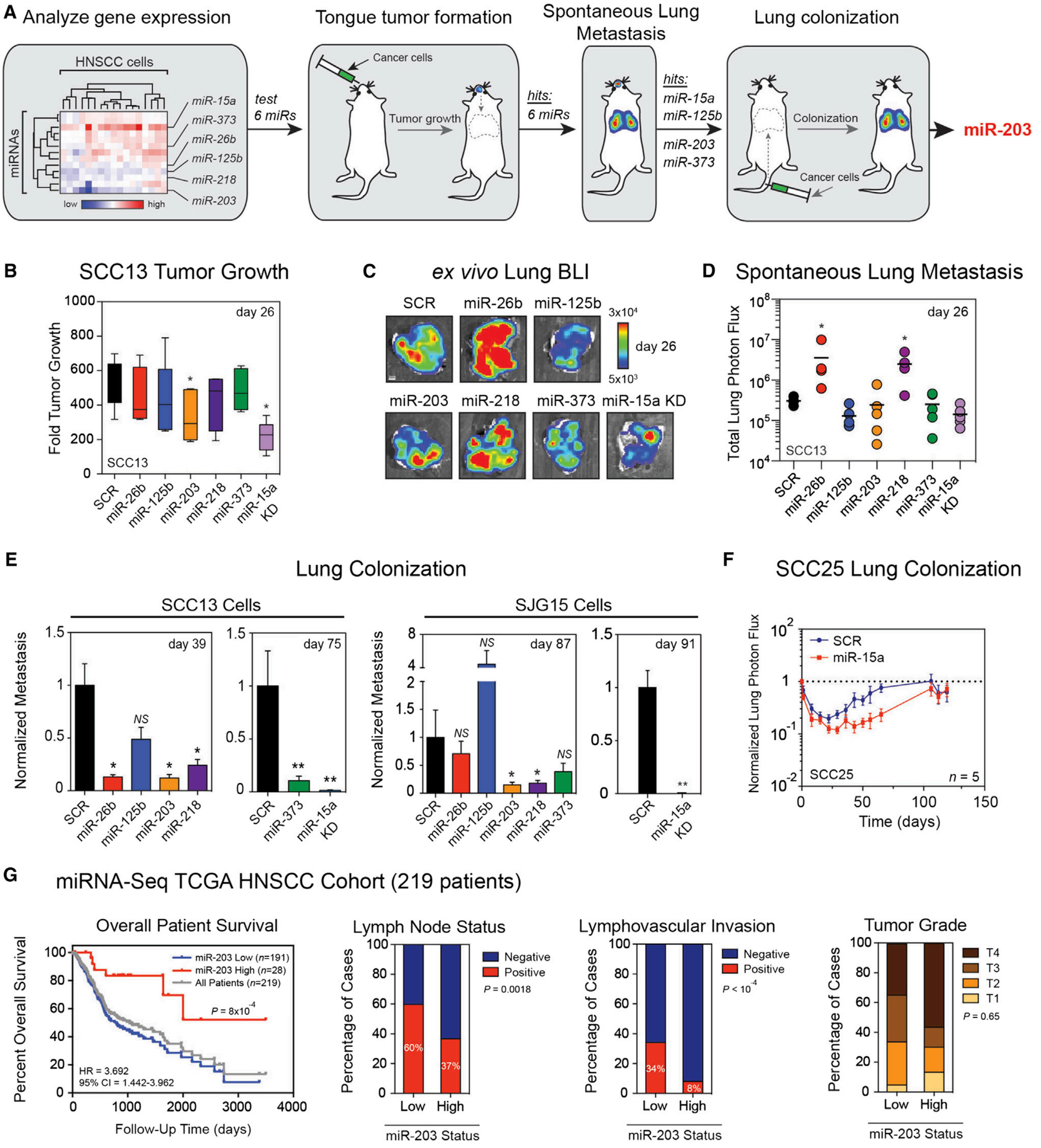

Figure 1. Candidate-Gene-Based Functional In Vivo miRNA Screen

(A) Schematic of the pipeline for an in vivo functional screen to identify miRNAs that regulate HNSCC lung metastasis. Heatmap of $\log_2$ normalized qRT-PCR expression data for 13 miRNAs in 17 human HNSCC lines normalized to normal human oral keratinocytes. Data were clustered using cosine statistics.

(B) Fold primary tumor growth generated by 10^5 SCC13 cells individually expressing the indicated miRNA vectors after 26 days. Whiskers indicate min/max and the horizontal bar is the median, with n = 4–5 per group.

(C) Representative ex vivo bioluminescent images of whole lungs at necropsy (day 26). Scale bar represents 3 mm.

(D) Total ex vivo lung photon flux at endpoint (day 26). The horizontal line indicates mean, with n = 5 per group.

(E) Lung metastatic burden resulting from tail-vein injection of SCC13 or SJG15 cells in which the levels of six miRNAs were individually modulated. Data are means ± SEM, n = 5 per group.

(legend continued on next page)

mice bearing miR-203 or control SCC13 primary tumors (Figures 2E and S3C). However, ex vivo bioluminescence and fluorescence imaging of lungs showed that miR-203-expressing SCC13 cells founded smaller metastases than control cells (Figure 2F). Therefore, miR-203 does not alter the ability of cells from the primary tumor to enter the circulation, but does hamper their capacity to expand following engraftment in the lungs.

Consistent with these results, miR-203-expressing SCC13 and SJG15 cells exhibited a dose-dependent impairment in lung colonization following tail-vein injection (Figure 2G). Expression of miR-203 significantly prolonged the time required for SCC13 and SJG15 cells to reach input levels of lung bioluminescence, suggesting that miR-203 extended the dormancy phase of engrafted cells (Figure 2G), mirroring the phenotype observed in primary tumors (Figure 2C). Expression of miR-203 significantly increased survival time (Figure 2H) and reduced the endpoint lung metastatic burden (Figure 2I). This was confirmed by ex vivo fluorescence microscopy and anti-keratin-14 immunohistochemistry (IHC) of lungs (Figure 2J and S3E). Whereas control SCC13 and SJG15 cells colonized the majority of the lung area, cells expressing miR-203 only generated small metastatic lesions or remained as clusters of individual cells (Figure S3F). We confirmed that miR-203 expression was sustained in SCC13 lung metastases (Figure S3G). Taken together, these data suggest that miR-203 regulates the outgrowth of tumor cells once they have reached the lung.

We next asked whether endogenous miR-203 was necessary to prevent metastasis. For this purpose, we used SJG27 cells (primary lingual SCC), which express high levels of miR-203 and do not readily metastasize. Silencing miR-203 (miR-203 KD) in YFP-luciferase-labeled SJG27 cells did not impair proliferation in vitro (Figures S3H and S3I), but did enhance formation of clonogenic spheres in soft agar (Figure 3A). When injected into the circulation of mice, miR-203 KD SJG27 cells were significantly more proficient at forming lung metastases over 65 days compared with control cells (Figure 3B). Knockdown of miR-203 reduced the time to metastatic progression (Figure 3C) and enhanced the total lung metastatic burden (Figure 3D). Ex vivo fluorescence microscopy and anti-keratin-5 IHC showed that miR-203 KD cells generated more numerous and larger nodules than control cells (Figure 3E). Clusters of individual cells were visible in control, but not miR-203 KD, lungs, further suggesting a role for miR-203 in maintaining dormancy (Figure S3J).

We conclude, on the basis of miR-203 knockdown and overexpression, that miR-203 inhibits lung metastasis of HNSCC.

miR-203 Regulates Multiple Postextravasation Events and Inhibits Established Metastases

We next used a doxycycline (dox)-inducible system to temporally control miR-203 expression (Figures 4A, 4B, and S4A). Dox-inducible miR-203 expression did not affect in vitro proliferation of adherent cells (Figure S4B), but reduced the anchorage-independent growth of SCC13 cells in soft agar (Figure S4C), consistent with the effect of constitutive expression (Figure 2B).

We activated miR-203 expression at defined time points following intravenous injection of SCC13 cells (Figure 4B). Expression of miR-203 was either never induced (mimicking cells lacking miR-203), induced before intravenous injection (day 0, phenocopying constitutive miR-203 expression), or induced 10 (dormancy phase of engrafted cells), 29 (establishment of micrometastases), or 56 days (macrometastatic colonization) after injection.

Animals injected with scrambled control cells that were never induced or induced from day 0, 10, 29, or 56 all had similar disease burdens, arguing against any effects of the dox-rich diet (Figures 4C–4E and data not shown). An examination of RFP and GFP signals from lung metastases in each cohort of animals, as well as in situ hybridization for miR-203, confirmed dox-dependent, sustained expression of miR-203 in vivo (Figures S4D–S4F and data not shown).

Activation of miR-203 for the duration of the experiment resulted in a 95% inhibition of endpoint lung metastatic burden (Figures 4C and 4D). Reexpression of miR-203 from 10 or 29 days was sufficient to block 80% of the endpoint lung metastatic burden (Figures 4C and 4D). Ex vivo fluorescence microscopy of lungs confirmed that reintroduction of miR-203 substantially reduced the number and size of lung metastases (Figure 4E). Although it was not statistically significant, animals in which miR-203 was induced for only the final 2 weeks of the experiment had ~40% lower lung metastasis (Figures 4C and 4D) accompanied by a moderate reduction in lesion size and number (Figure 4E).

We next investigated whether miR-203 affects cancer cell extravasation across the lung vasculature in vivo (Figure 4F). Thirty hours after intravenous injection, multiphoton confocal imaging of individual GFP^+ tumor cells and tomato lectin-labeled blood vessels revealed that a similar proportion (~90%) of control and miR-203-expressing SCC13 cells had successfully extravasated (Figure 4G).

We conclude that miR-203 inhibits lung metastasis by inhibiting exit from dormancy, rather than by preventing migration from the primary tumor or entry into the lung.

miR-203 Induces Mutually Exclusive Transcriptomic Landscapes Characteristic of Keratinocyte Differentiation and Poorly Metastatic Cells

To probe the mechanisms by which miR-203 regulates metastasis, we performed genome-wide expression profiling and gene set enrichment analysis (GSEA) of miR-203-expressing SCC13 and SJG15 cells versus scrambled controls. Genome-

(F) Time course of experimental lung metastasis by SCC25 cells overexpressing miR-15a or control vector for 119 days. Log_{10} y axis, data are means ± SEM, n = 5 per group.

(G) Kaplan-Meier plots for overall survival of 219 HNSCC patients from the TCGA cohort partitioned into miR-203-high (red, n = 28) and miR-203-low (blue, n = 191) cohorts based on miRNAseq read counts. The miR-203-high and -low patient cohorts were analyzed for the proportion of cases with lymph node disease (positive or negative), lymphovascular invasion (positive or negative), and primary tumor T-stage, which is a measure of tumour state and size.

*p < 0.05; **p < 0.01; ***p < 0.001 (nonparametric Mann-Whitney test). For Kaplan-Meier plots, p values were calculated using a log rank Mantel-Cox test. For patient cohort comparisons, p values were calculated using Fisher's exact test. See also Figure S1.

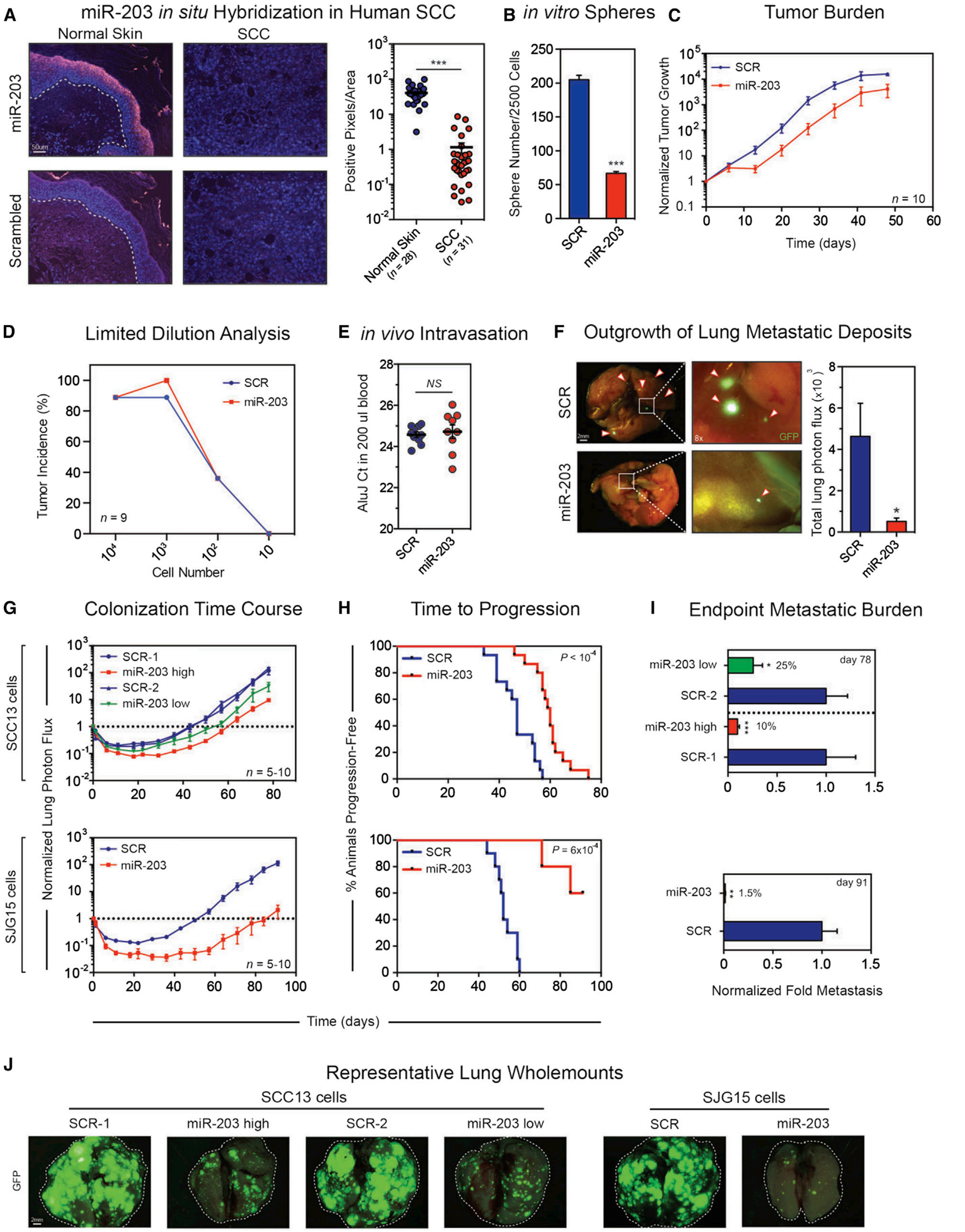

(legend on next page)

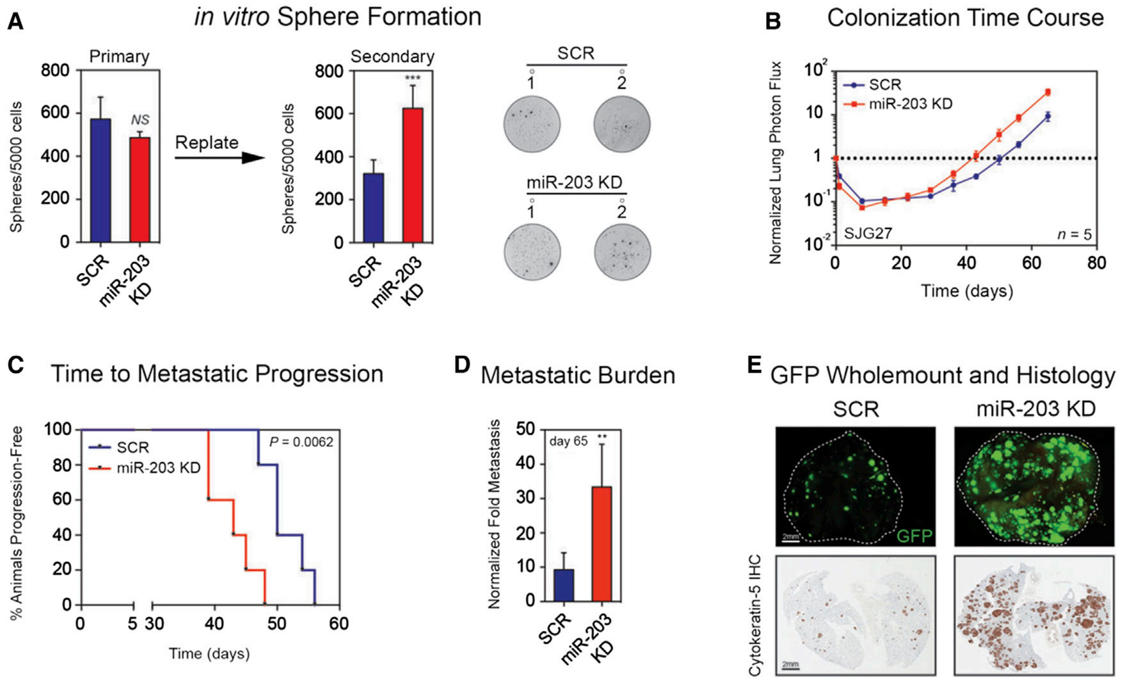

Figure 3. Silencing Endogenous miR-203 Enhances Lung Metastasis

(A) In vitro soft-agar assay quantified at the first and second passages. Data are means ± SEM, with representative wells shown; ***$p < 0.001$ calculated using two-tailed Student's t test.

(B) Time course of experimental lung metastasis of miR-203 knockdown (KD) and control SJG27 cells over 65 days. Log_{10} y axis; data are mean ± SEM; n = 5 per group.

(C) Kaplan-Meier plots for time to progression of lung metastasis of SJG27 SCR and miR-203-KD groups; p values were calculated using the log rank Mantel-Cox test.

(D) Lung metastasis by control and miR-203-KD SJG27 cells at day 65. Data are mean ± SEM; n = 5 per group; **$p < 0.01$ calculated using the nonparametric Mann-Whitney test.

(E) Representative whole-mount GFP fluorescence microscopy images and matched keratin-5 IHC of metastatic lungs. Scale bar represents 2 mm.

See also Figure S3.

wide expression changes elicited by miR-203 were negatively enriched for signatures of metastasis in primary human HNSCC arising in various anatomical locations within the aerodigestive tract (Cromer et al., 2004; O'Donnell et al., 2005; Rickman et al., 2008; Figure 5A). A similar negative enrichment between miR-203 and metastasis signatures was also found in

Figure 2. miR-203 Inhibits Experimental Lung Metastasis

(A) Representative in situ hybridization (red signal) for miR-203 and scrambled control probe in normal human skin (n = 28) and malignant SCC (n = 31) counterstained with DAPI nuclear label. Scale bar represents 50 μm. Adjacent panel: quantification of miR-203 signal intensity in normal skin versus SCC. Log_{10} y axis.

(B) Anchorage-independent soft-agar assay. GFP^+ spheres were quantified after 3 weeks. Data are means ± SEM.

(C) Fold primary tumor growth: 10^4 control and miR-203 expressing SCC13 cells were injected into the tongue and tumor growth was monitored by bioluminescent imaging. Raw photon flux was normalized to input levels and presented as mean ± SEM; n = 10 per group.

(D) Limited dilution analysis: 10^4, 10^3, 100, and 10 control and miR-203-expressing SCC13 cells were injected into the tongue. Tumor incidence (5-fold increase over baseline) was determined by bioluminescent imaging and tumors were monitored for up to 70 days (n = 9).

(E) In vivo tumor cell intravasation was inferred by using qRT-PCR for human-specific AluJ repeat elements present in circulating tumor cells within 200 μl whole blood. n = 10 (SCR), n = 9 (miR-203).

(F) Whole-mount lung fluorescence images from representative animals in (D). Scale bar represents 2 mm; left panels at 2× and right panels at 8× magnification. Quantification of lung metastasis by bioluminescence; data are means ± SEM; n = 7 (SCR), n = 6 (miR-203).

(G) Lung metastatic colonization time course for SCC13 cells expressing high (n = 10) or low miR-203 (n = 5) and relevant controls, and SJG15 cells expressing miR-203 or scrambled control hairpin (n = 5 each). Log_{10} y axis. Data are means ± SEM.

(H) Kaplan-Meier analysis for time to progression of lung metastasis. SCC13 SCR (n = 15) and miR-203 groups (high and low; n = 15) were pooled.

(I) Endpoint lung metastatic burden of animals from (F) injected with miR-203 expressing SCC13 cells (day 78) and SJG15 cells (day 91) normalized to control animals. Data are means ± SEM.

(J) Representative whole-mount GFP fluorescence microscopy images of metastatic lung fields generated by miR-203 and control SCC13 and SJG15 cells. Scale bar represents 2 mm.

*$p < 0.05$; **$p < 0.01$; ***$p < 0.001$ calculated using the nonparametric Mann-Whitney test. The p values for the Kaplan-Meier plots were calculated using the log rank Mantel-Cox test. The p values for the soft-agar assay were calculated using the two-tailed Student's t test. See also Figures S2 and S3.

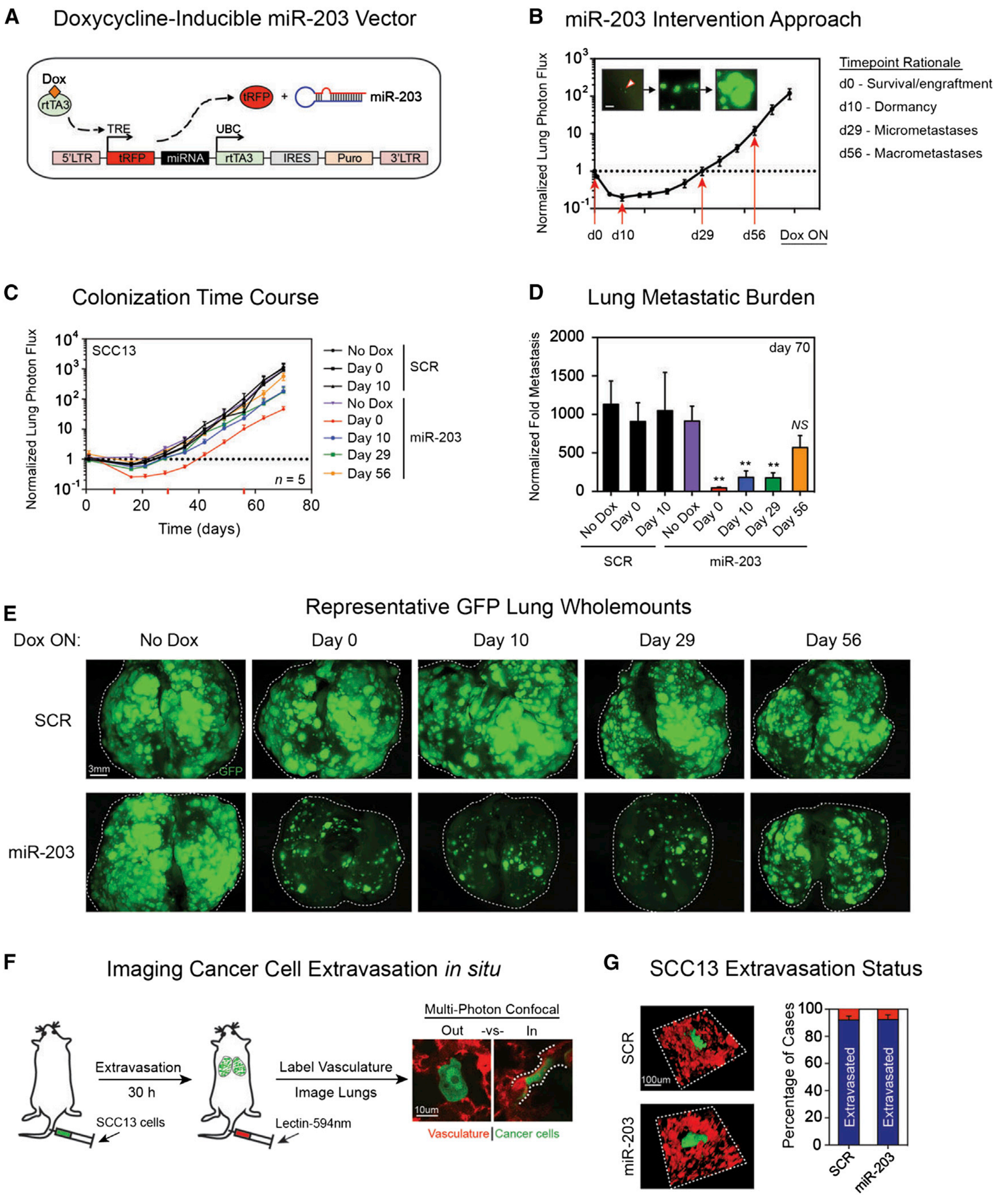

Figure 4. Inducible Restoration of miR-203

(A) Schematic representation of the dox-inducible miR-203 vector.

(B) Dox-mediated approach for reexpressing miR-203 following intravenous inoculation of SCC13 cells. Images represent single cells, micrometastases, and macrometastases (left to right) in the lung following tail-vein injection. Scale bar represents 500 μm.

(C) Time course of experimental lung metastases formed by inducible control (SCR) and miR-203-expressing SCC13 in animals on normal or dox-rich diets. Day indicates when the diet was switched to dox-rich. Log_{10} y axis; data are means ± SEM; n = 4–5 per group.

(D) Lung metastasis at endpoint (day 70) of noninduced or induced control and miR-203 SCC13 groups. Data are means ± SEM; n = 4–5 per group; **$p < 0.01$ calculated using nonparametric Mann-Whitney test relative to noninduced miR-203 animals. Differences between SCR groups and noninduced miR-203 are nonsignificant.

(E) Representative whole-mount ex vivo GFP fluorescence microscopy images of metastatic lungs at day 70. Scale bar represents 3 mm.

(legend continued on next page)

melanoma, breast, prostate, and endometrial tumors, all of which have a tendency to metastasize (data not shown). Together, these results suggest that miR-203 enforces a transcriptional landscape in human HNSCC cells that is restrictive for metastasis.

GSEA also revealed that signatures of human keratinocyte differentiation were positively enriched in miR-203-expressing HNSCC cells (Kretz et al., 2012; Mulder et al., 2012; Sen et al., 2010; Figures 5A and 5B). However, there was no overlap between the core genes driving the metastasis and differentiation signatures (Figure 5C). This suggests that miR-203 uses mutually exclusive genetic strategies to prime the transcriptional landscape of HNSCC cells toward a poorly metastatic cell state and a differentiated state. In support of this prediction, there was no difference between control and miR-203-expressing SCC13 tongue tumors and lung metastases in expression of markers of undifferentiated keratinocytes, including keratin-5, keratin-14, α6-integrin, and p63. There was no difference in E-cadherin expression, and all tumors were negative for the epithelial-mesenchymal transition (EMT) markers N-cadherin and vimentin, with the exception of a few cells at the invasive edge (Figures S5A and S5B; cf. Taube et al., 2013). There was also no difference in expression of the keratinocyte differentiation markers involucrin, keratin 10, and loricrin (Figures S5C and S5D), or in markers of proliferation (Ki67 and phospho-histone H3), apoptosis (cleaved-caspase 3 positive), or vascular density (Figures S6A–S6C).

Hence, although miR-203 primed the transcriptional landscape of HNSCC cells toward a differentiated state, primary tumors and lung metastases did not exhibit alterations in differentiation status in vivo. These results suggest that the mechanism by which miR-203 controls metastasis is distinct from its prodifferentiation function in normal epidermis (Yi et al., 2008).

miR-203 Directly Targets a Cohort of Genes Upregulated in HNSCC

To identify prometastatic miR-203 target genes, we used an integrated genomics, bioinformatics, and experimental approach (Figure 5D). We curated our microarray expression data for genes significantly downregulated by miR-203 and overlapped these hits (171 genes) with a list of 993 miR-203 predicted target genes from 11 publically available algorithms. To enrich for relevance in human HNSCC, we filtered the 13 hits through Oncomine (Rhodes et al., 2007) and the Human Protein Atlas (Uhlen et al., 2010), and discovered four genes (*LASP1*, *NUAK1*, *SPARC*, and *THBS2*) that were upregulated in HNSCC specimens relative to normal mucosa. Furthermore, we observed tumor-specific upregulation of *LASP1*, *NUAK1*, *SPARC*, and *THBS2* in an independent cohort of 24 oral SCC patients not contained in Oncomine (Figure 5F; Rhodes et al., 2007). Genes associated with the actions of miR-203 in other cancers, such as BIRC5 (Saini et al., 2011), SNAI2 (Ding et al., 2013), P63 (Yi et al., 2008), and c-Jun (Sonkoly et al., 2012), were not enriched in our samples.

To validate the results of our analysis, we showed that overexpression of miR-203 led to downregulation of LASP1, NUAK1, SPARC, and THBS2 in SCC13 and SJG15 cells (Figure 5E). Dox induction of miR-203 in SCC13 and SJG15 cells led to downregulation of LASP1, NUAK1, and SPARC, but not THBS2 (Figure 5G).

We focused on LASP1, NUAK1, and SPARC because of their involvement in metastasis-relevant processes such as cytoskeletal dynamics, energy metabolism, and extracellular matrix remodeling, respectively, and their previously described roles in cancer (Liu et al., 2012; Minn et al., 2005; Traenka et al., 2010). To determine whether or not *LASP1*, *NUAK1*, and *SPARC* are direct miR-203 targets, we cloned the 3′ UTRs into a luciferase reporter construct. The 3′ UTR of *TP63*, a known downstream target of miR-203 (Yi et al., 2008), served as a positive control. Transfection of exogenous miR-203 molecules (mimics) into 293T cells led to specific upregulation of miR-203 levels (Figure 5H). miR-203 mimics repressed the *LASP1*, *NUAK1*, *SPARC*, and *TP63* 3′ UTR reporters, while mutation of the various miR-203 binding sites blocked miR-203-mediated regulation of each 3′ UTR (Figure 5I). Western blotting showed a miR-203-dependent reduction in LASP1, NUAK1, and SPARC protein levels (Figure 5J). Moreover, we found significant negative correlations between RNA sequencing (RNAseq) read counts of miR-203 and each of its three direct targets, but not *TP63*, across 225 HNSCC patients in TCGA (Figure S2A). We thus confirmed LASP1, NUAK1, and SPARC as direct target genes of miR-203 in human HNSCC.

LASP1, NUAK1, and SPARC Are Functionally Important Prometastatic Downstream Effectors of miR-203 that Are Prognostic of Overall Survival in HNSCC

To investigate the functional contribution(s) of LASP1, NUAK1, and SPARC to miR-203-induced inhibition of metastasis, we conducted genetic rescue experiments in vivo (Figure 6A). We engineered control and miR-203 expressing SCC13 cells to stably express all 16 possible combinations of miRNA-insensitive cDNAs (lacking 3′ UTRs) encoding *LASP1*, *NUAK1*, and *SPARC*, and validated correct target gene reconstitution by western blotting (Figure 6B). The levels of exogenous protein expressed were sufficiently high that endogenous NUAK1 and SPARC (Figure 5J) could not be seen on the blots (Figure 6B).

All 16 SCC13 populations were intravenously injected into mice and lung metastasis was monitored for up to 88 days (Figures 6C, S6D, and S6E). Consistent with our earlier findings (Figure 2), SCC13 cells expressing miR-203 together with three

(F) Experimental strategy to quantify cancer cell extravasation across the lung vasculature in vivo. Examples of GFP$^+$ SCC13 cells that have successfully extravasated (out) or are trapped within the lumen of pulmonary blood vessels labeled in red (in) and outlined with a dashed white line are shown. Scale bar represents 10 μm.
(G) Proportion of cells in or out of lung vasculature at 30 hr after intravenous injection. Data are mean % ± SEM, with n = 3 per group. A total of 127–136 cells were counted per mouse.
See also Figure S4.

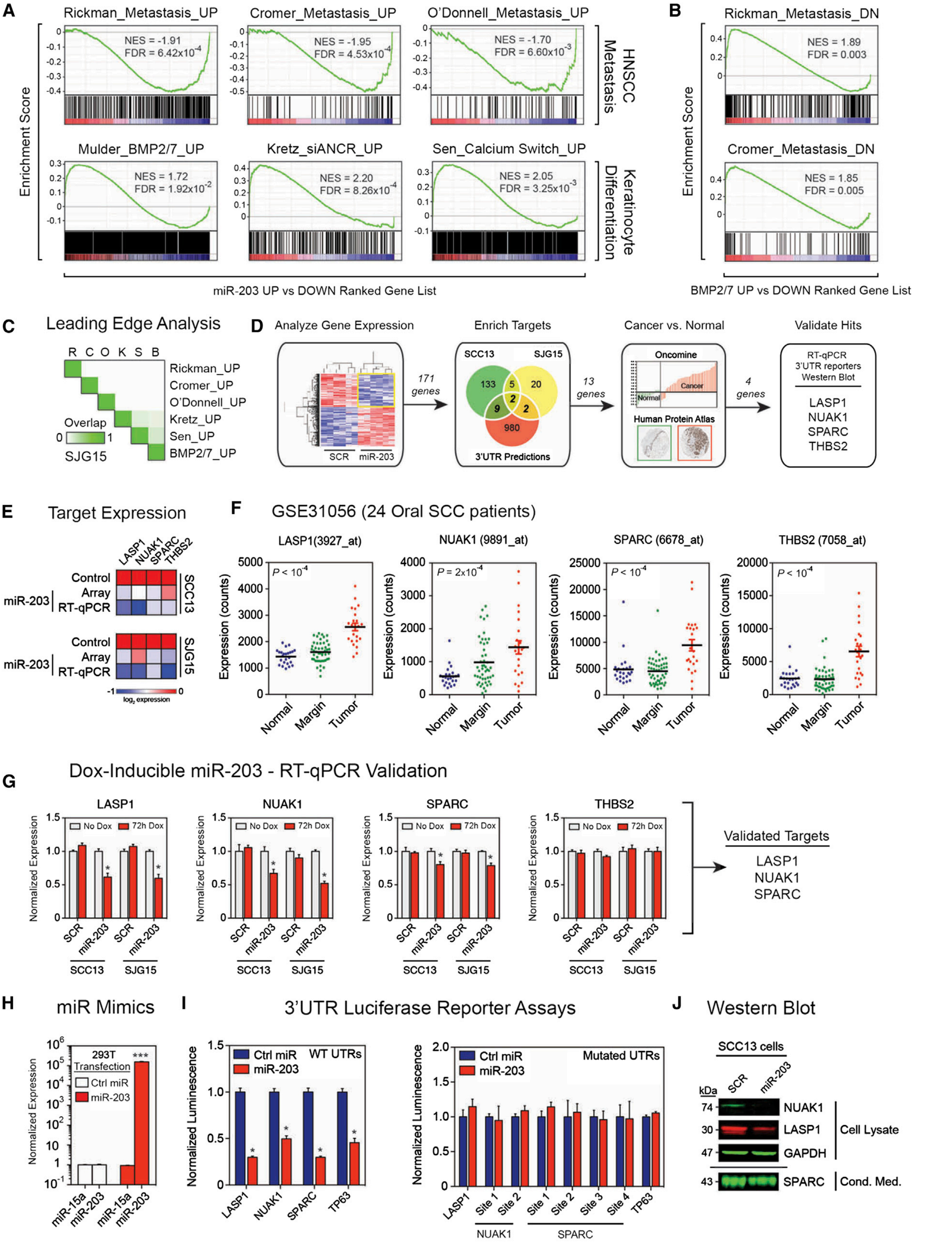

(legend on next page)

empty-vector controls (3xEV) did not colonize the lungs, whereas 3xEV SCC13 SCR cells generated numerous macrometastatic lesions (Figure 6C). Overexpression of SPARC or NUAK1 alone led to a small increase in metastasis of miR-203 cells, whereas overexpression of LASP1 alone caused a pronounced stimulation, which was comparable to overexpressing all three proteins (Figures 6C, S6E, and S6E). Overexpression of LASP1 and/or SPARC, or combined expression of LASP1, SPARC, and NUAK1 did not enhance metastasis of SCR SCC13 cells (Figures 6C–6F, S6D, and S6E). Unexpectedly, when NUAK1 was expressed alone or in combination with SPARC or LASP1, SCR SCC13 cells produced fewer metastases (Figures 6F and S6E).

Individual or combined reintroduction of NUAK1, SPARC, and/or LASP1 was sufficient to bolster the lung metastatic colonization by SCC13 expressing miR-203 (Figure 6C–6F). NUAK1, SPARC, and LASP1 promoted exit from dormancy (Figure 6D) and overt pulmonary colonization, increasing the lung metastatic burden by 7-fold, 10-fold, and 53-fold, respectively (Figures 6E and 6F). Thus, each miR-203 target promotes metastasis in the presence of miR-203, with the effect of LASP1 being most pronounced. In addition, knockdown of LASP1 reduced the clonogenic potential of SCC13 cells in vitro (Figures S6F and S6G), recreating the phenotype observed when miR-203 is expressed in the SCC13 cell line (Figure 2B).

The clinical relevance of the miR-203 target genes was confirmed in patient data. High expression of *LASP1*, *NUAK1*, and *SPARC* correlated with poor prognosis in both the TCGA cohort and a separate publically available HNSCC cohort (Figures 6G and 6H; Cromer et al., 2004).

We conclude that miR-203 inhibits lung metastasis, not by triggering differentiation, but by directly targeting the prometastatic genes *LASP1*, *SPARC*, and *NUAK1*, which are prognostic factors in human HNSCC.

DISCUSSION

Our studies uncover miR-203 as a potent suppressor of key postextravasation events during lung metastasis. Reintroducing miR-203 into already established pulmonary nodules elicits their regression, suggesting the potential for therapeutic modalities aimed at activating miR-203 in cancer cells to treat metastatic HNSCC.

Recent evidence suggests that cancer cells hijack normal stem cell self-renewal signaling pathways to generate and propagate tumors in vivo (Reis et al., 2011). If so, coaxing cancer cells to differentiate may inhibit tumor growth and metastasis. Although miR-203 promotes differentiation of normal epidermal stem cells (Jackson et al., 2013; Yi et al., 2008), our study shows that it does not promote carcinoma differentiation in vivo. Instead, miR-203 controls HNSCC metastasis by targeting a network of prometastatic proteins, including LASP1, SPARC, and NUAK1. Thus, we propose that miR-203 undergoes a context-dependent functional switch from regulating normal differentiation to acting as a differentiation-independent roadblock to metastasis.

It is known that the genetic and epigenetic alterations sustained by host cells during malignant transformation alter the repertoire of mRNA species available for targeting by miRNAs (Lujambio and Lowe, 2012). Thus, miRNAs can perform cellular-context-dependent functions driven by different cohorts of downstream effectors. For example, miR-126 inhibits breast cancer metastasis either by modulating the primary tumor microenvironment (Zhang et al., 2013) or by inhibiting lung metastatic colonization (Png et al., 2012) in mouse or human breast cancer models, respectively. As such, the rewiring of miR-203 function from regulating differentiation to inhibiting metastasis is likely to be explained by changes in target gene selection from differentiation-relevant proteins, such as TP63, to metastasis-promoting factors, such as LASP1, SPARC, and NUAK1.

Figure 5. LASP1, SPARC, and NUAK1 Are Direct Target Genes of miR-203

(A) GSEA plots showing negative enrichment of three signatures of genes upregulated in metastatic HNSCC (top row), and positive enrichment with three signatures of genes upregulated during primary human keratinocyte differentiation in vitro (bottom row), in miR-203-expressing SJG15 cells compared with control. NES, normalized enrichment score; FDR, false-discovery rate q value.

(B) GSEA as in (A) using the ranked gene list (top upregulated to most downregulated) of genes in BMP2/7-treated keratinocytes compared with control.

(C) Leading-edge analysis of genes driving enrichment of metastasis and differentiation signatures in miR-203-expressing SJG15. Signatures on the vertical are abbreviated to first letters only on the horizontal axis. The overlap score (0–1) indicates the degree to which leading-edge genes in each signature are shared with other signatures.

(D) Experimental and bioinformatics strategy to identify clinically relevant miR-203 target genes. Microarray expression profiling of stable miR-203 and control expressing SCC13 and SJG15 cells was followed by enrichment for downregulated direct targets using publically available miRNA target prediction algorithms, and subsequently analysis of microarray and protein expression data sets in Oncomine and the Human Protein Atlas to obtain four candidate target genes for validation.

(E) Heatmap depicting $\log_2$ transformed expression levels of four candidate miR-203 target genes in the microarray experiments, validated by qRT-PCR in control and miR-203-expressing SCC13 and SJG15 cells.

(F) Analysis of GSE31056 for LASP1, NUAK1, SPARC, and THBS2 expression (counts) in normal tissue (24 samples), oral SCC margins (49 samples), and oral SCCs (23 samples); p values were calculated between normal and tumors using the nonparametric Mann-Whitney test.

(G) qRT-PCR for LASP1, NUAK1, SPARC, and THBS2 mRNA expression in SCC13 and SJG15 cells expressing inducible miR-203 or control vector. Cells were either not induced or induced for 3 consecutive days in vitro. Expression normalized to GAPDH. Data are means ± SEM.

(H) qRT-PCR expression of miR-15a and mi-20 in 293T cells transfected with 20 nM negative control miRNA (Ctrl miR) or miR-203 for luciferase reporter assays. Expression was normalized to Ctrl miR. Data are means ± SEM.

(I) Luciferase reporter assays measuring the ability of transfected miR-203 to repress wild-type (left) and mutant (right) 3′ UTR sequences of LASP1, NUAK1, SPARC, and TP63 (positive control). Measurements are the ratios of firefly/renilla luminescence readings relative to control miRNA transfectants. Data are means ± SEM.

(J) Li-Cor western blots of whole-cell lysates (NUAK1, LASP1, and GAPDH) and conditioned medium (SPARC) from control (SCR) and miR-203-expressing SCC13 cell cultures.

*$p < 0.05$, **$p < 0.01$, ***$p < 0.001$ calculated using unpaired Student's t test. See also Figures S5 and S6.

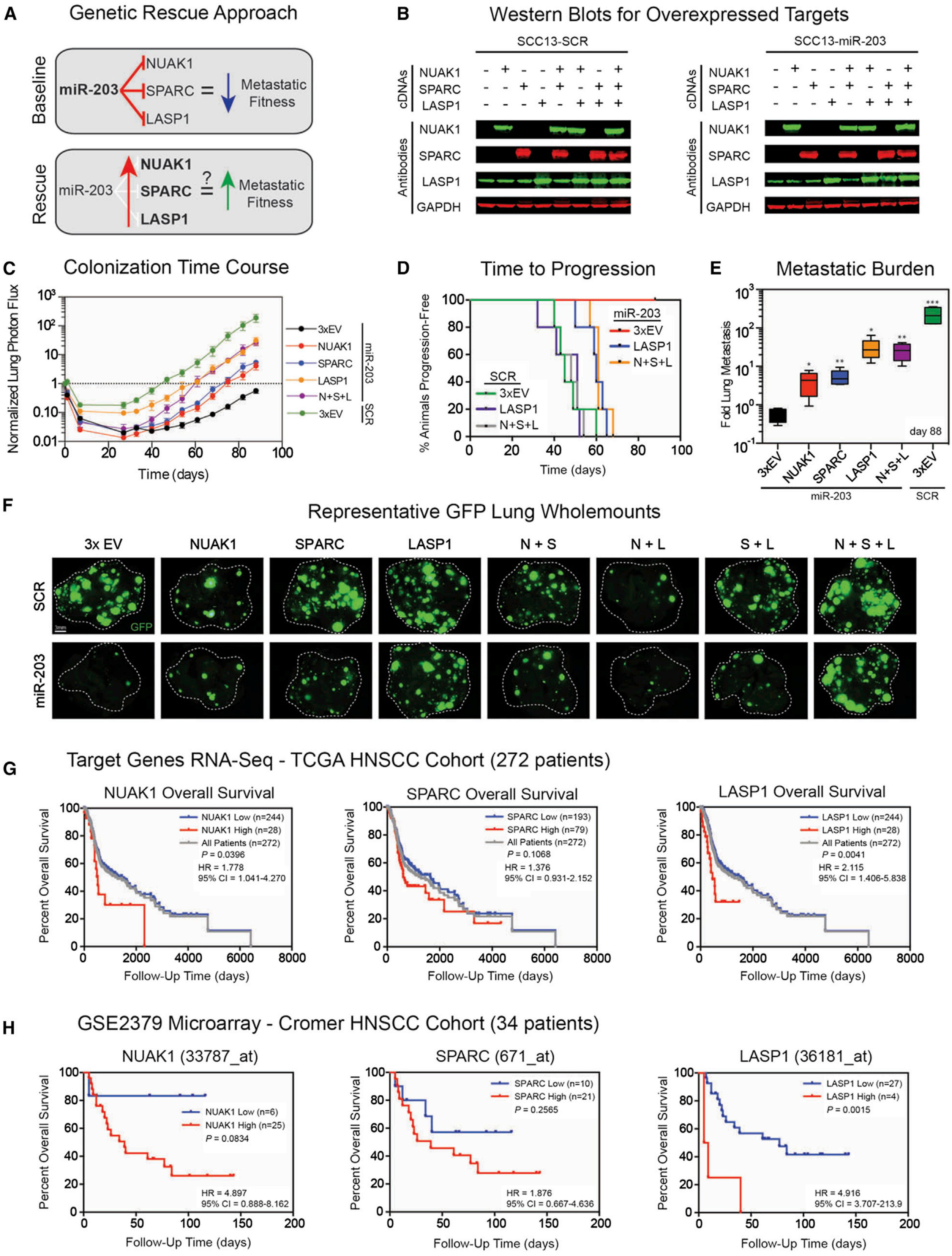

(legend on next page)

Our mechanistic studies identify LASP1, SPARC, and NUAK1 as important direct downstream effectors for miR-203-mediated inhibition of HNSCC metastasis. Prior studies have shown that these proteins promote cancer cell migration and invasion in vitro, promote tumor progression and/or metastasis in vivo, and correlate with poor overall survival in diverse carcinomas (Chang et al., 2012; Chin et al., 2005; Davis et al., 2008; Liu et al., 2012; Minn et al., 2005; Suzuki et al., 2004; Traenka et al., 2010; Viticchiè et al., 2011). We speculate that LASP1, NUAK1, and SPARC promote postextravasation survival and/or engraftment, shorten dormancy, and bolster overt pulmonary colonization by HNSCC cells.

Our data indicate that miR-203 is an upstream governor of several distinct cellular pathways that converge to enforce a poorly metastatic cell state, and highlight the importance of cellular context in determining the effects of a specific miRNA on metastasis. Indeed, miR-203 regulates tumor progression in breast and prostate cancer, but the targets of its action are very distinct among tissue types. In advanced metastatic prostate cancer, miR-203 targets BIRC5 (Saini et al., 2011), whereas in breast cancer metastasis it targets SNAI2 (Ding et al., 2013). This suggests that miR-203 not only functionally switches between tissue homeostasis and cancer progression, as shown here, but also switches targets depending on the cancer type.

In conclusion, our studies highlight miR-203 and its effectors as promising routes for therapeutic intervention in metastatic HNSCC. Unlike most anticancer agents and antimetastatic strategies currently in clinical trials (Valastyan and Weinberg, 2011), miR-203 can antagonize metastasis even after cancer cells have seeded the lung and formed clinically advanced nodules. In light of the development of targeted strategies to deliver nucleic acids into tumor cells (Davis et al., 2008), we envision the potential value of using miR-203 mimetics to alleviate otherwise therapeutically intractable metastatic HNSCC.

EXPERIMENTAL PROCEDURES

Cell Lines

SCC13, SCC25, SJG cell lines, and normal keratinocytes were cultured in FAD medium under standard conditions. Work with human material was either carried out in compliance with the UK Human Tissue Act (2004) and approved by the National Research Ethics Service (08/H0306/30) or according to the recommendations of the local ethics committee and the German Medical Council for diagnostic tissue used in research.

Animal Studies

Tongue xenografting and experimental lung metastasis experiments were performed as previously described (Goldie et al., 2012). Animal studies were subject to Cancer Research UK and King's College London ethical review and performed in accordance with an approved UK Government Home Office license. A dox-rich diet (Harlan) was used to induce miR-203 expression in vivo. Bioluminescent imaging was conducted using a Xenogen IVIS 200 system (Perkin Elmer).

qRT-PCR, Microarrays, and GEO Data Sets

RNA was isolated using a miRNeasy Mini Kit (QIAGEN), and qRT-PCR was performed using TaqMan probes (Life Technologies) and SYBR Green primers. Genome-wide expression analysis was carried out on Illumina Human HT12 version 4 arrays. Microarray data sets GSE31056 and GSE2379 were downloaded from NCBI GEO. TCGA data were downloaded from https://tcga-data.nci.nih.gov/tcga/ and http://www.broadinstitute.org/tcga/home.

Statistical Analysis

An unpaired two-tailed Student's t test (in vitro experiments) or a nonparametric Mann-Whitney test (in vivo mouse experiments) was used for comparisons, with $p < 0.05$ considered significant. A log rank (Mantel-Cox) test was used to compare survival curves and compute hazard ratios, and Fisher's exact test used to compare human cohorts on the basis of clinical characteristics.

ACCESSION NUMBERS

The NCBI Gene Expression Omnibus accession number for the gene expression data reported in this paper is GSE47028.

SUPPLEMENTAL INFORMATION

Supplemental Information includes Supplemental Experimental Procedures, six figures, and one table and can be found with this article online at http://dx.doi.org/10.1016/j.celrep.2014.08.062.

AUTHOR CONTRIBUTIONS

N.B. and F.M.W. conceived the project. N.B., S.W., and F.M.W. wrote the manuscript. N.B., S.W., and A.M. performed experiments. S.J.G. and S.R.Q. provided reagents.

Figure 6. LASP1, SPARC, and NUAK1 Can Overcome miR-203 Metastasis Suppression

(A) Schematic model for miR-203-driven suppression of lung metastatic fitness in SCC, and hypothesis for combinatorial genetic rescue by overexpression of miR-203-insensitive LASP1, NUAK1, and SPARC in miR-203-expressing SCC13 cells.

(B) Li-Cor western blots of 16 different whole-cell lysates from control (SCR) and miR-203-expressing SCC13 cells also overexpressing single, double, or triple combinations of LASP1, NUAK1, and SPARC cDNAs. GAPDH was used as a loading control.

(C) Time course of experimental lung metastasis generated by control (SCR) SCC13 cells expressing 3xEV and miR-203 SCC13 cells expressing 3xEV, NUAK1, SPARC, or LASP1, or NUAK1, SPARC, and LASP1 together (N+S+L) over 88 days (n = 5 per group). Log_{10} y axis; data are means ± SEM with n = 4–5 per group. Data were selected from the full set of combinations presented in Figure S6D.

(D) Kaplan-Meier plots for time to progression of lung metastasis of control and miR-203 SCC13 cells expressing 3xEV, LASP1 alone, or all three target gene cDNAs; p values were calculated using the log rank Mantel-Cox test; n = 4–5 per group.

(E) Lung metastasis formed by 3xEV, NUAK1, SPARC, or LASP1, or NUAK1, SPARC, and LASP1 together (N+S+L) of miR203 SCC13 cell lines and 3xEV in the SCR SCC13 cell line, analyzed at day 88. Whiskers indicate min/max and the horizontal bar is median, with n = 4–5 per group. Log_{10} y axis. *$p < 0.05$ and **$p < 0.01$ were calculated using the nonparametric Mann-Whitney test in comparison with miR-203 3xEV.

(F) Representative whole-mount ex vivo GFP fluorescence microscopy images of lung metastases formed by all 16 combinations of SCC13 cell lines at day 88. Scale bar represents 3 mm.

(G) Kaplan-Meier plots for overall survival of 272 HNSCC patients from the TCGA cohort partitioned on the basis of LASP1, NUAK1, and SPARC RNAseq read counts; p values were calculated using the log rank Mantel-Cox test.

(H) LASP1, NUAK1, and SPARC mRNA expression was used to partition the GSE2379 cohort of 34 HNSCC patients; p values were calculated using the log rank Mantel-Cox test.

ACKNOWLEDGMENTS

We thank Scott Valastyan, Alasdair Russell, Hironobu Fujiwara, Scott Lyons, Klaas Mulder, Carles Escriu, Andrew Sims, and the F.M.W. laboratory for helpful discussions and materials. We are grateful for experimental support from CRUK-CRI core facilities (Histopathology, Biological Resources, Genomics and Bioinformatics, Light Microscopy, Flow Cytometry, and the Equipment Park). N.B. was funded by a Gates Cambridge Scholarship and a Williams College Herchel Smith Fellowship. This research was funded by grants to F.M.W. from the EU Seventh Framework Programme (OptiStem), Medical Research Council (G1100073), Wellcome Trust (096540/Z/11/Z), and Cancer Research UK.

Received: September 19, 2013
Revised: August 5, 2014
Accepted: August 25, 2014
Published: October 2, 2014

REFERENCES

Agrawal, N., Frederick, M.J., Pickering, C.R., Bettegowda, C., Chang, K., Li, R.J., Fakhry, C., Xie, T.X., Zhang, J., Wang, J., et al. (2011). Exome sequencing of head and neck squamous cell carcinoma reveals inactivating mutations in NOTCH1. Science *333*, 1154–1157.

Aqeilan, R.I., Calin, G.A., and Croce, C.M. (2010). miR-15a and miR-16-1 in cancer: discovery, function and future perspectives. Cell Death Differ. *17*, 215–220.

Argiris, A., Karamouzis, M.V., Raben, D., and Ferris, R.L. (2008). Head and neck cancer. Lancet *371*, 1695–1709.

Arwert, E.N., Hoste, E., and Watt, F.M. (2012). Epithelial stem cells, wound healing and cancer. Nat. Rev. Cancer *12*, 170–180.

Blanpain, C., and Fuchs, E. (2006). Epidermal stem cells of the skin. Annu. Rev. Cell Dev. Biol. *22*, 339–373.

Chakrabarti, R., Hwang, J., Andres Blanco, M., Wei, Y., Lukačišin, M., Romano, R.A., Smalley, K., Liu, S., Yang, Q., Ibrahim, T., et al. (2012). Elf5 inhibits the epithelial-mesenchymal transition in mammary gland development and breast cancer metastasis by transcriptionally repressing Snail2. Nat. Cell Biol. *14*, 1212–1222.

Chang, X.Z., Yu, J., Liu, H.Y., Dong, R.H., and Cao, X.C. (2012). ARK5 is associated with the invasive and metastatic potential of human breast cancer cells. J. Cancer Res. Clin. Oncol. *138*, 247–254.

Chin, D., Boyle, G.M., Williams, R.M., Ferguson, K., Pandeya, N., Pedley, J., Campbell, C.M., Theile, D.R., Parsons, P.G., and Coman, W.B. (2005). Novel markers for poor prognosis in head and neck cancer. Int. J. Cancer *113*, 789–797.

Cromer, A., Carles, A., Millon, R., Ganguli, G., Chalmel, F., Lemaire, F., Young, J., Dembélé, D., Thibault, C., Muller, D., et al. (2004). Identification of genes associated with tumorigenesis and metastatic potential of hypopharyngeal cancer by microarray analysis. Oncogene *23*, 2484–2498.

Davis, M.E., Chen, Z.G., and Shin, D.M. (2008). Nanoparticle therapeutics: an emerging treatment modality for cancer. Nat. Rev. Drug Discov. *7*, 771–782.

Ding, X., Park, S.I., McCauley, L.K., and Wang, C.-Y. (2013). Signaling between transforming growth factor β (TGF-β) and transcription factor SNAI2 represses expression of microRNA miR-203 to promote epithelial-mesenchymal transition and tumor metastasis. J. Biol. Chem. *288*, 10241–10253.

Flores, E.R., Sengupta, S., Miller, J.B., Newman, J.J., Bronson, R., Crowley, D., Yang, A., McKeon, F., and Jacks, T. (2005). Tumor predisposition in mice mutant for p63 and p73: evidence for broader tumor suppressor functions for the p53 family. Cancer Cell *7*, 363–373.

Goldie, S.J., Mulder, K.W., Tan, D.W.M., Lyons, S.K., Sims, A.H., and Watt, F.M. (2012). FRMD4A upregulation in human squamous cell carcinoma promotes tumor growth and metastasis and is associated with poor prognosis. Cancer Res. *72*, 3424–3436.

Hüsemann, Y., Geigl, J.B., Schubert, F., Musiani, P., Meyer, M., Burghart, E., Forni, G., Eils, R., Fehm, T., Riethmüller, G., and Klein, C.A. (2008). Systemic spread is an early step in breast cancer. Cancer Cell *13*, 58–68.

Jackson, S.J., Zhang, Z., Feng, D., Flagg, M., O'Loughlin, E., Wang, D., Stokes, N., Fuchs, E., and Yi, R. (2013). Rapid and widespread suppression of self-renewal by microRNA-203 during epidermal differentiation. Development *140*, 1882–1891.

Kim, J.B., Urban, K., Cochran, E., Lee, S., Ang, A., Rice, B., Bata, A., Campbell, K., Coffee, R., Gorodinsky, A., et al. (2010). Non-invasive detection of a small number of bioluminescent cancer cells in vivo. PLoS ONE *5*, e9364.

Kretz, M., Webster, D.E., Flockhart, R.J., Lee, C.S., Zehnder, A., Lopez-Pajares, V., Qu, K., Zheng, G.X., Chow, J., Kim, G.E., et al. (2012). Suppression of progenitor differentiation requires the long noncoding RNA ANCR. Genes Dev. *26*, 338–343.

Lapouge, G., Beck, B., Nassar, D., Dubois, C., Dekoninck, S., and Blanpain, C. (2012). Skin squamous cell carcinoma propagating cells increase with tumour progression and invasiveness. EMBO J. *31*, 4563–4575.

Leemans, C.R., Braakhuis, B.J.M., and Brakenhoff, R.H. (2011). The molecular biology of head and neck cancer. Nat. Rev. Cancer *11*, 9–22.

Liu, L., Ulbrich, J., Müller, J., Wüstefeld, T., Aeberhard, L., Kress, T.R., Muthalagu, N., Rycak, L., Rudalska, R., Moll, R., et al. (2012). Deregulated MYC expression induces dependence upon AMPK-related kinase 5. Nature *483*, 608–612.

Lujambio, A., and Lowe, S.W. (2012). The microcosmos of cancer. Nature *482*, 347–355.

Martello, G., Rosato, A., Ferrari, F., Manfrin, A., Cordenonsi, M., Dupont, S., Enzo, E., Guzzardo, V., Rondina, M., Spruce, T., et al. (2010). A MicroRNA targeting dicer for metastasis control. Cell *141*, 1195–1207.

Minn, A.J., Gupta, G.P., Siegel, P.M., Bos, P.D., Shu, W., Giri, D.D., Viale, A., Olshen, A.B., Gerald, W.L., and Massagué, J. (2005). Genes that mediate breast cancer metastasis to lung. Nature *436*, 518–524.

Mulder, K.W., Wang, X., Escriu, C., Ito, Y., Schwarz, R.F., Gillis, J., Sirokmány, G., Donati, G., Uribe-Lewis, S., Pavlidis, P., et al. (2012). Diverse epigenetic strategies interact to control epidermal differentiation. Nat. Cell Biol. *14*, 753–763.

Nguyen, B.C., Lefort, K., Mandinova, A., Antonini, D., Devgan, V., Della Gatta, G., Koster, M.I., Zhang, Z., Wang, J., Tommasi di Vignano, A., et al. (2006). Cross-regulation between Notch and p63 in keratinocyte commitment to differentiation. Genes Dev. *20*, 1028–1042.

Nicolas, M., Wolfer, A., Raj, K., Kummer, J.A., Mill, P., van Noort, M., Hui, C.C., Clevers, H., Dotto, G.P., and Radtke, F. (2003). Notch1 functions as a tumor suppressor in mouse skin. Nat. Genet. *33*, 416–421.

O'Donnell, R.K., Kupferman, M., Wei, S.J., Singhal, S., Weber, R., O'Malley, B., Cheng, Y., Putt, M., Feldman, M., Ziober, B., and Muschel, R.J. (2005). Gene expression signature predicts lymphatic metastasis in squamous cell carcinoma of the oral cavity. Oncogene *24*, 1244–1251.

Pantel, K., and Brakenhoff, R.H. (2004). Dissecting the metastatic cascade. Nat. Rev. Cancer *4*, 448–456.

Pardal, R., Clarke, M.F., and Morrison, S.J. (2003). Applying the principles of stem-cell biology to cancer. Nat. Rev. Cancer *3*, 895–902.

Png, K.J., Halberg, N., Yoshida, M., and Tavazoie, S.F. (2012). A microRNA regulon that mediates endothelial recruitment and metastasis by cancer cells. Nature *481*, 190–194.

Reis, P.P., Waldron, L., Perez-Ordonez, B., Pintilie, M., Galloni, N.N., Xuan, Y., Cervigne, N.K., Warner, G.C., Makitie, A.A., Simpson, C., et al. (2011). A gene signature in histologically normal surgical margins is predictive of oral carcinoma recurrence. BMC Cancer *11*, 437.

Rheinwald, J.G., and Beckett, M.A. (1981). Tumorigenic keratinocyte lines requiring anchorage and fibroblast support cultured from human squamous cell carcinomas. Cancer Res. *41*, 1657–1663.

Rhodes, D.R., Kalyana-Sundaram, S., Mahavisno, V., Varambally, R., Yu, J., Briggs, B.B., Barrette, T.R., Anstet, M.J., Kincead-Beal, C., Kulkarni, P.,

et al. (2007). Oncomine 3.0: genes, pathways, and networks in a collection of 18,000 cancer gene expression profiles. Neoplasia *9*, 166–180.

Rickman, D.S., Millon, R., De Reynies, A., Thomas, E., Wasylyk, C., Muller, D., Abecassis, J., and Wasylyk, B. (2008). Prediction of future metastasis and molecular characterization of head and neck squamous-cell carcinoma based on transcriptome and genome analysis by microarrays. Oncogene *27*, 6607–6622.

Saini, S., Majid, S., Yamamura, S., Tabatabai, L., Suh, S.O., Shahryari, V., Chen, Y., Deng, G., Tanaka, Y., and Dahiya, R. (2011). Regulatory role of mir-203 in prostate cancer progression and metastasis. Clin. Cancer Res. *17*, 5287–5298.

Sen, G.L., Reuter, J.A., Webster, D.E., Zhu, L., and Khavari, P.A. (2010). DNMT1 maintains progenitor function in self-renewing somatic tissue. Nature *463*, 563–567.

Sonkoly, E., Lovén, J., Xu, N., Meisgen, F., Wei, T., Brodin, P., Jaks, V., Kasper, M., Shimokawa, T., Harada, M., et al. (2012). MicroRNA-203 functions as a tumor suppressor in basal cell carcinoma. Oncogenesis *1*, e3.

Stransky, N., Egloff, A.M., Tward, A.D., Kostic, A.D., Cibulskis, K., Sivachenko, A., Kryukov, G.V., Lawrence, M.S., Sougnez, C., McKenna, A., et al. (2011). The mutational landscape of head and neck squamous cell carcinoma. Science *333*, 1157–1160.

Suzuki, A., Lu, J., Kusakai, G., Kishimoto, A., Ogura, T., and Esumi, H. (2004). ARK5 is a tumor invasion-associated factor downstream of Akt signaling. Mol. Cell. Biol. *24*, 3526–3535.

Taube, J.H., Malouf, G.G., Lu, E., Sphyris, N., Vijay, V., Ramachandran, P.P., Ueno, K.R., Gaur, S., Nicoloso, M.S., Rossi, S., et al. (2013). Epigenetic silencing of microRNA-203 is required for EMT and cancer stem cell properties. Sci Rep *3*, 2687.

Tiffen, J.C., Bailey, C.G., Ng, C., Rasko, J.E.J., and Holst, J. (2010). Luciferase expression and bioluminescence does not affect tumor cell growth in vitro or in vivo. Mol. Cancer *9*, 299.

Traenka, C., Remke, M., Korshunov, A., Bender, S., Hielscher, T., Northcott, P.A., Witt, H., Ryzhova, M., Felsberg, J., Benner, A., et al. (2010). Role of LIM and SH3 protein 1 (LASP1) in the metastatic dissemination of medulloblastoma. Cancer Res. *70*, 8003–8014.

Uhlen, M., Oksvold, P., Fagerberg, L., Lundberg, E., Jonasson, K., Forsberg, M., Zwahlen, M., Kampf, C., Wester, K., Hober, S., et al. (2010). Towards a knowledge-based Human Protein Atlas. Nat. Biotechnol. *28*, 1248–1250.

Valastyan, S., and Weinberg, R.A. (2011). Tumor metastasis: molecular insights and evolving paradigms. Cell *147*, 275–292.

Valastyan, S., Benaich, N., Chang, A., Reinhardt, F., and Weinberg, R.A. (2009a). Concomitant suppression of three target genes can explain the impact of a microRNA on metastasis. Genes Dev. *23*, 2592–2597.

Valastyan, S., Reinhardt, F., Benaich, N., Calogrias, D., Szász, A.M., Wang, Z.C., Brock, J.E., Richardson, A.L., and Weinberg, R.A. (2009b). A pleiotropically acting microRNA, miR-31, inhibits breast cancer metastasis. Cell *137*, 1032–1046.

Valastyan, S., Chang, A., Benaich, N., Reinhardt, F., and Weinberg, R.A. (2010). Concurrent suppression of integrin alpha5, radixin, and RhoA phenocopies the effects of miR-31 on metastasis. Cancer Res. *70*, 5147–5154.

Valastyan, S., Chang, A., Benaich, N., Reinhardt, F., and Weinberg, R.A. (2011). Activation of miR-31 function in already-established metastases elicits metastatic regression. Genes Dev. *25*, 646–659.

Viticchiè, G., Lena, A.M., Latina, A., Formosa, A., Gregersen, L.H., Lund, A.H., Bernardini, S., Mauriello, A., Miano, R., Spagnoli, L.G., et al. (2011). MiR-203 controls proliferation, migration and invasive potential of prostate cancer cell lines. Cell Cycle *10*, 1121–1131.

Yi, R., Poy, M.N., Stoffel, M., and Fuchs, E. (2008). A skin microRNA promotes differentiation by repressing 'stemness'. Nature *452*, 225–229.

Zhang, Y., Yang, P., Sun, T., Li, D., Xu, X., Rui, Y., Li, C., Chong, M., Ibrahim, T., Mercatali, L., et al. (2013). miR-126 and miR-126* repress recruitment of mesenchymal stem cells and inflammatory monocytes to inhibit breast cancer metastasis. Nat. Cell Biol. *15*, 284–294.

Cell Reports, Volume *9*

Supplemental Information

Rewiring of an Epithelial Differentiation Factor, miR-203, to Inhibit Human Squamous Cell Carcinoma Metastasis

Nathan Benaich, Samuel Woodhouse, Stephen J. Goldie, Ajay Mishra, Sven R. Quist, and Fiona M. Watt

SUPPLEMENTAL INFORMATION

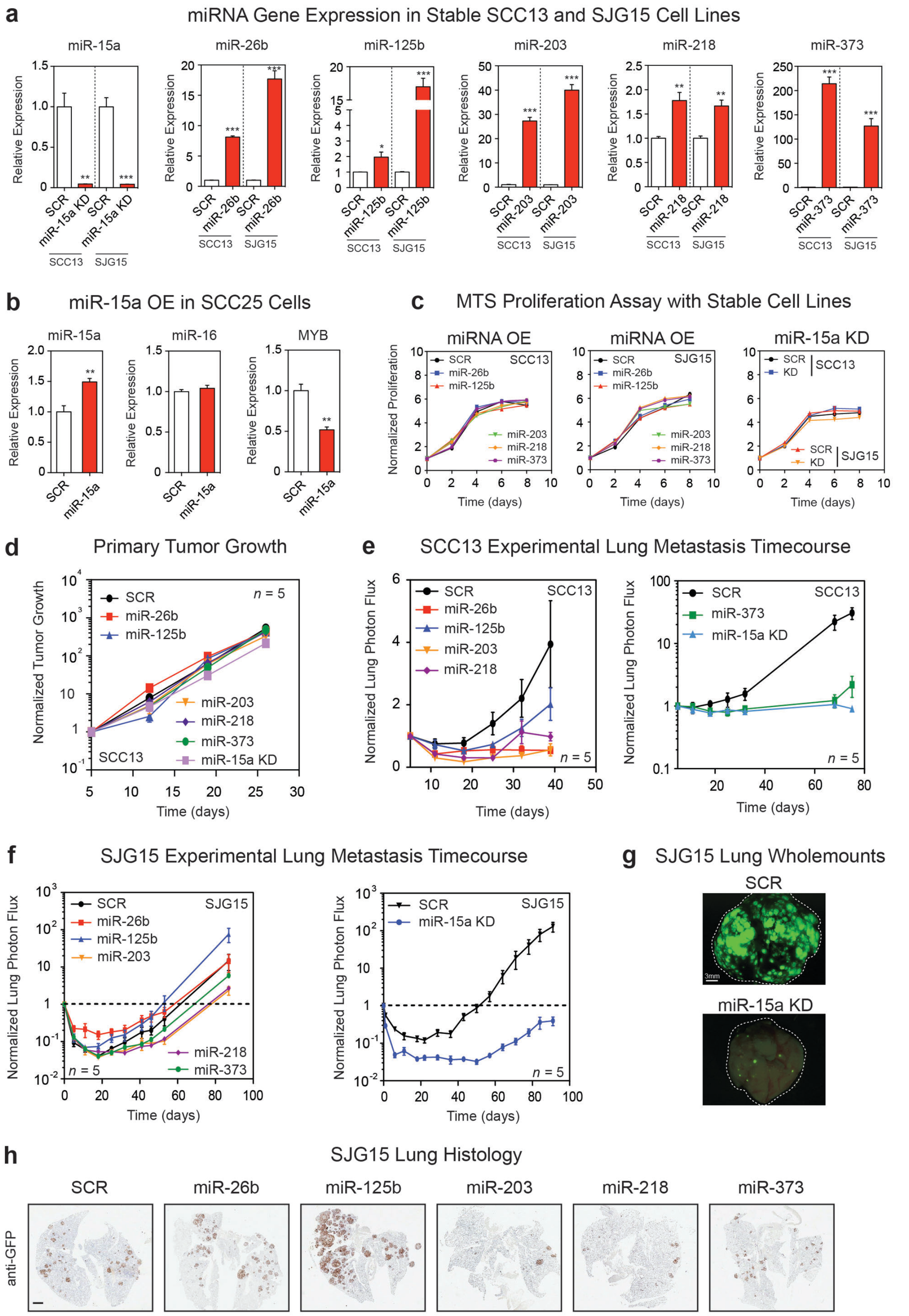

Figure S1. Stable SCC13 and SJG15 cell line validation and additional *in vivo* data panels. Related to Figure 1

(a) qRT-PCR was used to assay expression levels of mature miR-15a, miR-26b, miR-125b, miR-203, miR-218, and miR-373 in SCC13 and SJG15 cells stably infected with either overexpression or knockdown (KD) vectors. Expression was normalized to SCR in each case. Data are mean ± SEM.

(b) miR-15a overexpression, but not miR-16, in SCC25 cells was verified by qRT-PCR, as well as its published target gene, *MYB*. Data are mean ± SEM.

(c) MTS assay measuring *in vitro* growth kinetics of SCC13 and SJG15 cells from panel (a). Each data point was normalized to the day 0 value within each group of cells. Data are mean ± SEM.

(d) Time course of primary tongue tumor growth starting with 10^5 SCC13 cells. Total lung photon flux was normalized to day 5 for each mouse and expressed as a relative fold change. Log_{10} y-axis, data are mean ± SEM with *n*=5 per group.

(e) Time course of SCC13 experimental lung metastasis using cells in panel (a). Data are normalized to bioluminescence at input and presented as mean ± SEM with *n*=4-5 per group.

(f) Same as (e) except using SJG15 cells.

(g) *Ex vivo* fluorescence lung wholemounts of mice tail-vein injected with control or miR-15a KD SJG15 cells at day 91. Scale bar, 3 mm.

(h) Matched representative anti-GFP immunohistochemistry of lungs injected with SJG15 after 87 days after tail-vein injection of indicated SJG15 cells. Scale bar, 50 μm.

**P* < 0.05; ***P* < 0.01; ****P* < 0.001 calculated using a non-parametric Mann-Whitney test (mouse experiments) or a two-tailed Student's t-test (qRT-PCR data).

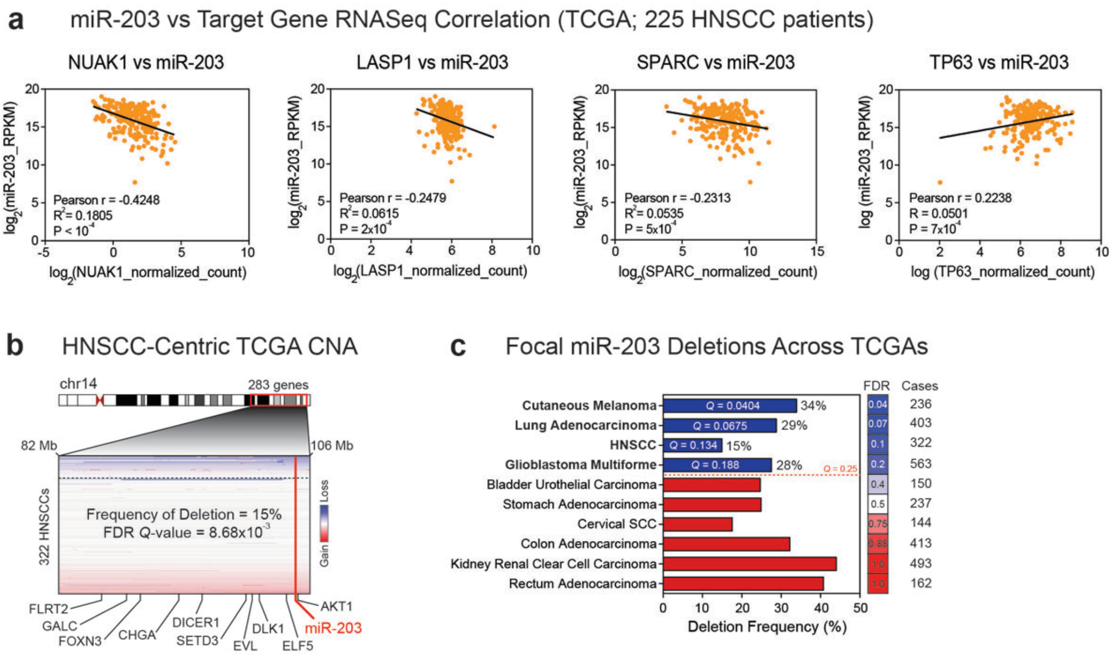

Figure S2. RNASeq correlations between miR-203 and target genes in HNSCC TCGA cohort and CNA analysis. Related to Figure 2

(a) miRNASeq (RPKM) and RNASeq (normalized counts) paired values for 225 HNSCC TCGA primary tumors were plotted against one another and correlations calculated using a Pearson's test. Note negative correlation between miR-203 and NUAK1, LASP1, and SPARC, but absence of such correlation with a previously reported target, TP63.

(b) Schematic representation of a 24 Mb region on human chromosome 14 incorporating miR-203 that is significantly focally deleted in 15% of HNSCC cases in the 322 TCGA patient cohort. Indicated genes are an example subset. Blue indicates deletion and red indicates amplification. False discovery rate Q-value (<0.25 cutoff) calculated using GISTIC statistics.

(c) A miR-203-centric GISTIC analysis of TCGAs cohorts reveals significant deletion of miR-203 in multiple tumor types (significant in red; non-significant in blue, using *Q*<0.25 as a cutoff).

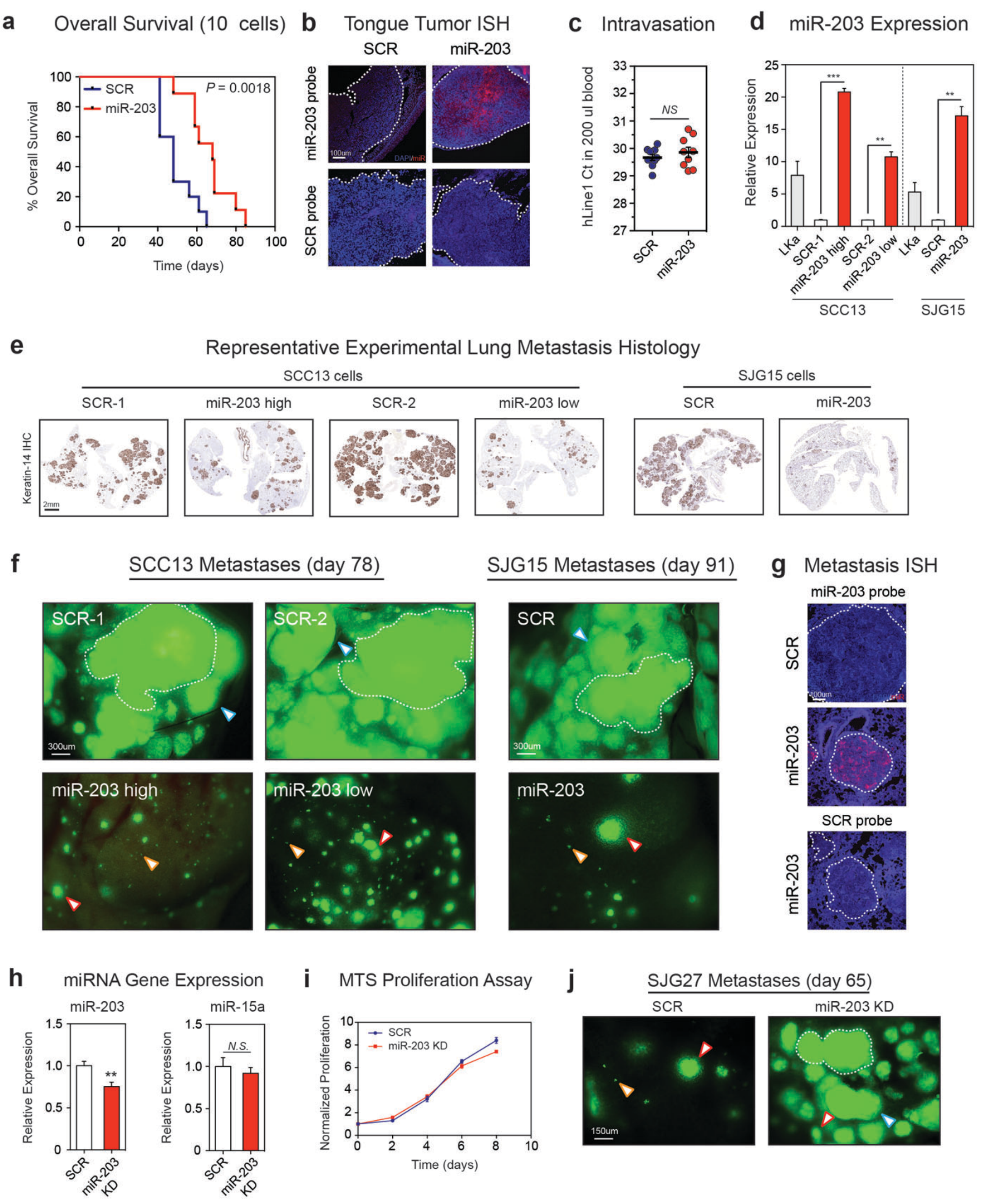

Figure S3. miR-203 primarily regulates lung metastatic colonization *in vivo*. Related to Figure 2 and Figure 3

(a) Kaplan-Meier overall survival curve for animals tongue xenografted with 10^4 control or miR-203 expressing SCC13 cells (n=10 per group).

(b) Primary tongue tumors formed by control (SCR) and miR-203 expressing SCC13 cells stained with miR-203 or scrambled LNA probes (Exiqon). miR-203 signal in red and DAPI nuclei in blue. Scale bar is 100 µm. Dotted lines outline metastases.

(c) *in vivo* tumor cell intravasation was measured by qRT-PCR for human-specific *AluJ* repeat elements present in circulating tumor cells within 200 ul of whole mouse

blood. *n*=10 (SCR) and *n*=9 (miR-203). *P*-values calculated using a non-parametric Mann-Whitney test.

(d) qRT-PCR expression of miR-203 high and miR-203 low SCC13 cells, SJG15 cells expressing miR-203, and normal human primary lip keratinocytes (LKa). Expression was normalized to SCR in each case. Data are mean ± SEM.

(e) Representative keratin-14 immunohistochemistry of experimental lung metastases formed by SCC13 cells expressing high or low miR-203, as well as primary SJG15 cells expressing miR-203. Sections are of matched lungs from fluorescence images in Figure 2j. Scale bar is 2mm.

(f) High magnification GFP fluorescence microscopy images of control and miR-203 experimental lung metastases formed by SCC13 or SJG15 cells *in vivo* after 78 or 91 days, respectively. Dashed lines outline massive metastatic areas observed only in control animals. Blue arrowheads point to macrometastases; red arrowheads indicate small macrometastases or micrometastases; orange arrowheads show clusters of single cells. Scale bars are 300 μm.

(g) Experimental lung metastases formed by control (SCR) and miR-203 expressing SCC13 cells were stained with miR-203 or scrambled LNA probes (Exiqon). Note presence of miR-203 signal in metastases formed by miR-203 expressing cells, but not control cells. Scale bar and dotted lines are the same as in panel (e).

(h) qRT-PCR gene expression measuring miR-203 and miR-15a (control) levels in SJG27 cells expressing scrambled control hairpin (SCR) or miR-203 knockdown vector (KD). Expression was normalized to SCR in each case. Data are mean ± SEM.

(i) MTS assay measuring *in vitro* growth kinetics of control and miR-203 knockdown SJG27 cells. Each data point was normalized to the day 0 value within each group of cells. Data are mean ± SEM. *P*-values calculated using a two-tailed Student's t-test.

(j) High magnification GFP fluorescence microscopy images of SJG27 control and miR-203 KD experimental lung metastases after 65 days. Dashed lines outline massive metastatic areas observed only in control animals. Blue arrowheads point to macrometastases; red arrowheads indicate small macrometastases or micrometastases; orange arrowheads show clusters of single cells. Scale bars are 150 μm.

*$P < 0.05$; **$P < 0.01$; ***$P < 0.001$ calculated using a two-tailed Student's t-test for panels (d) and (h).

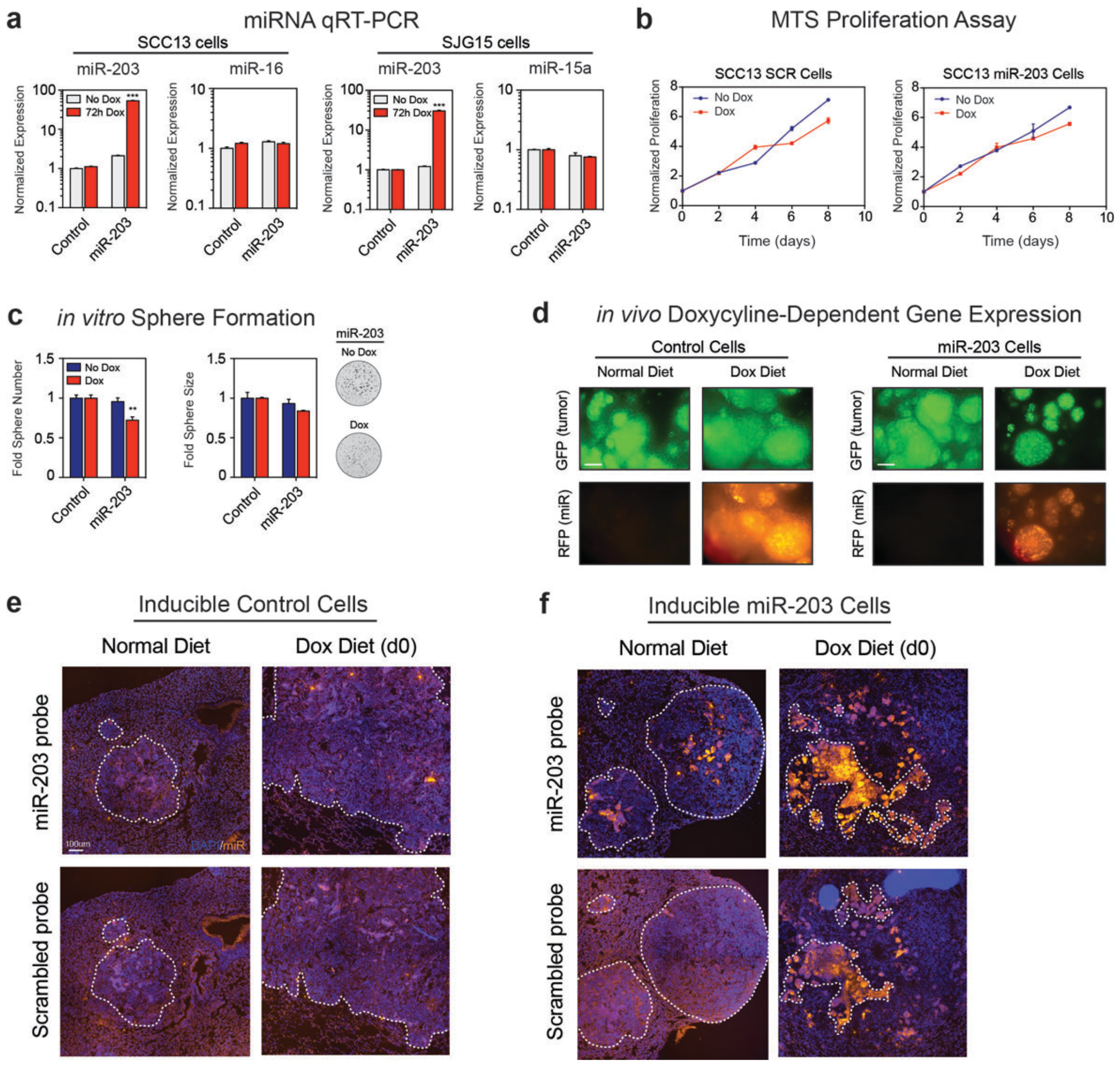

Figure S4. Validation experiments for doxycycline-inducible miR-203 vector system. Related to Figure 4

(a) qRT-PCR expression analysis of miR-203 and either miR-16 or miR-15a (control miRNAs) in control and miR-203 inducible SCC13 and SJG15 cells after *in vitro* treatment with dox for 72h. Note dox-dependent induction of miR-203 but not irrelevant miRNAs, miR-15a or miR-16. Data are mean ± SEM with a $\log_{10}$ y-axis

(b) MTS assay measuring *in vitro* growth kinetics of SCC13 in panel (b) either non-induced or induced with dox starting 3 days prior to the experiment. Each data point was normalized to the day 0 value within each group of cells. Data are mean ± SEM.

(c) *in vitro* anchorage-independent soft agar assay using SCR and miR-203 inducible SCC13 cells either not induced or induced continuously with dox. Fold changes in sphere number and size (5000 cells seeded/well) are normalized to respective non-dox

treated groups. Data are presented mean ± SEM. Representative GFP scans of wells with non-induced or induced miR-203 spheres are shown on the right.

(d) *ex vivo* GFP (all tumor cells) and RFP (only induced SCR or miR-203) fluorescence microscopy images of lung metastases formed by control and miR-203 inducible SCC13 cells in animals fed with either a normal diet (no dox) or dox-rich diet starting at 10 days post-injection. Images are at 8x magnification, scale bar is 500 μm

(e) and (f) Experimental lung metastases formed by inducible control (SCR) (e) and miR-203 expressing SCC13 cells (f) in mice fed with normal diet (non-induced) or dox-rich diet starting at day 0 of the experiment were stained with miR-203 or scrambled LNA probes (Exiqon). miR-203 signal is orange and DAPI nuclei in blue. Scale bar is 100 μm and lung metastases outlined using dotted lines. Note the absence of miR-203 signal in non-induced and induced control metastases in panel (e), and the strong miR-203 signal present in dox-induced miR-203 lung metastases in panel (f).

*$P < 0.05$; **$P < 0.01$; ***$P < 0.001$ calculated using a two-tailed Student's t-test.

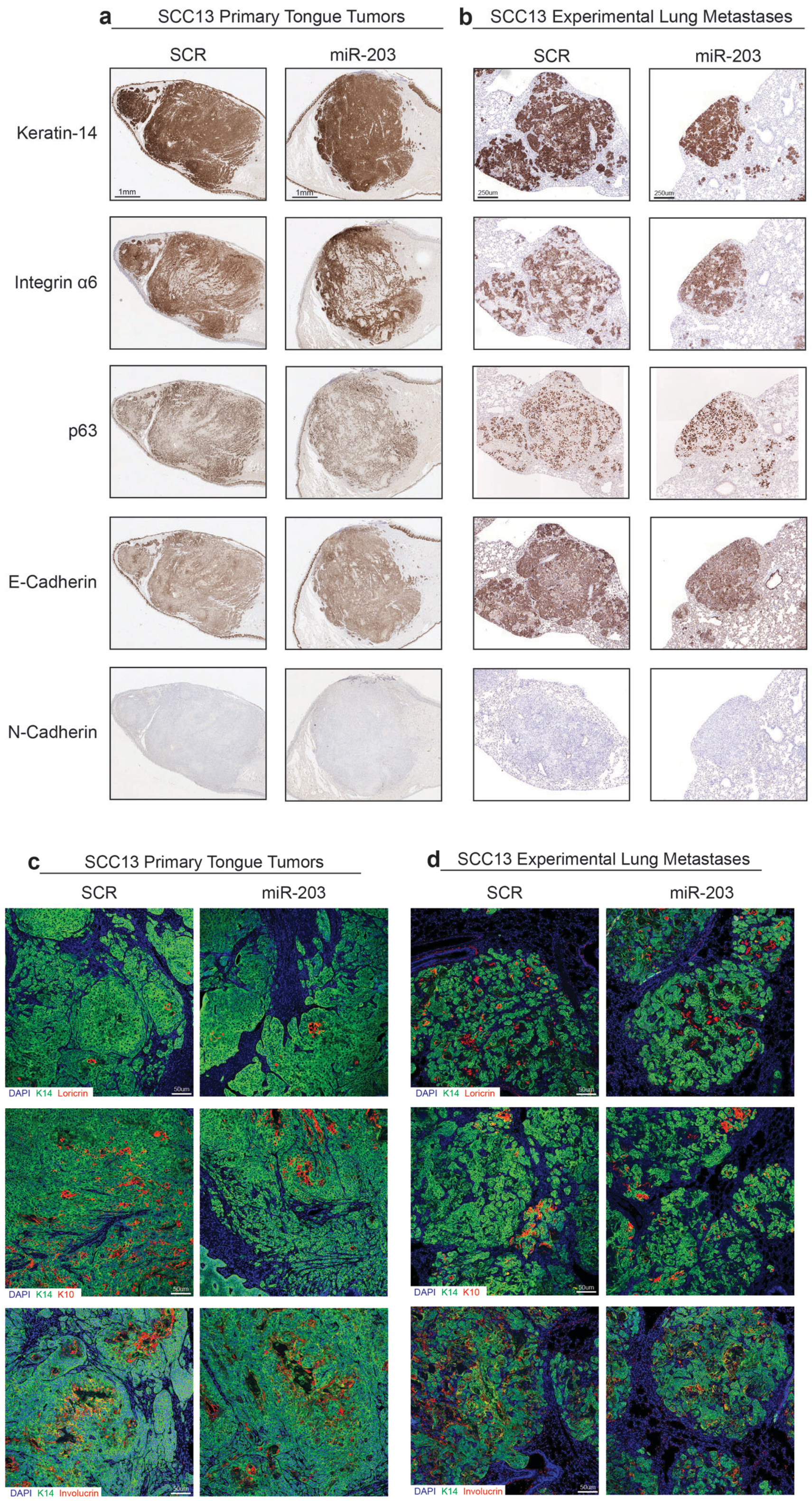

Figure S5. Analysis of epithelial markers and differentiation status in SCC13 primary tongue tumors and experimental lung metastases. Related to Figure 5.

(a-d) Primary tongue xenograft tumors (a, c) and experimental lung metastases (b, d) generated by SCC13 cells expressing control (SCR) or miR-203 were immunolabelled for: (a, b) basal keratinocyte markers Keratin-14, Integrin α6, p63, the epithelial marker E-cadherin, and the mesenchymal marker N-cadherin; (c, d) Keratin-14 (green), and the keratinocyte terminal differentiation markers Loricrin, Keratin-10 and Involucrin (red) with DAPI nuclear counter-stain (blue). (a, b) Note persistence of basal keratinocyte marker expression in both control and miR-203 tumors and metastases, as well as maintenance of an epithelial phenotype. (c, d) Note presence of terminally differentiated cells in both control and miR-203 tumors and metastases. Scale bars: (a, b) primary tumors 1 mm, lung metastases 250 μm; (c, d) 50 μm.

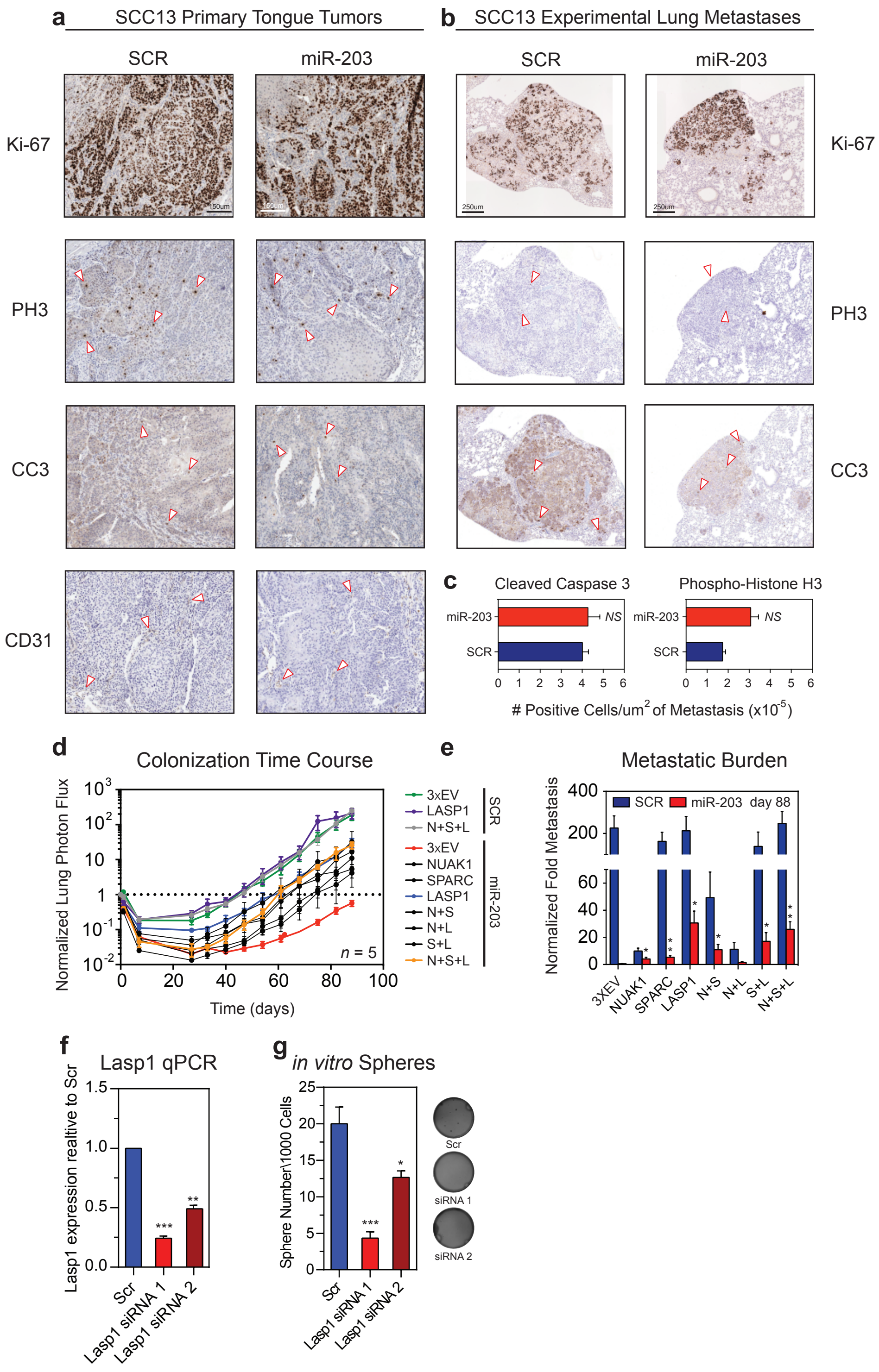

Figure S6. Immunohistochemical analysis of proliferation, apoptosis, and vascularity in SCC13 primary tongue tumors and experimental lung metastases and validation of role of LASP1 in anchorage independent growth of SCC13 cells. Related to Figure 5.

(a) and (b) Primary tongue xenograft tumors (a) and experimental lung metastases (b)

generated by SCC13 cells expressing control (SCR) or miR-203 were immunohistochemically stained for markers of proliferation (Ki-67 and PH3), apoptosis (CC3), and vascularity (CD31). Scale bars are 150 μm (tumors) and 250 μm (metastases).

(c) Quantification of CC3- and PH3-positive cells in control and miR-203 expressing SCC13 lung metastases. Data are presented as means (average of 15 individual metastases analyzed in one section per mouse, *n*=3 mice per group) ± SEM. A non-parametric Mann-Whitney test was used to calculate significance.

(d) Time course of experimental lung metastasis generated by control (SCR) SCC13 cells expressing 3xEV, LASP1 alone, or NUAK1+SPARC+LASP1 (N+S+L) as representative controls, and miR-203 SCC13 cells expressing all 8 cDNA combinations over 88 days (*n*=5 per group). Log_{10} y-axis, data are mean ± SEM with *n*=4-5 per group.

(e) Lung metastases formed by all 16 combinations of SCC13 cell lines at day 88. Data are presented as means ± SEM, *n*=4-5 per group. $^{*}P < 0.05$ and $^{**}P < 0.01$ calculated using a non-parametric Mann-Whitney test comparing miR-203 3xEV with miR-203 expressing one, two, or all three target gene cDNAs.

(f) qRT-PCR was used to assay for the expression levels of LASP1 in SCC13 cells transfected with either SCR control or two independent, LASP1 siRNAs (1 or 2). Expression was normalised to SCR. Data are mean ± SEM.

(g) *in vitro* sphere formation in an anchorage-independent soft-agar assay. The number of spheres was quantified after 3 weeks. Data are presented as mean ± SEM with representative wells shown.

Patient #	Age at diagnosis	Sample ID	Sample description
1	58	A	Primary skin SCC on left cheek with local skin metastasis. Patient suffered infraorbital metastasis infiltrating bone 26 months after diagnosis.
2	82	A	Primary skin SCC on left lower leg
2		B	Local skin metastasis on left knee in transit to lymph nodes suffered 14 months after diagnosis
2		C	Local skin metastasis on left knee following tumor progression post-chemo
3	78	A	Primary skin SCC on frontal head
3		B	Local skin metastasis in transit to lymph nodes
3		C	Local skin metastasis in transit to lymph nodes
3		D	Local skin metastasis in transit to lymph nodes
3		E	Local skin metastasis in transit to lymph nodes
3		F	Local skin metastasis in transit to lymph nodes
4	69	A	Primary skin SCC on left frontal head
4		B	Local skin metastasis in transit to lymph nodes
4		C	Local skin metastasis in transit to lymph nodes
5	81	A	Primary lower lip SCC
5		B	Primary lower lip SCC with potential right submandibular lymph node metastasis
6	75	A	Primary skin SCC on left second finger. Later detected 2/7 positive left axilla lymph nodes; 2 months later found distant metastasis to lungs and kidneys. Overall survival 24 months.
7	81	A	Primary skin SCC
7		B	Primary skin SCC
7		C	Ulcerated primary skin SCC on left temple involving subcutaneous metastasis in right preauricular lymph node
7		D	Local skin metastasis in transit to lymph nodes
7		E	Local skin metastasis in transit to lymph nodes
8	85	A	Primary skin SCC on right temple
8		B	Local skin metastasis in transit to lymph nodes
8		C	Local skin metastasis in transit to lymph nodes
9	80	A	Primary skin SCC above left front parietal lobe. Patient suffered metastasis to parotid gland and

			cervical lymph node 2 months after local spread.
9		B	Local skin metastasis in transit to lymph nodes
10	86	A	Primary skin SCC on right temple
10		B	Local skin metastasis
10		C	Local skin metastasis
11	90	A	Primary skin SCC on right frontal head
11		B	Local skin metastasis in transit to lymph nodes

Table S1. Clinical information of patients from whom matched skin and SCCs were resected and used for miRNA *in situ* hybridization. Related to Figure 2.

Supplemental Experimental Procedures

Cell Lines and Human Tissue

The human facial skin SCC line, SCC13, was obtained from ATCC. Primary human "SJG" cell lines were established from freshly resected HNSCC specimens of patients treated at Addenbrooke's Hospital, Cambridge. Primary normal human lip (strain LKa) and foreskin keratinocytes were obtained from surgical operations at the same hospital. Work with human material was carried out in compliance with the UK Human Tissue Act (2004) and approved by the National Research Ethics Service (08/H0306/30), or the German Medical Council, according to the recommendations of the local ethics committee, for tissue used in research. Appropriate informed consent was obtained from patients prior to their operation.

All cell lines were cultured at 37°C with 5% CO_2 in FAD medium (three parts DMEM medium, one part F12 medium and 10^{-4} M adenine, supplemented with 10% Fetal Bovine Serum Gold (PAA), 2 mM L-glutamine, 0.5 μg/ml hydrocortisone, 5 μg/ml insulin, 10^{-10} M cholera toxin, 10 ng/ml epidermal growth factor, 100 IU/l penicillin and 100 μg/l streptomycin (Gibco). HEK293T cells for lentiviral packaging were maintained in DMEM supplemented with 10% Fetal Bovine Serum, 100 IU/l penicillin and 100 μg/l streptomycin (Gibco). Cell lines passed mycoplasma testing.

For *in vitro* sphere forming assays, 1 ml of a 1% agar/FAD medium base layer was added to a 6-well culture plate (Corning) and 1,000 – 3,000 cells (depending on assay) were suspended in 1 ml 0.8% agar/FAD medium and seeded on top the solidified base layer. The cells were cultured for 21 days, stained with 0.01% Crystal Violet and imaged using a Bio-Rad XR+. For *in vitro* doxycycline induction

experiments, cells were grown in the presence of 1mg/ml doxycycline (filtered and diluted in PBS) for 3 days (Sigma).

Plasmid Construction

To generate miRNA-expression vectors, the human sequences encoding miR-15a, miR-26b, miR-125b-1, miR-203, miR-218-1 and miR-373, as well as up to 250 bp of upstream and downstream flanking genomic sequence, were PCR-amplified from normal human keratinocyte genomic DNA using Phusion High-Fidelity Polymerase (NEB) with 10% DMSO and buffers for GC-rich sequences. Relevant primer sequences for cloning are available in supplementary information. miRNA PCR products were verified by gel electrophoresis, purified using the QIAquick PCR Purification Kit (Qiagen), subcloned into the *XhoI* and *MluI* sites of the pGIPZ and/or pTRIPZ lentiviral expression vectors (Thermo), and sequence-verified. The pGIPZ and pTRIPZ lentiviral scrambled hairpin control plasmids were purchased from Open Biosystems. To knock down expression of miR-15a and miR-203, miRZip lentiviral expression vectors were purchased from System Biosciences.

Precision LentiORFs (pLOC backbone) encoding human LASP1 (clone ID PLOHS_100004169) and SPARC (clone ID PLOHS_100006045) cDNAs with their native stop codons were purchased from Thermo. An ORFeome Collaboration (pENTR223.1 backbone) encoding human NUAK1 cDNA (clone ID 100064197) with its native stop codon was purchased from Thermo. LeGO-iC2 (SFFV promoter, IRES-mCherry; Addgene plasmid 27345) and LeGO-iCer2 (SFFV promoter, IRES-Cerulean; Addgene plasmid 27346) were rendered Gateway-competent using the Gateway Vector Conversion System with One Shot *ccdB* Survival Cells (Invitrogen). These plasmids were used as empty vector controls. NUAK1 cDNA was then inserted into LeGO-iCer2 and SPARC cDNA into LeGO-iC2 using standard Gateway cloning techniques (Invitrogen) and sequence verified. A pLOC empty vector was generated by excising the LASP1 cDNA, blunting with Klenow, and ligating ends (NEB).

To generate psiCheck2-3'UTR reporter plasmids, relevant 3'UTR sequences were obtained from Ensembl, PCR amplified from normal human keratinocyte genomic DNA, verified by gel electrophoresis and gel purified when required, and subsequently cloned into the *XhoI* and *NotI* sites of psiCheck2 (Promega). Mutant UTRs were generated using a QuikChange XL Site-Directed Mutagenesis kit

(Agilent) with the following differences: extension time (2.5min/kb), 3% DMSO, and 100ng input DNA. All plasmids were sequenced verified.

A retrovirus expressing a yellow fluorescent protein and codon-optimized firefly luciferase (YFP-Luc) fusion protein was a generous gift from Dr. Scott Lyons.

Virus Production and Generation of Stable Cell Lines

pGIPZ, pTRIPZ, miRZip, LeGO, and pLOC lentiviral vectors were packaged using pMDLg/pRRE (Addgene plasmid 12251), pRSV-Rev (Addgene plasmid 12253), and pMD2.G (Addgene plasmid 12259). The YFP-luciferase vector was packaged using the FELIX system of pCMV-VSVg (Addgene pCI-VSVg) and pCPRDEnv (Addgene plasmid 1732). In all cases plasmids (25 ng total) were transfected into HEK293T cells using 50 ul jetPEI in 500 ul total NaCl solution. Serum-containing DMEM was used at all times. Viral supernatants were harvested after 48, 72, and 96 hours post-transfection, pooled, filtered through a 40 μm syringe-driven filter (Millipore), concentrated 100x using the Lenti-X Concentrator system (Clontech), and stored as aliquots at -80°C. Concentrated lentiviral particles were then used to transduce $2x10^5$ target cells in 6-well dishes in the presence of 8 μg/ml polybrene in complete FAD overnight. Successfully infected cells were selected 48 hours post-transduction with either 2 ug/ml Puromycin, 150 ug/ml Hygromycin B, or Blasticidin (10 ug/ml) (Sigma).

SCC13, SJG15, SJG27 and SCC25 cells were first infected with YFP-luciferase virus followed by miRNA or cDNA vectors. To generate cDNA combination cell lines, YFP-luciferase expressing SCC13 cells were first infected with LeGO-iCer2-NUAK1 or LeGO-iCer2, sorted for Cerulean positivity by flow cytometry, and placed back in culture. These cells were then infected with LeGO-iC2-SPARC or LeGO-iC2, sorted for Cerulean and mCherry positivity and cultured. These double-cDNA cells were then infected with pLOC-LASP1 or pLOC-EV and treated with Blasticidin to select for triple-cDNA expressing cells. To generate pTRIPZ cells, YFP-luciferase expressing SCC13 cells were infected with pTRIPZ-SCR or pTRIPZ-miR-203 and selected with puromycin. Surviving clones were pooled and either treated with doxycycline for 3 days or not. Flow cytometry was used to isolate those cells that robustly induced tRFP expression upon doxycycline treatment;

uninduced cells remained uniformly tRFP negative. All cell sorting was run using a FACS Aria SORP (BD Biosciences).

RNA Extraction and qRT-PCR Expression Analysis

We curated miRNA expression data from 12 previously published studies using either primary HNSCC samples or cell lines for miRNAs coordinately deregulated in at least 3 of 12 reports (49, 50, 51, 52, 53, 54, 55, 56, 57, 58, 59, 60) The output list of 20 miRNAs was refined down to 15 miRNAs by excluding miRNAs with well-documented roles in cancer at the time of study initiation (miR-21, miR-31, miR-155, and the let-7 family) (61). miR-199b was removed from our candidate list given that the multiplexed primer pool used to assay miRNA expression did not feature a primer for this miRNA.

Total RNA was isolated using the miRNeasy Mini Kit (Qiagen) for both miRNA and mRNA expression analysis. For miRNA expression analysis, 500 ng input RNA was reverse transcribed using the TaqMan Megaplex Human Primer Pool A v2.1 and TaqMan MicroRNA Reverse Transcription Kit as per the manufacturer's instructions (Applied Biosystems). Expression of the relevant mature miRNA species was quantified by real-time PCR using individual TaqMan MicroRNAs Assays (Applied Biosystems) with the U6 snRNA as a normalization control. For mRNA expression analysis, 250 ng input RNA was reverse transcribed using the SuperScript III First-Strand Synthesis system (Invitrogen) according to the manufacturer's instructions. SYBR Green- or pre-designed TaqMan probe-based real-time PCR (Applied Biosystems) was used to amplify genes of interest with GAPDH or 18S ribosomal RNA serving as normalization controls, respectively. Data were acquired and analyzed using an ABI Prism 7900HT Sequence Detection System (Applied Biosystems). Relevant primer sequences are available in supplemental information.

Microarray Experiments and Gene Set Enrichment Analysis

Total RNA was isolated in technical duplicates from YFP-luciferase expressing SCC13 and SJG15 cells stably infected in biological duplicates with either miR-203 or scrambled control hairpin using a miRNeasy Mini Kit (Qiagen). RNA quality was analyzed using a Bioanalyzer 2100 (Agilent) and the RNA Nano chip.

Gene expression analysis was carried out on Illumina Human HT12 version 4 arrays. All data analyses were carried out on R using Bioconductor (62) packages. Raw intensity data from the array scanner were processed using the BASH (63) and HULK algorithms as implemented in the *beadarray* package (64). Log2 transformation and quantile normalization of the data were performed across all sample groups. Differential expression analysis was carried out using the *limma* package (65). Differentially expressed genes were selected using a p-value cut-off of <0.05 after application of FDR correction for multiple testing applied globally to correct for multiple contrasts. Gene expression data has been deposited into NCBI GEO and is accessible under accession number GSE47028.

GSEA and Leading Edge Analysis was performed using GSEA v2.0 software (Broad Institute) (66). Genes were ranked according to fold change from highest to lowest in miR-203 expressing SJG15 compared to control, or BMP2/7-treated primary human keratinocytes compared to control (33; GSE34558). The median value of probe sets present more than once was used and statistical significance assessed using 1000 random permutations of the gene set. An FDR q-value < 0.2 was considered significant. Gene sets were obtained from the MSigDB database v3.1 (September 2012 release) or from the published datasets indicated in the main text. Other datasets used but not shown are the following: melanoma (67, 68, 69), breast (70), prostate cancer (71), and endometroid endometrial tumors (72).

Luciferase Reporter Assays

HEK293T cells were seeded in white-coated 96 well plates (Corning) at a density of 10,000 cells/well overnight. 10ng psiCheck2-3'UTR vector was co-transfected with 20nM miRNA mimic molecules (Dharmacon) using jetPRIME (Polyplus), and incubated for 48 hours. Cells were subsequently lysed and a Dual-Luciferase assay (Promega) was performed using a PHERAstar *FS* multi-mode microplate reader (BMG LabTech) and standard protocols. Normalized luminescent signal (i.e. *Renilla*/*Firefly* ratio) in miRNA mimic transfected wells was compared to values from negative control miRNA transfected wells.

Animal Studies and Non-Invasive in vivo Bioluminescent Imaging

All animal work was subject to Cancer Research UK ethical review and performed in accordance with an approved U.K. Government Home Office license. Aged-matched (minimum 6 weeks of age) immunocomprimized NOD/SCID/interleukin-2 gamma chain null (*Il2rg*$^{-/-}$) (NSG) mice (Jackson Labs; bred in-house) were inoculated via the tail vein with either 10^5 or $2.5x10^5$ SCC13 or SJG15 cells in 200 μL of PBS. For tongue xenografts, either 10^5 10^4, 10^3, 100 or 10 SCC13 cells in 50 μL of PBS were injected into the anterior dorsal mucosa of mice anaesthesized with isofluorane.

For bioluminescent imaging, mice were injected intraperitoneally with 200 μL D-luciferin dissolved in PBS (15mg/ml), anaesthetized with isofluorane, and imaged using a Xenogen IVIS 200 system 10 min after injection (PerkinElmer). Bioluminescent images were analyzed using tethered Living Image acquisition software (PerkinElmer). To produce BLI plots and associated graphs, total photon flux was calculated by drawing a region of interest across the thorax (experimental metastases) or head (tongue primary tumor growth) of each mouse individually in a supine position. This value was normalized to that obtained on the first day of scanning so that all mice began the experiment with an arbitrary starting BLI signal equal to 1.

Survival Analysis using Publically Available Microarray Datasets

NCBI GEO datasets GSE31056 (36) and GSE2379 (29) were downloaded as series matrix files of normalized expression values. Probe sets were matched to gene symbols for each platform. RNASeq (mRNA and miRNA) data from The Cancer Genome Atlas (TCGA) cohort of Head and Neck Cancer patients was downloaded as Level 3 data matrix files from the TCGA Data Portal available online (https://tcga-data.nci.nih.gov/tcga/). The BCGSC IlluminaHiSeq_miRNASeq (reads per million miRNA mapped) and the UNC IlluminaHiSeq_RNASeqV2 (normalized counts) data files were used for analysis. X-Tile software (73) was used to compute the optimal cutoff point for 2-population Kaplan-Meier analysis plotted using GraphPad Prism 6. Copy number aberrations called using gene- or cancer-centric GISTIC analysis of TCGA datasets were obtained through the TCGA Copy Number Portal available online (http://www.broadinstitute.org/tcga/home; 74).

Immunoblotting

For whole cell lysates, cells were grown to 70% confluency, washed twice with PBS, and harvested in RIPA buffer (Thermo Scientific). For secreted protein analysis, $5x10^6$ cells were seeded in 15 cm tissue culture places with 20ml serum-free FAD medium for 24 hours after which conditioned medium was harvested, filtered through a 0.45 µm syringe and concentrated using a 30 kDa Vivaspin 500 sample concentrator at full speed for 1 hour. Flow through was discarded and the remaining precipitate washed 3x for 30 minutes with 1M Tris-HCl (pH 8.0) followed by resuspension in RIPA buffer.

Protein lysates were sonicated for three 10-second cycles on medium power using a Biorupter (Diagenode) and sample concentration measured using a DC Protein Assay (Bio-Rad). 40 ug total protein per sample was separated on 4-12% polyacrylamide gradient gels, transferred to nitrocellulose membranes and detected using the following primary anti-human antibodies: LASP1 diluted 1:250 (Atlas HPA012072), NUAK1 diluted 1:250 (Atlas HPA027455), SPARC diluted 1:1,500 (Haemtech AON-5031), and GAPDH diluted 1:10,000 (Ambion AM4300). IRDye 800CW (800 nm channel) and 680LT (700 nm channel) secondary antibodies were used (LiCor). Western Blots were visualized using a Li-Cor Odyssey near infrared imager.

In vivo Intravasation

Total blood (w/heparin-lined syringe) was obtained by cardiac puncture under terminal anaesthesia from moribund mice that had received tongue xenografts of 10^4 SCC13-miR-203 and control cells. Total genomic DNA was extracted from 200 ul anti-coagulated whole blood per mouse using a QIAmp DNA Micro Kit (Qiagen) and diluted 1:20. The presence of circulating tumor cells was detected by SYBR Green qRT-PCR for the human repeat elements *AluJ* and *hLine1* (75), as well as the YFP-luciferase and Hygomycin genes integrated in luciferase-expressing cells.

Multi-Photon Confocal Microscopy

To quantitatively investigate *in vivo* extravasation potential, $2.5x10^5$ miR-203 and control SCC13 cells were tail-vein injected into NSG mice (*n*=3 per condition) and allowed to extravasate into the lungs. One hour prior to sacrifice, mice received an intravenous injection of 200 µl DyLight 594 labeled Tomato Lectin (*Lycopersicon*

Esculentum) (Vector Labs) to label the lung microvasculature. Whole lungs were surgically removed at necropsy after 30 hours of extravasation, washed in PBS, immobilized in an Ibidi μ-dish$^{35mm, high}$, and imaged as a wholemount with a Leica TCS SP5 confocal microscope equipped with a Chameleon Ultra laser (Coherent) for multi photon excitation using a 63x objective (HCX PL APO lambda blue 1.4 OIL). For excitation the laser was tuned to 835nm. An average of 25 lung areas were imaged per mouse, equating to 138 and 127 individual control and miR-203 expressing SCC13 cells per mouse, respectively. Images were processed with Volocity 3D Image Analysis 6.2 software (PerkinElmer) and Adobe Photoshop CS4.

miRNA in situ Hybridization

Formalin-fixed paraffin-embedded (FFPE) tissue blocks were sectioned at 10 μm on Fisher Superfrost slides and dried overnight at 45°C. After dewaxing and hydration through an ethanol series (100%, 70%, 50%), slides were incubated with 10ug/ml Proteinase K (Sigma Aldrich) for 8 min at 37°C. Slides were dehydrated in ethanol (70%, 90%, and 100%) and left to air dry. Double DIG-labeled LNA scrambled control and miR-203 probes (Exiqon) were diluted to 40nM in 2X hybridization buffer (Exiqon), denatured at 80°C for 4 min and chilled on ice. Probes were hybridized to tissue sections for 1 hour at 57°C using a hybridizer. Slides were put through a series of stringency washes with Saline-sodium citrate (SSC) and rinsed with PBS. Slides were then blocked for 30 min in antibody blocking buffer (10% sheep serum, 2% BSA in 1 X PBS) and incubated with anti-DIG-POD antibody (Roche; 1:200 in blocking buffer) for 1 hour at room temperature. Samples were washed in TNT buffer (0.1M Tris HCl pH 7.4, 0.15M NaCl, 0.05% Tween 20, 0.1% DEPC) and the Perkin Elmer TSA Cy3 kit reagents utilized to amplify and detect signal according to the manufacturer's protocol. Slides were mounted in Prolong Gold + DAPI and widefield fluorescence images of tissue sections were obtained with the Ariol automated microscope image capture system using filter sets for DAPI (nucleus) and Cy3 (miRNA) at fixed exposures.

Immunohistochemistry

FFPE specimens were de-waxed and rehydrated, using standard protocols, on the automated Leica ST5020. Antigen retrieval was done at 100°C in citrate buffer or

EDTA. The following primary antibodies were used: Ki67 (DAKO, clone MIB-1, 1:200), p63 (Novocastra, clone 7JUL, 1:50), EpCAM (Novocastra, clone VU-1D9, 1:100), Cytokeratin-14 (Novocastra, clone LL002, 1:20), Cytokeratin-5 (Novocastra, clone XM26, 1:100), E-Cadherin (DAKO, clone NCH-38, 1:25), SPARC (Haematologic Technologies, cat number AON-5031, 1:1500), Vimentin (Novocastra, clone SRL33, 1:400), N-Cadherin (Novocastra, clone IAR06, 1:100), Cleaved Caspase-3 (CC3) (Cell Signaling, cat number 9664, 1:100), Alpha 6 Integrin (Atlas, HPA012696, 1:140), Phospho-Histone H3 (PH3) (Ser10; Upstate, cat number 06-570, 1:500), GFP (Abcam, ab13970, 1;1000), CD31 (BD Pharmingen, clone MEC13.3, 1:100), LASP1 (Atlas, HPA012072, 1:500), NUAK1 (Atlas, HPA027455, 1:50), Keratin-10 (Covance, PRB-159P, 1:500), Loricrin (Covance, PRB-145P, 1:500), Involucrin (in-house, clone SY7, 1:500) and Keratin-14 (Covance, PRB-155P, 1:1000).

CC3, PH3, GFP, and CD31 were run on the LSAB system (Intense R) on the automated Bond Max (Leica Microsystems). All other antibodies were run on the polymer refine kit as per the manufacturer's instructions (Leica Microsystems).

Secondary antibodies (Jackson) were run at 1:250 in Bond diluent: biotinylated donkey anti-rat (ref. 712-065-153), biotinylated donkey anti-chicken (ref. 703-066-155), and biotinylated donkey anti-rabbit (ref. 711-065-152). The DAB Enhancer was used for all antibodies (Leica, ref. AR9432), as was the Avidin/Biotin blocking kit (Vector, ref. SP-2001).

Post-IHC hydration and clearing were performed on the automated Leica ST5020 and slides were mounted on Leica's CV5030. The automated ScanScope Digital Slide Scanner and software (Aperio) were used to image staining.

Graphing and Statistical Analysis

All graphs were generated using GraphPad Prism 6 and Adobe Illustrator CS4 software. Data are mean ± standard error of the mean (SEM). An unpaired two-tailed Student's t-test (*in vitro* experiments) or a non-parametric Mann-Whitney test (*in vivo* mouse experiments) was used for comparisons, with $P < 0.05$ considered significant. The Log-rank (Mantel-Cox) test was used to compare survival curves and compute Hazard Ratios, and a Fisher's exact test used to compare human cohorts on the basis of clinical characteristics.

Supplemental References

(49) Avissar, M., Christensen, B.C., Kelsey, K.T., and Marsit, C.J. (2009). MicroRNA expression ratio is predictive of head and neck squamous cell carcinoma. Clin. Cancer Res. *15*, 2850–2855.

(50) Childs, G., Fazzari, M., Kung, G., Kawachi, N., Brandwein-Gensler, M., McLemore, M., Chen, Q., Burk, R.D., Smith, R.V., Prystowsky, M.B., et al. (2009). Low-level expression of microRNAs let-7d and miR-205 are prognostic markers of head and neck squamous cell carcinoma. Am. J. Pathol. *174*, 736–745.

(51) Hebert, C., Norris, K., Scheper, M.A., Nikitakis, N., and Sauk, J.J. (2007). High mobility group A2 is a target for miRNA-98 in head and neck squamous cell carcinoma. Mol. Cancer *6*, 5.

(52) Henson, B.J., Bhattacharjee, S., O'Dee, D.M., Feingold, E., and Gollin, S.M. (2009). Decreased expression of miR-125b and miR-100 in oral cancer cells contributes to malignancy. Genes Chromosomes Cancer *48*, 569–582.

(53) Hui, A.B.Y., Lenarduzzi, M., Krushel, T., Waldron, L., Pintilie, M., Shi, W., Perez-Ordonez, B., Jurisica, I., O'Sullivan, B., Waldron, J., et al. (2010). Comprehensive MicroRNA profiling for head and neck squamous cell carcinomas. Clinical Cancer Research: An Official Journal of the American Association for Cancer Research *16*, 1129–1139.

(54) Kikkawa, N., Hanazawa, T., Fujimura, L., Nohata, N., Suzuki, H., Chazono, H., Sakurai, D., Horiguchi, S., Okamoto, Y., and Seki, N. (2010). miR-489 is a tumor-suppressive miRNA target PTPN11 in hypopharyngeal squamous cell carcinoma (HSCC). Br. J. Cancer *103*, 877–884.

(55) Kimura, S., Naganuma, S., Susuki, D., Hirono, Y., Yamaguchi, A., Fujieda, S., Sano, K., and Itoh, H. (2010). Expression of microRNAs in squamous cell carcinoma of human head and neck and the esophagus: miR-205 and miR-21 are specific markers for HNSCC and ESCC. Oncol. Rep. *23*, 1625–1633.

(56) Kozaki, K., Imoto, I., Mogi, S., Omura, K., and Inazawa, J. (2008). Exploration of tumor-suppressive microRNAs silenced by DNA hypermethylation in oral cancer. Cancer Res. *68*, 2094–2105.

(57) Ramdas, L., Giri, U., Ashorn, C.L., Coombes, K.R., El-Naggar, A., Ang, K.K., and Story, M.D. (2009). miRNA expression profiles in head and neck squamous cell carcinoma and adjacent normal tissue. Head Neck *31*, 642–654.

(58) Tran, N., McLean, T., Zhang, X., Zhao, C.J., Thomson, J.M., O'Brien, C., and Rose, B. (2007). MicroRNA expression profiles in head and neck cancer cell lines. Biochem. Biophys. Res. Commun. *358*, 12–17.

(59) Wald, A.I., Hoskins, E.E., Wells, S.I., Ferris, R.L., and Khan, S.A. (2011). Alteration of microRNA profiles in squamous cell carcinoma of the head and neck cell lines by human papillomavirus. Head & Neck *33*, 504–512.

(60) Wong, T.-S., Liu, X.-B., Wong, B.Y.-H., Ng, R.W.-M., Yuen, A.P.-W., and Wei, W.I. (2008). Mature miR-184 as Potential Oncogenic microRNA of Squamous Cell Carcinoma of Tongue. Clin. Cancer Res. *14*, 2588–2592.

(61) Ventura, A., and Jacks, T. (2009). MicroRNAs and cancer: short RNAs go a long way. Cell *136*, 586–591.

(62) Gentleman, R.C., Carey, V.J., Bates, D.M., Bolstad, B., Dettling, M., Dudoit, S., Ellis, B., Gautier, L., Ge, Y., Gentry, J., et al. (2004). Bioconductor: open software development for computational biology and bioinformatics. Genome Biology *5*, R80.

(63) Cairns, J.M., Dunning, M.J., Ritchie, M.E., Russell, R., and Lynch, A.G. (2008). BASH: a tool for managing BeadArray spatial artefacts. Bioinformatics (Oxford, England) *24*, 2921–2922.

(64) Dunning, M.J., Smith, M.L., Ritchie, M.E., and Tavaré, S. (2007). beadarray: R classes and methods for Illumina bead-based data. Bioinformatics *23*, 2183–2184.

(65) Smyth, G.K. (2004). Linear models and empirical bayes methods for assessing differential expression in microarray experiments. Stat Appl Genet Mol Biol *3*, Article3.

(66) Subramanian, A., Tamayo, P., Mootha, V.K., Mukherjee, S., Ebert, B.L., Gillette, M.A., Paulovich, A., Pomeroy, S.L., Golub, T.R., Lander, E.S., et al.

(2005). Gene set enrichment analysis: A knowledge-based approach for interpreting genome-wide expression profiles. PNAS *102*, 15545–15550.

(67) Winnepenninckx, V., Lazar, V., Michiels, S., Dessen, P., Stas, M., Alonso, S.R., Avril, M.-F., Ortiz Romero, P.L., Robert, T., Balacescu, O., et al. (2006). Gene expression profiling of primary cutaneous melanoma and clinical outcome. J. Natl. Cancer Inst. *98*, 472–482.

(68) Jaeger, J., Koczan, D., Thiesen, H.-J., Ibrahim, S.M., Gross, G., Spang, R., and Kunz, M. (2007). Gene expression signatures for tumor progression, tumor subtype, and tumor thickness in laser-microdissected melanoma tissues. Clin. Cancer Res. *13*, 806–815.

(69) Alonso, S.R., Tracey, L., Ortiz, P., Pérez-Gómez, B., Palacios, J., Pollán, M., Linares, J., Serrano, S., Sáez-Castillo, A.I., Sánchez, L., et al. (2007). A high-throughput study in melanoma identifies epithelial-mesenchymal transition as a major determinant of metastasis. Cancer Res. *67*, 3450–3460.

(70) Ramaswamy, S., Ross, K.N., Lander, E.S., and Golub, T.R. (2003). A molecular signature of metastasis in primary solid tumors. Nat. Genet. *33*, 49–54.

(71) Chandran, U.R., Ma, C., Dhir, R., Bisceglia, M., Lyons-Weiler, M., Liang, W., Michalopoulos, G., Becich, M., and Monzon, F.A. (2007). Gene expression profiles of prostate cancer reveal involvement of multiple molecular pathways in the metastatic process. BMC Cancer *7*, 64.

(72) Bidus, M.A., Risinger, J.I., Chandramouli, G.V.R., Dainty, L.A., Litzi, T.J., Berchuck, A., Barrett, J.C., and Maxwell, G.L. (2006). Prediction of lymph node metastasis in patients with endometrioid endometrial cancer using expression microarray. Clin. Cancer Res. *12*, 83–88.

(73) Camp, R.L., Dolled-Filhart, M., and Rimm, D.L. (2004). X-Tile A New Bio-Informatics Tool for Biomarker Assessment and Outcome-Based Cut-Point Optimization. Clin Cancer Res *10*, 7252–7259.

(74) Mermel, C.H., Schumacher, S.E., Hill, B., Meyerson, M.L., Beroukhim, R., and Getz, G. (2011). GISTIC2.0 facilitates sensitive and confident localization of the targets of focal somatic copy-number alteration in human cancers. Genome Biol. *12*, R41.

(75) Gorges, T.M., Schiler, J., Schmitz, A., Schuetzmann, D., Schatz, C., Zollner, T.M., Krahn, T., von Ahsen, O. (2012) Cancer therapy monitoring in xenografts by quantitative analysis of circulating tumor DNA. Biomarkers. *17*, 498-506.
